# Predictive value of serum biomarkers for survival in melanoma: a systematic review and meta-analysis

**DOI:** 10.1038/s41598-026-54347-w

**Published:** 2026-06-08

**Authors:** Lili Gulyás, Fanni Adél Meznerics, Bence Szabó, Noémi Nóra Varga, Lajos Vince Kemény, Norbert Kiss, Tamara Érseki, Péter Hegyi, András Bánvölgyi, Kende Kálmán Lőrincz

**Affiliations:** 1 Department of Dermatology, Venereology and Dermatooncology, Faculty of Medicine, Semmelweis University, H-1085, Mária utca 41., Budapest, Hungary; 2 Centre for Translational Medicine, Semmelweis University, Budapest, Hungary; 3 Institute for Translational Medicine, Medical School, University of Pécs, Pécs, Hungary; 4 Institute of Pancreatic Diseases, Semmelweis University, Budapest, Hungary; 5 Department of Physiology, Faculty of Medicine, Semmelweis University, Budapest, Hungary

**Keywords:** Melanoma, Serum biomarkers, LDH, NLR, ctDNA, Meta-analysis, Biomarkers, Cancer, Immunology, Oncology

## Abstract

Malignant melanoma is responsible for most skin cancer–related deaths due to its unpredictable behavior. Serum biomarkers have been widely investigated to improve prognostic assessment, yet their clinical utility remains inconclusive. This systematic review and meta-analysis evaluated the prognostic significance of serum biomarkers in melanoma. Following PRISMA guidelines and a registered protocol (PROSPERO: CRD42023486532), PubMed, EMBASE, and CENTRAL were searched for studies assessing biomarkers and survival outcomes. Univariate analyses revealed that elevated levels of LDH (HR 2.29, 95%-CI:1.97–2.67), S100B (HR 2.52, 95%-CI:1.59–3.99), circulating tumor DNA (ctDNA) (HR 2.61, 95%-CI:1.90–3.58), neutrophil-to-lymphocyte ratio (NLR) (HR 2.34, 95%-CI:1.86–2.93), and interleukin-6 (HR 3.11, 95%-CI:2.44–3.96) were significantly associated with reduced overall survival. Similarly, higher levels of LDH (HR 2.04, 95%-CI:1.68–2.47), S100B (HR 1.94, 95%-CI:1.39–2.70), ctDNA (HR 2.57, 95%-CI:1.95–3.39), and NLR (HR 2.38, 95%-CI:1.52–3.73) predicted shorter progression-free survival. These associations persisted in multivariable-adjusted analyses for LDH, ctDNA, NLR, IL-6, S100B, and CRP supporting their predictive relevance. LDH remains a reliable and cost-effective biomarker, while NLR may provide complementary prognostic information in patients receiving immune checkpoint inhibitors. Emerging biomarkers such as ctDNA demonstrate promising prognostic potential, but further evaluation is required before routine clinical implementation.

**Supplementary Information:**

The online version contains supplementary material available at 10.1038/s41598-026-54347-w.

## Introduction

Malignant melanoma accounts for an estimated 8430 deaths/year in the US from skin cancer, and exhibits a highly variable and aggressive clinical course due to its unpredictable metastatic potential^1^. Globally, cutaneous melanoma accounted for approximately 325,000 new cases and 57,000 melanoma-related deaths in 2020^2^. Between 2017 and 2021, more than 606,000 new cases were diagnosed in Europe, with marked geographical distribution, including higher incidence and mortality in Northern Europe and a higher mortality-to-incidence ratio in Eastern Europe^3^.

In the early stages, the 5-year survival is 94%, but it significantly decreases in advanced cases to 29.8%, and malignant melanoma is responsible for the majority of skin-cancer-related deaths^4^. In such instances, the expected outcome of the disease influences therapeutic decisions and care protocols; however, predicting patient outcomes remains a major challenge. Current guidelines emphasize staging, imaging, and mutational status for treatment planning, and monitoring melanoma patients, while among serum biomarkers, only LDH is routinely recommended as a predictive tool in metastatic melanoma^5^.

To predict disease course precisely, it is crucial to identify and investigate biomarkers that correlate with disease progression and patient survival. In recent years, several serum biomarkers have been investigated to assess disease progression, monitor treatment efficacy, and predict patient survival; however, their predictive performance is ambiguous^6,7^. There are widely accepted markers such as LDH and S100B. LDH is an easily measurable serum enzyme marker released from tumorous cells; however, it is not specific to melanoma^8^. It has been recognized as a strong predictor of survival in metastatic melanoma and included as a prognostic factor in the American Joint Committee on Cancer (AJCC) staging system^7^. S100B, secreted by melanocytes, involved in cell growth and differentiation, emerged as an independent prognostic biomarker, with elevated serum levels correlating with reduced survival in melanoma patients^9^. However, its clinical applicability appears to be limited in certain subtypes, such as uveal melanoma, where S100B levels do not consistently increase with tumor progression, especially in S100-negative tumors^10^. While these conventional biomarkers provide valuable clinical insights, their predictive performance is limited. This limitation has led to growing interest in novel serum biomarkers. Among these biomarkers, there are circulating tumor-derived biomarkers, including circulating tumor DNA (ctDNA) and circulating tumor cells (CTCs), reflecting the genetic and cellular characteristics of melanoma. ctDNA holds promise for non-invasive mutation profiling, monitoring treatment response, and detecting minimal residual disease. Another group of biomarkers are inflammatory and immunological markers, such as interleukin-6 (IL-6), neutrophil-to-lymphocyte ratio (NLR), and C-reactive protein (CRP), which serve as indicators of the systemic inflammatory response, with higher levels generally correlating with adverse outcomes and reduced efficacy of immunotherapy. While novel serum biomarkers showed promising results, their clinical utility remained debatable, highlighting the need for a comprehensive evaluation of their clinical utility^11,12^. Therefore, this study aimed to compare the predictive value of conventional and novel serum biomarkers regarding patient survival and disease progression^13,14^.

## Methods

A systematic review and meta-analysis was conducted following the recommendations of the Preferred Reporting Items for Systematic Review and Meta-Analysis (PRISMA) 2020 Guidelines and the Cochrane Handbook (version 6.4)^15,16^. The protocol was registered on PROSPERO (registration number: CRD42023486532). No modifications were made to the protocol.

### Search strategy, selection process

A systematic literature search was performed in three medical databases: PubMed (MEDLINE), EMBASE, and Cochrane Central Register of Controlled Trials (CENTRAL) on November 25, 2023. A preliminary search was conducted to identify serological biomarkers investigated in malignant melanoma. A predefined search key was used with three domains. The detailed search strategy – including the search key and the number of records – is provided in the supplementary material. Original studies using statistical methods to demonstrate or validate models designed for predicting melanoma outcomes of interest, including clinical trials and prospective and retrospective cohort studies were eligible for inclusion in this systematic review and meta-analysis, provided they met the following Population-Exposure-Outcome (PEO) framework: (P) melanoma patients, (E) serum biomarkers (O) death, disease progression, survival. Case reports, case series, letters, editorials, systematic reviews, meta-analyses, and studies without valuable or suitable data were excluded. After conducting the systematic search, the retrieved articles were imported into a reference management program (EndNote 21, Clarivate Analytics, Philadelphia, PA, USA)^17^. Duplicates were removed automatically and manually, based on overlapping publication years, authors, and titles. After duplicate removal, two independent reviewers (L.G. and N.N.V.) screened the articles by title and abstract, followed by full-text review, using the Rayyan Systems software tool (Cambridge, MA, USA)^18^. A third independent reviewer resolved disagreements (F.A.M.).

### Data extraction and synthesis

A standardized data collection sheet was used following the consensus of methodological and clinical experts. Two independent review authors (L.G. and N.N.V.) performed the data extraction. The following data were collected: title, first author, year of publication, country, study type and period, patient characteristics, main study findings, follow-up time, median survival time, Hazard Ratios of survival outcomes and their 95% confidence intervals, and studied biomarkers and their cut-off values. The investigated outcomes were overall survival (OS), which was defined as the time from treatment initiation to death; progression-free survival (PFS), which was defined as the time from initiation to therapy to radiological progression (defined by either RECIST 1.1 or irRECIST 1.1); melanoma specific survival or disease specific survival (DSS), which was defined from start of therapy to death due to melanoma event or last follow-up; disease-specific survival after progression (DSSap), calculated from radiologically ascertained PD to death due to melanoma or last follow-up; recurrence-free survival (RFS); and distant metastasis recurrence-free survival (DM-RFS).

Two independent authors (L.G. and N.N.V.) performed the risk of bias assessment with the QUIPS (Quality in Prognosis Studies) tool following the recommendation of the Cochrane Collaboration^19^. Conflicts were resolved by mutual agreement. Evidence of publication bias was identified through visual inspection of funnel plots and confirmed by Egger’s test.

### Statistical analysis

For time-to-event data hazard ratios (HR) were reported with 95% confidence intervals (CI). IPD or the published HR and its 95% CI or log-rank test statistics were used for the calculations. To calculate pooled HRs, the logarithm of HRs and their SE values were calculated from the available data following the methodology of Tierney et al.^20^. Inverse variance weighting method was used on a logarithmic scale to calculate the pooled estimates, and the restricted maximum-likelihood estimator was used with the Q profile method for confidence intervals^21,22^.

Heterogeneity was assessed by Higgins and Thompson I^2^ statistics and variance measure (τ^2^)^23^. The R software^24^ with the meta^25^ and metafor^26^ packages was used for the analyses. Where authors reported a negative correlation between the biomarkers and patient survival, calculations were based on reciprocated values.

Results were considered statistically significant if the pooled CI did not contain the null effect value. The findings related to the meta-analysis were summarized on forest plots. Where the study number was sufficiently large and not too heterogeneous, the prediction intervals (i.e., the expected range of effects of future studies) of results were also reported.

Univariable HRs were pooled for all serum biomarkers with data available from at least three independent studies. For biomarkers supported by a limited number of studies, pooled estimates were considered exploratory and interpreted with caution.

Pairwise differences between subgroups were assessed using meta-regression with subgroup included as a categorical moderator. Following model estimation, Wald-type contrasts were constructed to compare all pairs of subgroup-specific pooled effects. Contrast estimates and their standard errors were derived from the regression coefficient vector and its variance–covariance matrix, and statistical significance was evaluated using normal approximation. Two-sided 95% confidence intervals were calculated, and p-values were adjusted for multiple comparisons using the Benjamini–Hochberg method.

Where sufficient data were available, additional meta-analyses were conducted using multivariable-adjusted HRs to assess whether observed associations persisted after adjustment for established clinical prognostic factors. Multivariable-adjusted analyses were performed separately for each survival and were restricted to biomarkers reported in at least ten studies to ensure model stability. For biomarkers supported by fewer than ten studies, forest plots were used for descriptive visualization of effect estimates, and corresponding pooled results were interpreted as exploratory. In the full additive multivariate meta-analysis models, the following variables were included: WHO-region, therapy, stage, cut-off value. The model selection for the best followed a backward stepAIC approach, in which non-significant predictors were sequentially dropped from the model while the corrected AIC value was monitored. If the AIC value decreased by at least 2, the reduced model was deemed better. This approach was continued until the AIC value was minimized, or until all predictors were dropped from the model. Model fit was evaluated by examining the normality of residuals using QQ-plots, by comparing the observed and fitted values, and by examining how the spread of residuals behaves throughout the range of observed values. These diagnostic plots are presented in the Supplementary Material (see Supplementary Figure S70-S93).

In those cases when a categorical predictor remained significant in the final model, model predictions with point and interval estimates for the HR values were given for all levels of the categorical predictor. In those cases, when the continuous cut-off predictor was significant in the final model, predictions were made for predefined cut-off values derived from established thresholds in the literature, namely 250 U/L for LDH, 5 for NLR, and 5 pg/mL for IL-6^27–29^. Due to heterogeneity in covariate selection across studies, adjustment sets were not harmonized. Biomarkers not meeting the predefined study number threshold were not subjected to multivariable pooling.

## Results

### Search, selection, and basic characteristics of included studies

**Fig. 1:**
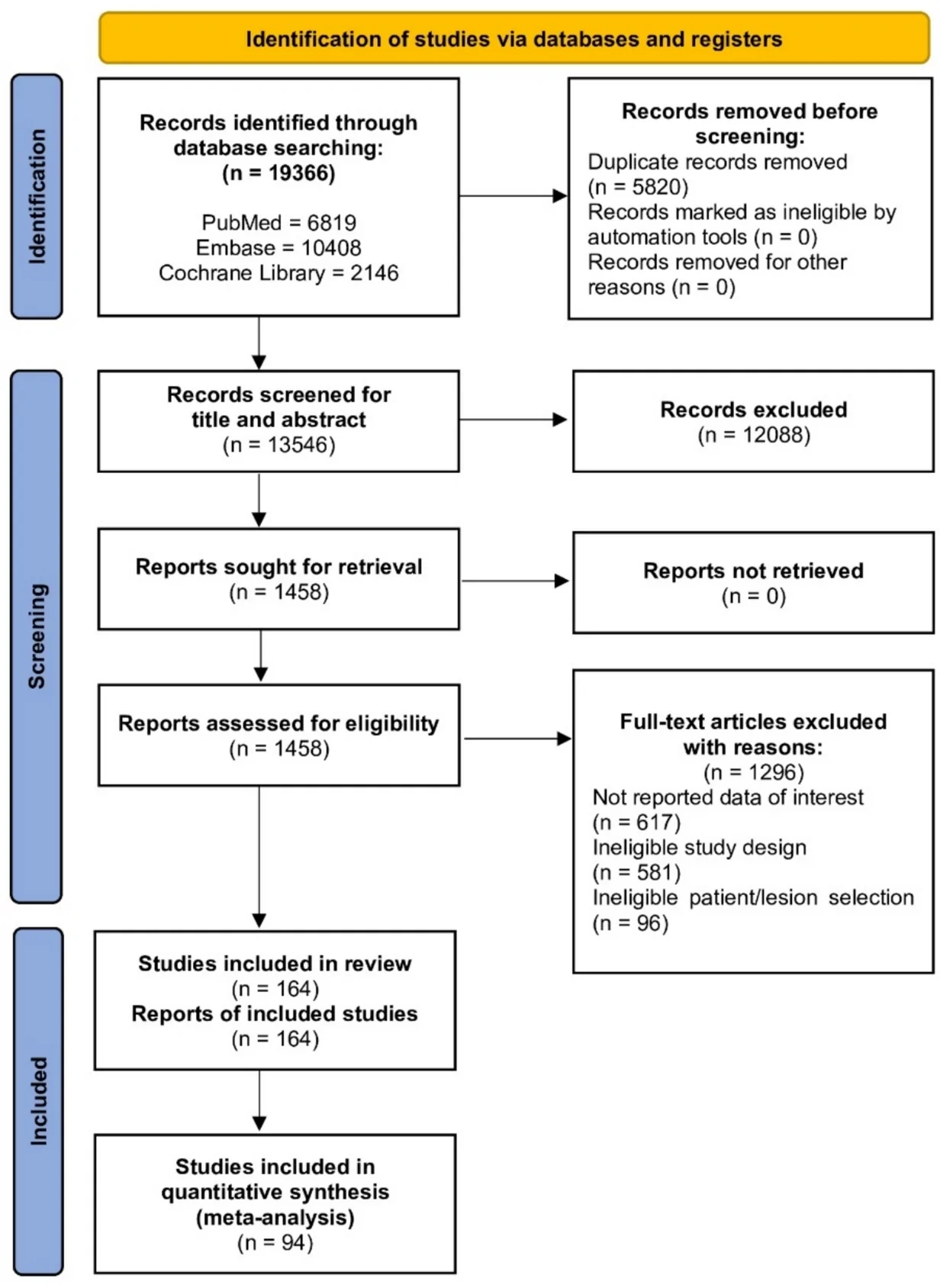
Preferred reporting in systematic reviews and meta-analyses (PRISMA) flowchart showing the selection process.

The systematic search yielded a total of 19,366 results. After duplicate removal, 13,546 articles were screened. After title, abstract, and full-text selection, 164 articles met the inclusion criteria. Of 164 included articles, 94 were subjected to quantitative analysis^30–123^, while the remaining studies were assessed qualitatively in the systematic review^124–193^. The detailed selection process is visualized in Fig. 1Ref.^15^. The basic study characteristics of the included articles and basic patient data are outlined in Supplementary Table S1.

### Quantitative analysis

#### Overall survival (OS)

##### Univariable analyses

**Fig. 2:**
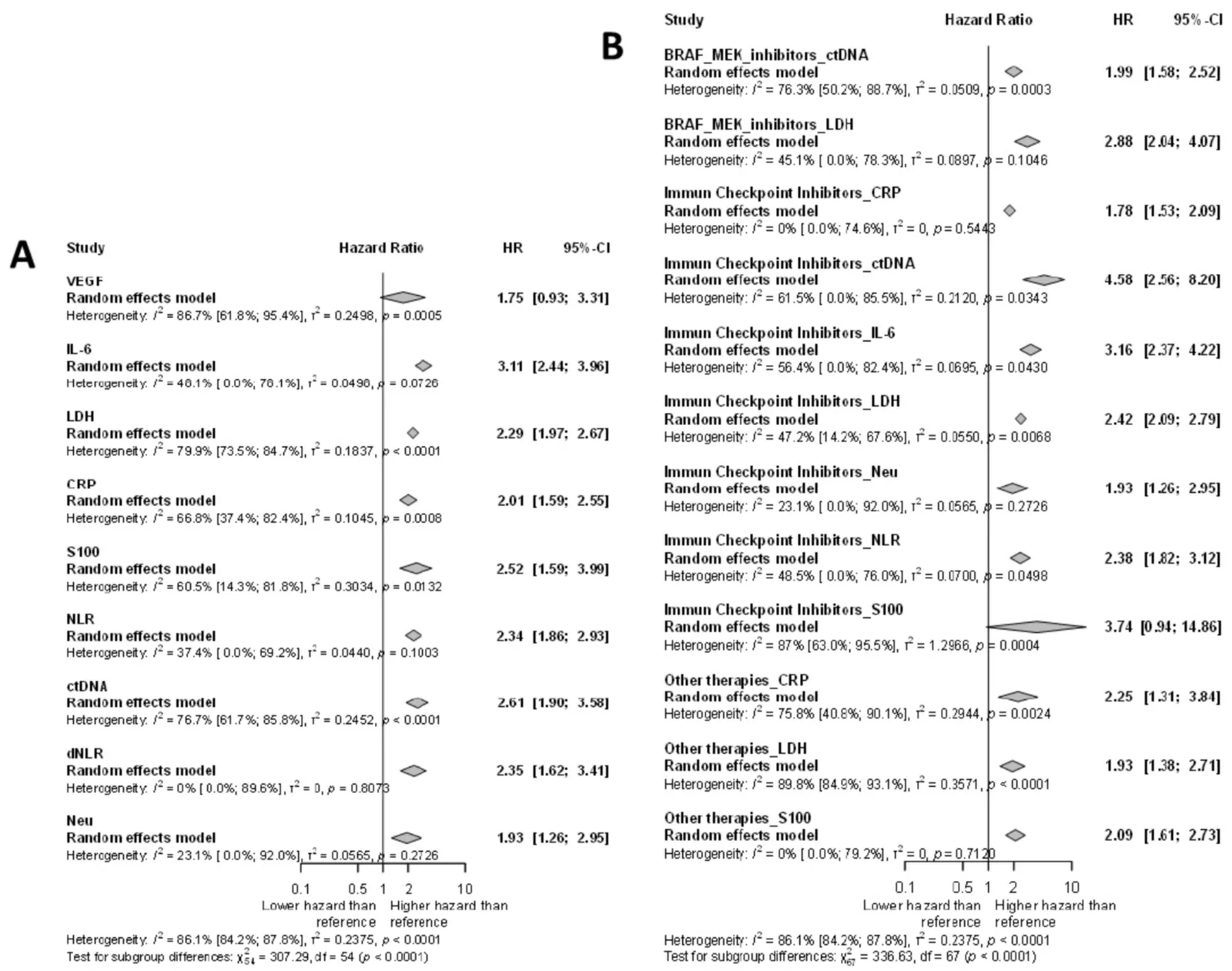
(**A**) Forest plot of univariate Hazard Ratios (HR) of OS and baseline serum biomarker level associations in melanoma patients, regardless of therapeutic agent. (**B**) Forest plot of univariate Hazard Ratios (HR) of OS and baseline serum biomarker level associations in melanoma patients subgrouped based on therapeutic agent. CI: confidence interval; CRP: C-reactive protein; ctDNA: circulating tumor DNA; dNLR: derived neutrophil-to-lymphocyte ratio; HR: hazard ratio; IL-6: interleukin-6; LDH: lactate dehydrogenase; Neu: neutrophils; NLR: neutrophil-to-lymphocyte ratio; S100: S100 calcium-binding protein; VEGF: vascular endothelial growth factor.

The following analyses show the results of different serum biomarker levels associated with OS. Elevated baseline levels of LDH (HR 2.29; 95% CI 1.97–2.67; I^2^ = 79.9%), S100B (HR 2.52; 95% CI 1.59–3.99; I^2^ = 60.5%), ctDNA (HR 2.61; 95% CI 1.90–3.58; I^2^ = 76.7%), IL-6 (HR 3.11; 95% CI 2.44–3.96; I^2^ = 48.1%), NLR (HR 2.34; 95% CI 1.86–2.93; I^2^ = 37.4%), dNLR (HR 2.35; 95% CI 1.62–3.41; I^2^ = 0%), neutrophil count (Neu) (HR 1.93; 95% CI 1.26–2.95; I^2^ = 23%) and CRP (HR 2.01; 95% CI 1.59–2.55; I^2^ = 66.8%) were associated with decreased OS. No significant differences were observed among the prognostic associations of the aforementioned individual biomarkers (see Supplementary Table S2). Elevated VEGF levels were not associated with decreased OS (HR 1.75; 95% CI 0.93–3.31; I^2^ = 86.7%). (see Fig. 2A).

The subgroup analysis stratified by therapeutic agent (Fig. 2B) contained three groups: solely BRAF/MEK inhibitors (BRAFMEKi) or immune checkpoint inhibitors (ICI) and further cytostatic and combination therapies labeled as “other therapies” (OT) (Fig. 2B). In the ICI and BRAFMEKi group, data involved stage III-IV patients, whereas in the OT group, patients with stage I-IV disease were included. BRAFMEKi-treated patients with elevated levels of LDH (HR 2.88; 95% CI 2.04–4.07; I^2^ = 45.1%) and ctDNA (HR 1.99; 95% CI 1.58–2.52; I^2^ = 76.3%) were associated with decreased OS. ICI-treated patients with elevated levels of LDH (HR 2.42; 95% CI 2.09–2.79; I^2^ = 47.21%), ctDNA (HR 4.58; 95% CI 2.56–8.20; I^2^ = 61.5%), IL-6 (HR 3.16; 95% CI 2.37–4.22; I^2^ = 56.4%), NLR (HR 2.38; 95% CI 1.82–3.12; I^2^ = 48.5%), Neu (HR 1.93; 95% CI 1.26–2.95; I^2^ = 23.1%) and CRP (HR 1.78; 95% CI 1.53–2.09; I^2^ = 0%) were associated with decreased OS. Elevated S100B level showed a trend towards decreased OS; however, the association was not statistically significant in ICI-treated patients (HR 3.74; 95% CI 0.94–14.86; I^2^ = 97%). In the OT group, elevated levels of LDH (HR 1.93; 95% CI 1.38–2.71; I^2^ = 89.8%), S100B (HR 2.09; 95% CI 1.61–2.73; I^2^ = 0%) and CRP (HR 2.25; 95% CI 1.31–3.84; I^2^ = 75.8%) were associated with decreased OS. Pairwise analysis of the biomarkers revealed no statistically significant differences in their prognostic associations across therapeutic subgroups. In ICI-treated patients, ctDNA and IL-6 were associated with worsened OS of patients compared to CRP. (see Supplementary Table S2-S3). Elevated biomarker levels were associated with worsened OS irrespective of disease stage (subgrouped as stage III-IV vs. IV), with no clinically meaningful subgroup differences(See Supplementary Figure S3-S14).

Patients with elevated follow-up levels on therapy of ctDNA (HR 3.11; 95% CI 1.27–7.62) and NLR (HR 1.13; 95% CI 1.05–1.22) also had significantly worse OS (see Supplementary Figure S15). The detailed forest plots for baseline and follow-up results related to the biomarkers are presented in Supplementary Figures S1-S2-S15.

##### Multivariable-adjusted analyses

Multivariable-adjusted hazard ratios were available from 52 studies for LDH, 9 for S100, 8 for ctDNA, 10 for IL-6, 12 for NLR, and 12 for CRP.

In the cases of the models fit for LDH and IL-6, meta regression identified cut-off values as significant moderators. The coefficients and p-values for the biomarkers are reported below: LDH (β = 0.0008, p = 0.023), IL-6 (β = 0.0591, p = 0.004) (see Supplementary Table S4 –S5). In such cases, predictions were made for predefined cut-off values, and the reporting of the HR for a given biomarker was based on these predictions. The cut-off for LDH was 250 U/L, for IL-6 it was 5 pg/mL.

**Fig. 3:**
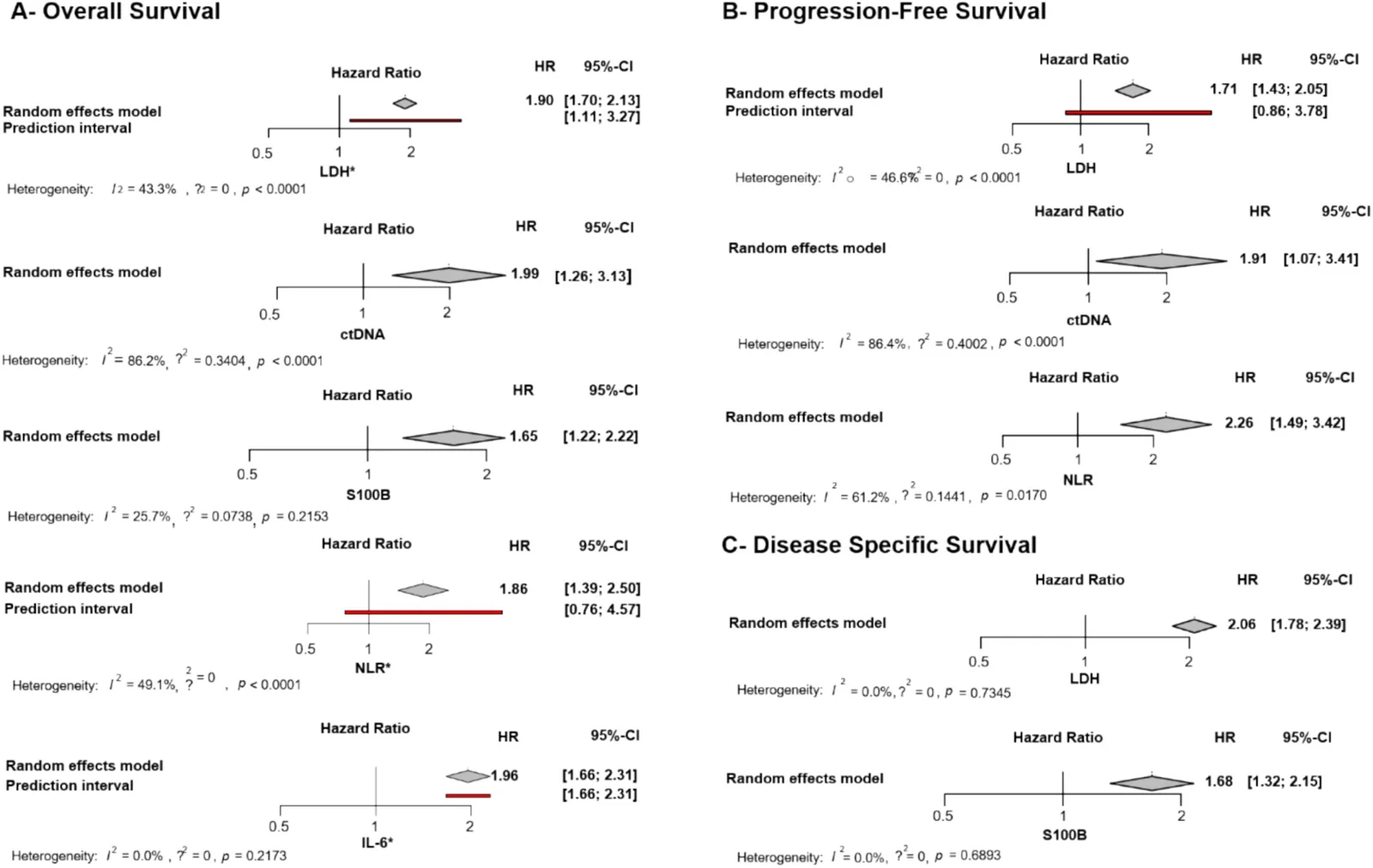
Pooled results of multivariate Hazard Ratios (HR) and multivariate model estimates for baseline serum biomarker level associations with survival outcomes in different models. Panels show pooled hazard ratios for (**A**) OS, (**B**) PFS, and (**C**) DSS. Asterisks (*) indicate results derived from multivariate models. LDH: lactate dehydrogenase; ctDNA: circulating tumor DNA; S100B: S100 calcium-binding protein B; NLR: neutrophil-to-lymphocyte ratio; IL-6: interleukin-6; HR: hazard ratio; CI: confidence interval.

Elevated LDH (HR 1.90, 95% CI 1.70–2.13; I^2^ = 43.3%), IL-6 levels (HR 1.96, 95% CI 1.66–2.31; I^2^ = 0%) and NLR (HR 1.86, 95% CI 1.39–2.50; I^2^ = 49.1%) remained significantly associated with worse OS (see Fig. 3A). Elevated ctDNA (HR 1.99, 95% CI 1.26–3.13; I^2^ = 86.2%) and S100B (HR 1.65, 95% CI 1.22–2.22; I^2^ = 25.7%) levels were significantly associated with worse OS in a pooled analysis of multivariate HRs (see Fig. 3A and Supplementary Figures S16-S20).

Meta-regression analyses identified treatment category as a significant moderator of the association between CRP and OS. Elevated CRP levels were associated with worse OS in patients receiving ICIs (HR 1.39, 95% CI 1.20–1.62; I^2^ = 0%), with a stronger association observed in patients treated with other therapies (HR 2.35, 95% CI 1.93–2.86; I^2^ = 0%) (see Supplementary Figure S21 and Supplementary Table S7).

#### Progression-free survival (PFS)

##### Univariable analyses

**Fig. 4:**
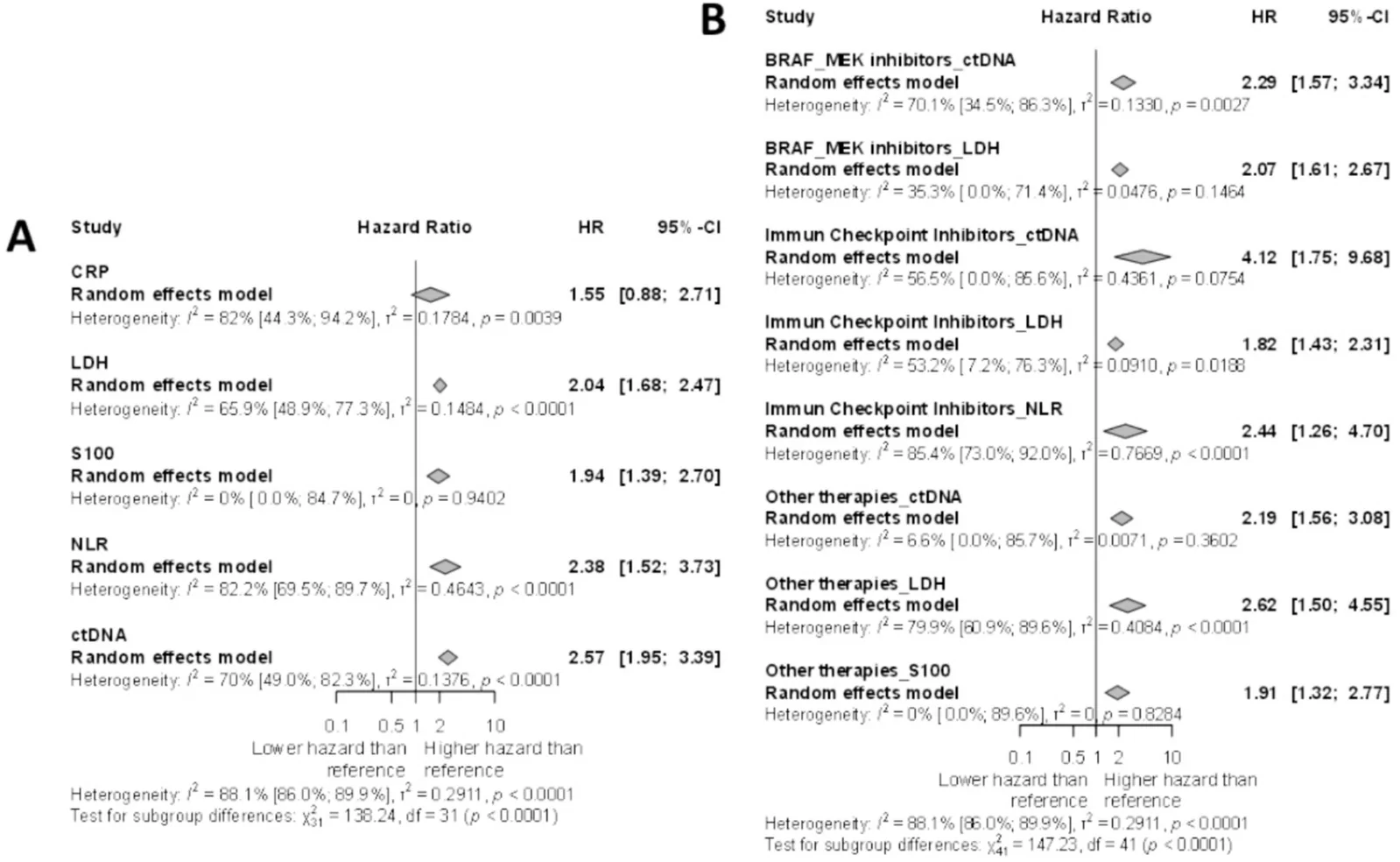
(**A**) Forest plot of univariate Hazard Ratios (HR) of PFS and baseline serum biomarker level associations independently from therapeutic agent. (**B**) Forest plot of univariate Hazard Ratios (HR) of PFS and serum biomarker level associations based on therapeutic agent. CI: confidence interval; CRP: C-reactive protein; ctDNA: circulating tumor DNA; HR: hazard ratio; LDH: lactate dehydrogenase; NLR: neutrophil-to-lymphocyte ratio; S100: S100 calcium-binding protein.

The following analyses focus on the associations between different baseline serum biomarkers and PFS. A total of 39 studies assessed the relationship between the association of PFS and serum biomarker levels. Elevated baseline level of LDH (HR 2.04; 95% CI 1.68–2.47; I^2^ = 65.9%), S100B (HR 1.94; 95% CI 1.39–2.70; I^2^ = 0%), ctDNA (HR 2.57; 95% CI 1.95–3.39; I^2^ = 70%) and NLR (HR 2.38; 95% CI 1.52–3.73; I^2^ = 82.2%) were associated with reduced PFS. In contrast, elevated CRP levels were not associated with PFS (HR 1.55; 95% CI 0.88–2.71; I^2^ = 82%) (see Fig. 4A).

The subgroup analyses stratified by therapeutic agents showed that BRAFMEKi-treated patients with elevated baseline levels of LDH (HR 2.07; 95% CI 1.61–2.67; I^2^ = 35.3%) and ctDNA (HR 2.29; 95% CI 1.57–3.34; I^2^ = 70.1%) were associated with reduced PFS. ICI-treated patients with elevated baseline levels of LDH (HR 1.82; 95% CI 1.43–2.21; I^2^ = 53.2%), ctDNA (HR 4.12; 95% CI 1.75–9.68; I^2^ = 56.5%), and NLR (HR 2.44; 95% CI 1.26–4.70; I^2^ = 85.4%) were associated with reduced PFS. In the OT group, elevated LDH levels (HR 1.08; 95% CI 1.01–3.24; I^2^ = 6.61%) and detectable ctDNA levels (HR 2.19; 95% CI 1.56–3.08; I^2^ = 6.6%) were associated with reduced PFS (see Fig. 4B). Pairwise analysis of biomarkers showed no statistically significant differences between their prognostic associations (see Supplementary Table S8-S9). Stage- and therapy-specific subgroup analyses are presented in the Supplementary Material.

Throughout the treatment follow-up period, detectable ctDNA levels were associated with reduced PFS (HR 4.61; 95% CI 2.89–7.36) (see Supplementary Figure S31). The detailed forest plots for baseline and follow-up results related to the biomarkers are presented in Supplementary Figures S22-S31.

##### Multivariable-adjusted analyses

Multivariable-adjusted hazard ratios were available from 35 studies for LDH, 6 for ctDNA, 5 for CRP, and 12 for NLR. Elevated LDH levels remained significantly associated with worse PFS in a random-effects meta-analytic model (HR 1.71, 95% CI 1.43–2.05; I^2^ = 46.6%) (see Fig. 3B and Supplementary Figure S32).

Elevated ctDNA (HR 1.91, 95% CI 1.07–3.41; I^2^ = 86.4%), NLR (HR 2.26, 95% CI 1.49–4.42; I^2^ = 61.2%) (see Fig. 4B) and CRP (HR 1.81, 95% CI 1.24–2.64; I^2^ = 83.8%) levels were significantly associated with worse PFS in a pooled analysis of HRs (Supplementary Figure S33-S35).

#### Disease-specific survival (DSS)

A total of 7 studies assessed the association between DSS and baseline biomarker levels. Elevated baseline levels of LDH (HR 2.66; 95% CI 2.00–3.55; I^2^ = 0%), S100B (HR 2.11; 95% CI 1.29–3.46; I^2^ = 31.8), and ctDNA (HR 1.96; 95% CI 1.48–2.59; I^2^ = 0%) were associated with reduced DSS (see Supplementary Fig. 37). No difference was observed between the prognostic associations of the evaluated biomarkers for DSS (see Supplemtary Table S11).

Elevated LDH (HR 2.06; 95% CI 1.78–2.39; I^2^ = 0%) and S100B (HR 1,68; 95% CI 1.32–2.15; I^2^ = 0%) levels were significantly associated with worse DSS in a pooled analysis of multivariate HRs (see Fig. 3C and Supplementary Figure S37).

### Qualitative analysis

Across 90 articles, more than 70 biomarkers were evaluated for their association with patient outcomes, summarized in Supplementary Table S2.

#### Risk of bias assessment

The majority of studies included had a moderate to high risk of overall bias, which can be attributed to the retrospective design of the studies. Detailed results of the risk of bias assessments are provided in Supplementary Figure S42 and Supplementary Table S12. Visual inspection of funnel plots did not indicate substantial publication bias; however, in certain subgroup analyses should be interpreted with caution due to the limited number of included studies and potential small-study effects. (see Supplementary Figure S43-S69).

## Discussion

Based on our analysis, LDH, ctDNA, IL-6, NLR, and S100B emerged as the most robust and consistent serum biomarkers associated with reduced OS in melanoma patients. These associations remained significant in multivariable analyses, supporting their role as independent prognostic markers. Across other survival endpoints, LDH, ctDNA, and NLR showed the most consistent associations with reduced PFS, while LDH and S100B showed associations with DSS.

Across the evaluated biomarkers, LDH showed comparable prognostic value to other markers, supporting its continued clinical relevance. These associations remained consistent in multivariable analyses, with a moderate reduction in between-study heterogeneity, reinforcing the role of LDH. As serum LDH is a widely available and cost-effective measurement, monitoring its level should be further recommended.

Despite its widespread clinical use and cost-effectiveness, LDH has important limitations. Its prognostic value appears to be highly dependent on the applied cut-off, complicating clinical interpretation^194^. Our analysis further indicates that the cut-off value significantly moderated effect estimates, indicating that variability in threshold selection contributes to between-study heterogeneity and affects the apparent prognostic strength of LDH.

LDH is a nonspecific marker of tissue damage, which may limit its utility for disease monitoring during immune-based therapies^195^. Beyond reflecting tumor burden and cell turnover, elevated LDH may also indicate biologically active mechanisms that influence antitumor immunity. Rapidly proliferating melanoma lesions often adopt an LDH-mediated glycolytic phenotype, leading to lactate accumulation and microenvironmental acidification that can impair lymphocyte function. Accordingly, elevated LDH reflects both advanced disease and a metabolically and immunologically unfavorable tumor microenvironment.^110,195^ LDH has also been associated with immune-related adverse events in melanoma patients treated with ICIs, potentially reflecting an association with treatment response^196^.

This highlights the need for melanoma-specific biomarkers and supports the use of combinational approaches that integrate LDH with immune-related markers to improve prognostic assessment^197^.

ctDNA showed similar prognostic associations to LDH, with no statistically significant differences observed between their effect sizes. Although ctDNA is a promising tumor-specific biomarker, it does not provide a clear prognostic advantage over LDH, which remains widely used in clinical practice. ctDNA is a tumor-specific biomarker reflecting DNA fragments released into the bloodstream; in melanoma, its relevance is supported by the high mutational burden and frequent driver mutations^11^. Despite their promising prognostic associations, the clinical utility of novel serum biomarkers remains inconclusive due to substantial between-study heterogeneity, lack of standardized cut-off values, and variability in measurement methods. In addition, many biomarkers are supported by a limited number of studies and relatively small sample sizes, and lack consistent validation in prospective settings. Methodological differences (e.g., detectability versus quantitative thresholds for ctDNA) and cost and accessibility limitations, further limit clinical implementation. Nevertheless, ctDNA is not yet routinely available in clinical practice, and its higher cost raises concerns about its practical clinical utility.

Therefore, further investigations are needed to elucidate the predictive power of ctDNA. Although no statistically detectable differences in survival prognostic associations were observed, dynamic changes in early ctDNA levels during therapy were also predictive of therapy response, and increased ctDNA levels correlated with radiological disease progression^94,151^. Elevated S100B level is a well-established prognostic biomarker in metastatic melanoma patient outcomes; however, it is not specific for malignant melanoma as liver and renal injury, inflammatory conditions or infectious disease can influence its level^198^. Consistent with previous literature^78^, our findings indicate that elevated S100B levels were associated with reduced OS. In subgroup analyses, elevated S100B levels in the ICI group trended toward worsened OS; however, the association was not significant. These results raise concerns about using S100B in ICI-treated patients. Our results remained significantly associated in multivariable analysis^94^

In patients receiving ICIs, elevated NLR was consistently associated with worse survival outcomes in both univariable and multivariable analyses. As a marker of systemic inflammation and immune status, NLR may reflect an immunosuppressive tumor microenvironment, where neutrophil-mediated mechanisms can inhibit T-cell activity and reduce the efficacy of immunotherapy^86,199^. Dynamic changes in NLR during treatment have been associated with survival outcomes, with increasing NLR linked to worse prognosis^200^.

Both high baseline NLR and increases during ICI therapy have been linked to poor survival outcomes, possibly reflecting metabolic and immunosuppressive pathways associated with LDH and CD39^97^. Consistent with previous literature, our results showed that elevated NLR has been associated with worse OS, PFS, and DSS in ICI-treated melanoma patients^194^. Given its accessibility and cost-effectiveness, NLR may serve as a useful complementary biomarker alongside LDH in clinical practice.

Based on our results, elevated IL-6 levels were associated with reduced OS, especially in ICI-treatment. This association remained significant in the multivariable model, and the cut-off value itself proved to be a significant moderator of effect estimates. IL-6 is a pleiotropic inflammatory cytokine involved in melanoma progression through JAK/STAT3 signaling and elevated serum levels are consistently associated with poor clinical outcomes^201,202^. Similarly, in advanced cutaneous squamous cell carcinoma patients treated with cemiplimab, elevated baseline serum IL-6 levels and increases during treatment have been associated with shorter PFS^203^. Given the lack of standardized cut-off values across studies, the clinical utility of IL-6 requires further investigation. Other inflammatory biomarkers have been linked to poor outcomes, such as IL-8^163,184^; however, IL-4 and IL-10 showed no significant associations with patient survival^124,143^.

In the qualitative analysis, more than 70 serum biomarkers were reported, among these, two showed promising prognostic potential: CTCs and Melanoma Inhibitory Activity (MIA) protein. Both biomarkers require further investigation; however, their limited accessibility raises questions about their clinical applicability.

To our knowledge, this is the first systematic review and meta-analysis investigating the association between various serum biomarkers and survival outcomes in melanoma patients, while accounting for different therapeutic settings. A key strength of this meta-analysis is its adherence to a pre-registered protocol and Cochrane methodological standards, ensuring transparency, high-quality, and reproducibility. To strengthen our findings, we performed multivariable and meta-regression analyses incorporating ethnicity (based on WHO regions), disease stage, cut-off values, and treatment modality into the model, which demonstrated that elevated LDH, NLR, IL-6, S100B, and ctDNA levels remained independently associated with worsened survival, supporting their role as true independent markers.

The main limitation of this study is the moderate to high heterogeneity among the included studies, which can be attributed to the diversity of study designs and clinical settings. To address this limitation, we conducted additional analyses stratified by treatment modality, disease stage and follow-up duration to identify potential sources of heterogeneity. In some cases, disease stage was inconsistently reported, which limited our ability to perform subgroup analyses by disease stage across all studies. Most analyses were conducted based on baseline levels of the biomarkers; only a few were suitable for follow-up assessment. In some cases, the biomarkers’ cut-off values were not reported consistently, thus influencing the data quality; in particular, ctDNA results are limited by the lack of standardized quantitative cut-off values, with most studies relying on detectability as a binary measure, which may contribute to between-study heterogeneity, thereby influencing our results. An additional limitation of this study was that only indirect comparisons and pairwise analyses could be performed to identify differences between the biomarkers.

Our findings confirm that LDH remains a reliable and easily accessible measurement and should be recommended in routine melanoma patient care. However, emerging serum biomarkers also demonstrate significant prognostic potential and may offer valuable complementary tools for disease monitoring and patient stratification. These findings highlight the importance of translating well-established scientific evidence into standardized clinical practice^204,205^ ctDNA appears promising; however, pairwise analyses did not identify statistically detectable differences compared to LDH. Furthermore, ctDNA measurements are associated with high costs and significant time requirements. Monitoring non-tumor-specific inflammatory markers like NLR in ICI-treated melanoma patients could provide valuable information regarding life expentancy, and their complementary use alongside LDH should be considered for monitoring disease progression and assessing their potential role in future clinical decision-making.

The clinical implementation of novel biomarkers remains challenging; although model-based approaches integrating these markers show promising results, their translation into clinical practice is limited by heterogeneity in model construction, lack of standardized predictors, and insufficient external validation^206^.

This meta-analysis highlights the importance of using unified cut-off values and standardized methods to provide valuable data for investigating biomarkers. Prospective, well-designed studies with standardized protocols are essential to validate biomarkers and establish their role in clinical practice. Future research should prioritize the development of consensus-based protocols, including uniform biomarker definitions and pre-specified thresholds and methodologies. Investigation of biomarker performance stratified by disease stage or treatment modality should be considered. Moreover, the potential of combinatorial biomarker panels should be further explored. The integration of new serum biomarkers into melanoma management has the potential to improve risk stratification, monitor therapeutic efficacy, and inform treatment adaptation, preparing the way towards personalized oncological care. Gene expression profiling represents a promising emerging approach for risk stratification; however, its routine clinical use is not currently recommended by guidelines due to limited evidence supporting its clinical relevance beyond established clinicopathological factors^207^.

LDH remains a reliable, cost-effective, and widely available biomarker. While ctDNA demonstrates potential as a prognostic marker, our analyses did not identify a statistically detectable advantage over LDH, underscoring the need for further studies to clarify its clinical applicability. In ICI-treated patients, NLR may provide complementary predictive information alongside LDH.

## Supplementary Information

Supplementary Information.

## Data Availability

The datasets analysed in this study are available in the published articles included in the systematic review and meta-analysis. Additional data may be obtained from the corresponding author upon reasonable request.
