# Supplementary material for: Predictive value of serum biomarkers for survival in melanoma: a systematic review and meta-analysis
Source: Sci Rep. 2026 Jun 8;16:26099. doi: 10.1038/s41598-026-54347-w (PMC13490590; doi:10.1038/s41598-026-54347-w)
Supplement: Supplementary file 1 [file 41598_2026_54347_MOESM1_ESM.docx]

**Supplementary Material**

**Predictive value of serum biomarkers for survival in malignant melanoma: A Systematic Review and Meta-analysis**

**Authors:** Lili Gulyás^1,2^, Fanni Adél Meznerics^1,2^, Bence Szabó^2^, Noémi Nóra Varga ^1,2^, Lajos Vince Kemény ^1,2,5^, Norbert Kiss^1,2^, Tamara Érseki^1,2^, Péter Hegyi^2,3,4^, András Bánvölgyi ^1,2,#^ Kende Kálmán Lőrincz ^1^

**Affiliations:**

1 Department of Dermatology, Venereology and Dermatooncology, Faculty of Medicine, Semmelweis University, Budapest, Hungary

2 Centre for Translational Medicine, Semmelweis University, Budapest, Hungary

3 Institute for Translational Medicine, Medical School, University of Pécs, Pécs, Hungary

4 Institute of Pancreatic Diseases, Semmelweis University, Budapest, Hungary

5. Department of Physiology, Faculty of Medicine, Semmelweis University

\# These authors share last authorship.

**Corresponding author**

Kende Kálmán Lőrincz, MD, PhD

Postal address: H-1085 Budapest, Mária utca 41., Hungary

Tel.: +(36-1) 266‑0465

Fax: +(36-1) 267‑6974

E-mail address: lorincz.kende@semmelweis.hu

[**Search Strategy**	8](#_Toc221122414)

[Preliminary search key for identifying biomarkers	8](#_Toc221122415)

[Search key for the systematic search used in each electronic database	8](#_Toc221122416)

[**Study and Patient Characteristics**	10](#_Toc221122417)

[**Supplementary Table S1**: Baseline characteristics of the included studies and baseline patient characteristics.	10](#_Toc221122418)

[**Systematic review results not eligible for quantitative synthesis**	47](#_Toc221122419)

[**Supplementary Table S2:** Serum biomarker level associations with melanoma patient outcomes	47](#_Toc221122420)

[**Overall Survival: Univariate analyses**	64](#_Toc221122421)

[**Supplementary Figure S1:** Forest plot of univariate Hazard Ratios (HR) of overall survival (OS) and baseline serum biomarker level associations in melanoma patients, regardless of therapeutic agent.	66](#_Toc221122422)

[**Supplementary Table S2:** Pairwise comparisons of prognostic associations between serum biomarkers for OS.	67](#_Toc221122423)

[**Supplementary Figure S2:** Forest plot of univariate Hazard Ratios (HR) of verall survival (OS) and baseline serum biomarker level associations in melanoma patients subgrouped based on therapeutic agent.	72](#_Toc221122424)

[**Supplementary Table S3:** Pairwise comparisons of prognostic associations between serum biomarkers across therapeutic subgroups.	73](#_Toc221122425)

[**Supplementary Figure S3:** Forest plot of univariate Hazard Ratios (HR) of overall survival (OS) and baseline lactate dehydrogenase (LDH) level associations in stage III-IV stage melanoma patients.	76](#_Toc221122426)

[**Supplementary Figure S4:** Forest plot of univariate Hazard Ratios (HR) of overall survival (OS) and baseline serum lactate dehydrogenase (LDH) level associations subgrouped based on therapeutic agent and disease stage in melanoma patients.	77](#_Toc221122427)

[**Supplementary Figure S5:** Forest plot of univariate Hazard Ratios (HR) of overall survival (OS) and baseline circulating tumor DNA (ctDNA) level associations in stage III-IV stage melanoma patients.	78](#_Toc221122428)

[**Supplementary Figure S6**: Forest plot of univariate Hazard Ratios (HR) of overall survival (OS) and baseline serum circulating tumor DNA (ctDNA) level associations subgrouped based on therapeutic agent and disease stage in melanoma patients.	78](#_Toc221122429)

[**Supplementary Figure S7:** Forest plot of univariate Hazard Ratios (HR) of overall survival (OS) and baseline S100 level associations in stage III-IV stage melanoma patients.	79](#_Toc221122430)

[**Supplementary Figure S8:** Forest plot of univariate Hazard Ratios (HR) of overall survival (OS) and baseline serum S100 level associations subgrouped based on therapeutic agent and disease stage in melanoma patients.	79](#_Toc221122431)

[**Supplementary Figure S9:** Forest plot of univariate Hazard Ratios (HR) of overall survival (OS) and baseline neutrophil-to-lymphocyte ratio (NLR) associations in stage III-IV stage melanoma patients.	80](#_Toc221122432)

[**Supplementary Figure S10:** Forest plot of univariate Hazard Ratios (HR) of overall survival (OS) and baseline neutrophil-to-lymphocyte ratio (NLR) associations subgrouped based on therapeutic agent and disease stage in melanoma patients.	80](#_Toc221122433)

[**Supplementary Figure S11:** Forest plot of univariate Hazard Ratios (HR) of overall survival (OS) and baseline interleukin-6 (IL-6) level associations in stage III-IV stage melanoma patients.	81](#_Toc221122434)

[**Supplementary Figure S12:** Forest plot of univariate Hazard Ratios (HR) of overall survival (OS) and baseline serum interleukin-6 (IL-6) level associations subgrouped based on therapeutic agent and disease stage in melanoma patients.	81](#_Toc221122435)

[**Supplementary Figure S13:** Forest plot of univariate Hazard Ratios (HR) of overall survival (OS) and baseline C-reactive protein (CPR) level associations in stage III-IV stage melanoma patients.	82](#_Toc221122436)

[**Supplementary Figure S14:** Forest plot of univariate Hazard Ratios (HR) of overall survival (OS) and baseline serum C-reactive protein (CRP) level associations subgrouped based on therapeutic agent and disease stage in melanoma patients.	82](#_Toc221122437)

[**Supplementary Figure S15:** Forest plot of univariate Hazard Ratios (HR) of overall survival (OS) and serum biomarker levels association in melanoma patients during follow-up.	83](#_Toc221122438)

[**Overall Survival: Mutlivariable-Analyses**	84](#_Toc221122439)

[**Supplementary Figure S16:** Forest plot of multivariable-adjusted HRs for OS associated with baseline serum LDH in melanoma patients.	84](#_Toc221122440)

[**Supplementary Table S4:** Multivariable random-effects meta-regression model for the association between baseline LDH levels and OS.	84](#_Toc221122441)

[**Supplementary Figure S17:** Forest plot of multivariable-adjusted HRs for OS associated with baseline serum IL-6 in melanoma patients.	85](#_Toc221122442)

[**Supplementary Table S5:** Multivariable random-effects meta-regression model for the association between baseline IL-6 levels and OS.	85](#_Toc221122443)

[**Supplementary Figure S18:** Forest plot of multivariable-adjusted HRs for OS associated with baseline serum NLR in melanoma patients.	85](#_Toc221122444)

[**Supplementary Table S6:** Multivariable random-effects meta-regression model for the association between baseline NLR levels and OS.	85](#_Toc221122445)

[**Supplementary Figure S19:** Forest plot of multivariable-adjusted HRs for OS associated with baseline ctDNA levels in melanoma patients.	86](#_Toc221122446)

[**Supplementary Figure S20:** Forest plot of multivariable-adjusted HRs for OS associated with baseline S100B levels in melanoma patients.	86](#_Toc221122447)

[**Supplementary Figure S21:** Forest plot of multivariable-adjusted HRs for OS associated with baseline CRP levels in melanoma patients.	87](#_Toc221122448)

[**Supplementary Table S7:** Multivariable random-effects meta-regression model for the association between baseline CRP levels and OS.	87](#_Toc221122449)

[**Progression-Free Survival: Univariate analyses**	88](#_Toc221122450)

[**Supplementary Figure S22:** Forest plot of univariate Hazard Ratios (HR) of progression-free survival (PFS) and baseline serum biomarker level associations in melanoma patients, regardless of therapeutic agent.	89](#_Toc221122451)

[**Supplementary Table S8:** Pairwise comparisons of prognostic associations between serum biomarkers and PFS regardless of therapeutic agent.	90](#_Toc221122452)

[**Supplementary Figure S23:** Forest plot of univariate Hazard Ratios (HR) of progression free survival (PFS) and baseline serum biomarker level associations in melanoma patients subgrouped based on therapeutic agent.	92](#_Toc221122453)

[**Supplementary Table S9:** Pairwise comparisons of prognostic associations between serum biomarkers and PFS across therapeutic subgroups.	92](#_Toc221122454)

[**Supplementary Figure S24:** Forest plot of univariate Hazard Ratios (HR) of progression-free survival (PFS) and baseline lactate dehydrogenase (LDH) level associations in stage III-IV stage melanoma patients.	94](#_Toc221122455)

[**Supplementary Figure S25:** Forest plot of univariate Hazard Ratios (HR) of progression-free survival (PFS) and baseline serum lactate dehydrogenase (LDH) level associations subgrouped based on therapeutic agent and disease stage in melanoma patients.	95](#_Toc221122456)

[**Supplementary Figure S26:** Forest plot of univariate Hazard Ratios (HR) of progression-free survival (PFS) and baseline circulating tumor DNA (ctDNA) level associations in stage III-IV stage melanoma patients.	96](#_Toc221122457)

[**Supplementary Figure S27:** Forest plot of univariate Hazard Ratios (HR) of progression-free survival (PFS) and baseline serum circulating tumor DNA (ctDNA) level associations subgrouped based on therapeutic agent and disease stage in melanoma patients.	96](#_Toc221122458)

[**Supplementary Figure S28:** Forest plot of univariate Hazard Ratios (HR) of progression-free survival (PFS) and baseline serum circulating tumor DNA (ctDNA) level associations in different therapeutic subgroups in melanoma patients.	97](#_Toc221122459)

[**Supplementary Figure S29:** Forest plot of univariate Hazard Ratios (HR) of progression-free survival (PFS) and baseline neutrophil-to-lymphocyte ratio (NLR) associations in stage III-IV stage melanoma patients.	97](#_Toc221122460)

[**Supplementary Figure S30:** Forest plot of univariate Hazard Ratios (HR) of progression-free survival (PFS) and baseline neutrophil-to-lymphocyte ratio (NLR) associations subgrouped based on therapeutic agent and disease stage in melanoma patients.	98](#_Toc221122461)

[**Supplementary Figure S31:** Forest plot of univariate Hazard Ratios (HR) of progression-free survival (PFS) and serum biomarker levels association in melanoma patients during follow-up.	98](#_Toc221122462)

[**Progression-Free-Survival: Multivariable analyses**	99](#_Toc221122463)

[**Supplementary Figure S32:** Forest plot of multivariable-adjusted HRs for PFS associated with baseline LDH levels in melanoma.	99](#_Toc221122464)

[**Supplementary Table S10:** Multivariable random-effects meta-regression model for the association between baseline LDH levels and PFS.	99](#_Toc221122465)

[**Supplementary Figure S33:** Forest plot of multivariable-adjusted HRs for PFS associated with baseline ctDNA levels in melanoma.	100](#_Toc221122466)

[**Supplementary Figure S34:** Forest plot of multivariable-adjusted HRs for PFS associated with baseline NLR levels in melanoma.	100](#_Toc221122467)

[**Supplementary Figure S35:** Forest plot of multivariable-adjusted HRs for PFS associated with baseline CRP levels in melanoma.	100](#_Toc221122468)

[**Distant metastasis disease-free- (DM-DFS), disease-free-(DSS) and recurrance-free (RFS) survival: univariate analyses**	101](#_Toc221122469)

[**Supplementary Figure S36:** Forest plot of univariate Hazard Ratios (HR) of distant metastasis disease-free survival (DM-DFS) and serum biomarker levels association in melanoma patients during follow-up.	101](#_Toc221122470)

[**Supplementary Figure S37:** Forest plot of univariate Hazard Ratios (HR) of disease-specific survival (DSS) and baseline serum biomarker level associations in melanoma patients, regardless of therapeutic agent.	101](#_Toc221122471)

[**Supplementary Table S11:**Pairwise comparisons of prognostic associations between serum biomarkers for DSS.	102](#_Toc221122472)

[**Supplementary Figure S38:** Forest plot of univariate Hazard Ratios (HR) of recurrence-free survival (RFS) and baseline serum biomarker level associations in melanoma patients, regardless of therapeutic agent.	102](#_Toc221122473)

[**Distant metastasis disease-free survival and disease-free survival: multivariable analyses**	103](#_Toc221122474)

[**Supplementary Figure S39:** Forest plot of multivariable-adjusted HRs for DM-DFS associated with baseline S100B levels in melanoma patients.	103](#_Toc221122475)

[**Supplementary Figure S40:** Forest plot of multivariable-adjusted HRs for DSS associated with baseline LDH levels in melanoma patients.	103](#_Toc221122476)

[**Supplementary Figure S41:** Forest plot of multivariable-adjusted HRs for DSS associated with baseline S100B levels in melanoma.	103](#_Toc221122477)

[**Risk of Bias assessment**	104](#_Toc221122478)

[**Supplementary Table S12:** Risk of bias and concerns regarding applicability assessment using the QUIPS (Quality In Prognosis Studies) tool.	104](#_Toc221122479)

[**Supplementary Figure S42**: Proportion of studies with low, high, or unclear risk of bias.	114](#_Toc221122480)

[**Publication Bias**	115](#_Toc221122481)

[**Supplementary Figure S43:** Funnel plot for studies of overall survival (OS) and baseline serum biomarker level associations in melanoma patients, independently from therapeutic agent.	115](#_Toc221122482)

[**Supplementary Figure S44:** Funnel plot for studies assessing overall survival (OS) and baseline serum biomarker level associations in melanoma patients based on therapeutic agent.	116](#_Toc221122483)

[**Supplementary Figure S45:** Funnel plot for studies assessing overall survival (OS) and baseline lactate dehydrogenase (LDH) level associations in stage III-IV stage melanoma patients.	117](#_Toc221122484)

[**Supplementary Figure S46:** Funnel plot for studies assessing overall survival (OS) and baseline serum lactate dehydrogenase (LDH) level associations subgrouped based on therapeutic agent and disease stage in melanoma patients.	118](#_Toc221122485)

[**Supplementary Figure S47:** Funnel plot for studies assessing overall survival (OS) and baseline circulating tumor DNA (ctDNA) level associations in stage III-IV stage melanoma patients.	119](#_Toc221122486)

[**Supplementary Figure S48:** Funnel plot for studies assessing overall survival (OS) and baseline serum circulating tumor DNA (ctDNA) level associations subgrouped based on therapeutic agent and disease stage in melanoma patients.	120](#_Toc221122487)

[**Supplementary Figure S49:** Funnel plot for studies assessing overall survival (OS) and baseline S100 level associations in stage III-IV stage melanoma patients.	121](#_Toc221122488)

[**Supplementary Figure S50:** Funnel plot for studies assessing overall survival (OS) and baseline serum S100 level associations subgrouped based on therapeutic agent and disease stage in melanoma patients.	122](#_Toc221122489)

[**Supplementary Figure S51:** Funnel plot for studies assessing overall survival (OS) and baseline neutrophil-to-lymphocyte ratio (NLR) associations in stage III-IV stage melanoma patients.	123](#_Toc221122490)

[**Supplementary Figure S52:** Funnel plot for studies assessing overall survival (OS) and baseline neutrophil-to-lymphocyte ratio (NLR) associations subgrouped based on therapeutic agent and disease stage in melanoma patients.	124](#_Toc221122491)

[**Supplementary Figure S53:** Funnel plot for studies assessing overall survival (OS) and baseline interleukin-6 (IL-6) level associations in stage III-IV stage melanoma patients.	125](#_Toc221122492)

[**Supplementary Figure S54:** Funnel plot for studies assessing overall survival (OS) and baseline serum interleukin-6 (IL-6) level associations subgrouped based on therapeutic agent and disease stage in melanoma patients.	126](#_Toc221122493)

[**Supplementary Figure S55:** Funnel plot for studies assessing overall survival (OS) and baseline C-reactive protein (CRP) level associations in stage III-IV stage melanoma patients.	127](#_Toc221122494)

[**Supplementary Figure S56:** Funnel plot for studies assessing overall survival (OS) and baseline serum C-reactive protein (CRP) level associations subgrouped based on therapeutic agent and disease stage in melanoma patients.	128](#_Toc221122495)

[**Supplementary Figure S57:** Funnel plot for studies assessing overall survival (OS) and serum biomarker levels association in melanoma patients during follow-up.	129](#_Toc221122496)

[**Supplementary Figure S58:** Funnel plot for studies assessing progression-free (PFS) and baseline serum biomarker level associations in melanoma patients, regardless of therapeutic agent.	130](#_Toc221122497)

[**Supplementary Figure S59:** Funnel plot for studies assessing progression-free survival (PFS) and baseline serum biomarker level associations in melanoma patients subgrouped based on therapeutic agent.	131](#_Toc221122498)

[**Supplementary Figure S60:** Funnel plot for studies assessing progression-free survival (PFS) and baseline lactate dehydrogenase (LDH) level associations in stage III-IV stage melanoma patients.	132](#_Toc221122499)

[**Supplementary Figure S61**: Funnel plot for studies assessing progression-free survival (PFS) and baseline serum lactate dehydrogenase (LDH) level associations, subgrouped based on therapeutic agent and disease stage in melanoma patients.	133](#_Toc221122500)

[**Supplementary Figure S62:** Funnel plot for studies assessing progression-free survival (PFS) and baseline circulating tumor DNA (ctDNA) level associations in stage III-IV stage melanoma patients.	134](#_Toc221122501)

[**Supplementary Figure S63:** Funnel plot for studies assessing progression-free survival (PFS) and baseline serum circulating tumor DNA (ctDNA) level associations subgrouped based on therapeutic agent and disease stage in melanoma patients.	135](#_Toc221122502)

[**Supplementary Figure S64**: Funnel plot for studies assessing progression-free survival (PFS) and baseline neutrophil-to-lymphocyte ratio (NLR) associations in stage III-IV stage melanoma patients.	136](#_Toc221122503)

[**Supplementary Figure S65:** Funnel plot for studies assessing progression-free survival (PFS) and baseline neutrophil-to-lymphocyte ratio (NLR) associations subgrouped based on therapeutic agent and disease stage in melanoma patients.	137](#_Toc221122504)

[**Supplementary Figure S66:** Funnel plot for studies assessing progression-free survival (PFS) and serum biomarker levels association in melanoma patients during follow-up.	138](#_Toc221122505)

[**Supplementary Figure S67:** Funnel plot for studies assessing distant metastasis disease-free survival (DM-DFS) and serum biomarker levels association in melanoma patients during follow-up.	139](#_Toc221122506)

[**Supplementary Figure S68:** Funnel plot for studies assessing disease-specific survival (DSS) and baseline serum biomarker level associations in melanoma patients, regardless of therapeutic agent.	140](#_Toc221122507)

[**Supplementary Figure S69:** Funnel plot for studies assessing recurrence-free survival (RFS) and baseline serum biomarker level associations in melanoma patients, regardless of therapeutic agent.	141](#_Toc221122508)

[**Multivariate-model diagnostics**	142](#_Toc221122509)

[**Supplementary Figure S70:** Fitted versus observed values from the multivariate meta-analysis model assessing baseline LDH levels and OS,demonstrating adequate model fit.	142](#_Toc221122510)

[**Supplementary Figure S71:** Funnel plot of the multivariate meta-analysis model for baseline LDH and OS.	143](#_Toc221122511)

[**Supplementary Figure S72:** Normal Q–Q plot of standardized residuals for the multivariate meta-analysis of baseline LDH and OS, indicating no major deviation from normality.	144](#_Toc221122512)

[**Supplementary Figure S73:** Study-level effect estimates from the multivariate random-effects meta-analysis model for baseline LDH and OS.	145](#_Toc221122513)

[**Supplementary Figure S74:** Fitted versus observed values from the multivariate meta-analysis model assessing baseline IL-6 levels and OS, demonstrating adequate model fit.	146](#_Toc221122514)

[**Supplementary Figure S75:** Funnel plot of the multivariate meta-analysis model for baseline IL-6 and OS.	147](#_Toc221122515)

[**Supplementary Figure S76:** Normal Q–Q plot of standardized residuals for the multivariate meta-analysis of baseline IL-6 and OS, indicating no major deviation from normality.	148](#_Toc221122516)

[**Supplementary Figure S77:** Study-level effect estimates from the multivariate random-effects meta-analysis model for baseline IL-6 and OS.	149](#_Toc221122517)

[**Supplementary Figure S78:** Funnel plot of the multivariate meta-analysis model for baseline NLR and OS.	150](#_Toc221122518)

[**Supplementary Figure S79:** Normal Q–Q plot of standardized residuals for the multivariate meta-analysis of baseline NLR and OS, indicating no major deviation from normality.	151](#_Toc221122519)

[**Supplementary Figure S80:** Study-level effect estimates from the multivariate random-effects meta-analysis model for baseline NLR and OS.	152](#_Toc221122520)

[**Supplementary Figure S81:** Funnel plot of the multivariate meta-analysis model for baseline ctDNA and OS.	153](#_Toc221122521)

[**Supplementary Figure S82:** Funnel plot of the multivariate meta-analysis model for baseline S100B and OS.	154](#_Toc221122522)

[**Supplementary Figure S83:** Fitted versus observed values from the multivariate meta-analysis model assessing baseline CRP levels and OS, demonstrating adequate model fit.	155](#_Toc221122523)

[**Supplementary Figure S84:** Funnel plot of the multivariate meta-analysis model for baseline CRP and OS.	156](#_Toc221122524)

[**Supplementary Figure S85:** Normal Q–Q plot of standardized residuals for the multivariate meta-analysis of baseline CRP and OS, indicating no major deviation from normality.	157](#_Toc221122525)

[**Supplementary Figure S86:** Study-level effect estimates from the multivariate random-effects meta-analysis model for baseline CRP and OS.	158](#_Toc221122526)

[**Supplementary Figure S87:** Funnel plot of the multivariate meta-analysis model for baseline LDH and PFS.	159](#_Toc221122527)

[**Supplementary Figure S88:** Normal Q–Q plot of standardized residuals for the multivariate meta-analysis of baseline LDH and PFS, indicating no major deviation from normality.	160](#_Toc221122528)

[**Supplementary Figure S89:** Funnel plot of the multivariate meta-analysis model for baseline ctDNA and PFS.	161](#_Toc221122529)

[**Supplementary Figure S90:** Funnel plot of the multivariate meta-analysis model for baseline NLR and PFS.	162](#_Toc221122530)

[**Supplementary Figure S91:** Funnel plot of the multivariate meta-analysis model for baseline CRP and PFS.	163](#_Toc221122531)

[**Supplementary Figure S92:** Funnel plot of the multivariate meta-analysis model for baseline LDH and DSS.	164](#_Toc221122532)

[**Supplementary Figure S93:** Funnel plot of the multivariate meta-analysis model for baseline S100B and DSS.	165](#_Toc221122533)

[**SupplementaryReferences**	166](#_Toc221122534)

# **Search Strategy**

## Preliminary search key for identifying biomarkers

melanom* AND (biomark* OR marker*) AND (surviv* OR prognos* OR predict* OR respons* OR relaps* OR recur*)

## Search key for the systematic search used in each electronic database

MEDLINE (via PubMed):

melanom* AND („LDH” OR „Lactate dehydrogenase" OR miRNA* OR microRNA* OR miR* OR ((ct OR cf OR „circulating tumor” OR „cell free”) AND („DNA” OR „deoxyribonucleic acid” OR cell* OR „nucleic acid”)) OR S100* OR „S 100*” OR „osteopontin” OR „OPN” OR „sialoprotein I” OR „early T lymphocyte activation” OR „ETA 1” OR „secreted phosphoprotein 1” OR „SPP1” OR „YKL 40” OR „Chitinase 3 Like" OR „CHI3L1” OR "melanoma inhibitory activity protein" OR „MIA” OR "growth and differentiation factor 15" OR „GDF 15” OR cysteinyldopa* OR „5 s cd” OR CEACAM1 OR „carcinoembryonic antigen related cell adhesion molecule 1” OR „biliary glycoprotein I” OR „CD66a7” OR „vascular endothelial growth factor" OR „VEGF” OR „vascular permeability factor” OR „VPF” OR „TA90IC” OR „TA90 immune complex ” OR "Cytoplasmic melanoma-associated antigen" OR „CYT MAA” OR „C reactive protein" OR „CRP” OR "hepatocyte growth factor" OR „HGF” OR "monocyte chemotactic protein 2" OR „MCP 2” OR "interleukin 6" OR „IL 6” OR "interleukin 8" OR „IL 8” OR „CXCL5” OR „C X C motif chemokine 5” OR „ENA78” OR „VCAM 1” OR „CD106” OR „Vascular cell adhesion molecule 1” OR „sPDL 1” OR „serum PDL 1”) AND (surviv* OR prognos* OR predict* OR respons* OR recurr* OR relaps*)

No specific filters or settings were used

Embase:

melanoma AND (LDH OR „Lactate dehydrogenase” OR miRNA* OR microRNA* OR miR* OR ((ct OR cf OR „circulating tumor” OR „cell free”) AND (DNA OR „deoxyribonucleic acid” OR cell* OR „nucleic acid”)) OR S100* OR „S 100*” OR „osteopontin” OR „OPN” OR „sialoprotein I” OR „early T lymphocyte activation” OR „ETA 1” OR „secreted phosphoprotein 1” OR „SPP1” OR „YKL 40” OR „Chitinase 3 Like” OR CHI3L1 OR „melanoma inhibitory activity protein” OR MIA OR „growth and differentiation factor 15” OR (GDF 15) OR cysteinyldopa* OR „5 s cd” OR CEACAM1 OR „carcinoembryonic antigen related cell adhesion molecule 1” OR „biliary glycoprotein I” OR CD66a7 OR „vascular endothelial growth factor” OR VEGF OR „vascular permeability factor” OR VPF OR TA90IC OR „TA90 immune complex” OR „cytoplasmic melanoma associated antigen” OR „CYT MAA” OR „C reactive protein” OR CRP OR „hepatocyte growth factor” OR HGF OR „monocyte chemotactic protein 2” OR „MCP 2” OR „interleukin 6” OR „IL 6” OR „interleukin 8” OR „IL 8” OR CXCL5 OR „C X C motif chemokine 5” OR ENA78 OR „VCAM 1” OR CD106 OR „Vascular cell adhesion molecule 1” OR „s PDL 1” OR „serum PDL 1”) AND (surviv* OR prognos* OR predict* OR respons* OR recurr* OR relaps*)

The search was conducted in titles, abstracts, and keywords. Advanced search was used, with every setting in mapping unchecked. No special settings, filters, or restrictions were used.

Cochrane Library (CENTRAL):

melanom* AND (LDH OR Lactate dehydrogenase OR miRNA* OR microRNA* OR miR* OR ((ct OR cf OR circulating tumor OR cell free) AND (DNA OR deoxyribonucleic acid OR cell* OR nucleic acid)) OR S100* OR S 100* OR osteopontin OR OPN OR sialoprotein I OR early T lymphocyte activation OR ETA 1 OR secreted phosphoprotein 1 OR SPP1 OR YKL 40 OR Chitinase 3 Like OR CHI3L1 OR melanoma inhibitory activity protein OR MIA OR growth and differentiation factor 15 OR GDF 15 OR sGDF 15 OR cysteinyldopa OR 5 s cd OR CEACAM1 OR carcinoembryonic antigen related cell adhesion molecule 1 OR biliary glycoprotein I OR CD66a7 OR vascular endothelial growth factor OR VEGF OR vascular permeability factor OR VPF OR TA90IC OR TA90immune complex OR Cytoplasmic melanomaassociated antigen OR CYT MAA OR C reactive protein OR CRP OR hepatocyte growth factor OR HGF OR monocyte chemotactic protein 2 OR MCP 2 OR interleukin 6 OR IL 6 OR interleukin 8 OR IL 8 OR CXCL5 OR C X C motif chemokine 5 OR ENA78 OR VCAM 1 OR CD106 OR Vascular cell adhesion molecule 1 OR s PDL 1 OR serum PDL 1) AND (surviv* OR prognos* OR predict* OR respons* OR recurr* OR relaps*)

No special settings, filters, or restrictions were used. Advanced search was used (all fields).

# **Study and Patient Characteristics**

## **Supplementary Table S1**: Baseline characteristics of the included studies and baseline patient characteristics.

| Author, Year | No. patients | Median age in years (range _min_ – range _max_) | Gender (No. females) | Median follow up, m | AJCC Stage | Therapy | Country, period | Study type | Biomarkers (cut-off values) | Investigated outcomes |
| --- | --- | --- | --- | --- | --- | --- | --- | --- | --- | --- |
| Articles included in the quantitative analysis | | | | | | | | | | |
| Immune Checkpoint Inhibitors | | | | | | | | | | |
| Aamdal, 2021 [1] | 151 | 63 (27-84) | 55 (36) | 68.1 m | III-IV | anti-CTLA-4 | Norway, (multicenter), January 2014 - March 2015 | Prospective, open label, single-armed, phase IV interventional clinical trial | CRP (10 mg/l), LDH (ULN), WBC (ULN), ANC (NA), ALC (NA), NLR (5) | OS, PFS |
| Abu-Sbeih, 2019 [2] | 173 | 57.3 NA | NA | NA | III-IV | anti-CTLA-4 or anti-PD-1/L1; anti-CTLA-4 + anti-PD-1/L1 | USA, January 2010 - April 2018 | Retrospective observational study | LDH (618 IU/l), S100B (NA) | OS, PFS |
| Amaral, 2020 [3] | 380 | NA | 140 (36.8) | 18 m | IV | anti-CTLA-4+ anti-PD-1 | Germany (multicenter), April 2015 – 31st October 2018 | Retrospective observational study | LDH (ULN), S100B (ULN) | OS |
| Andressa, 2020 [4] | 140 | NA | NA | NA | III-IV | anti-PD-1; anti-CTLA-4; anti-PD-1+anti-CTLA-4; chemotherapy | Multinational, NA | Retrospective analysis (Checkmate064) | IL-6 (13.3 pg/ml), LDH (ULN), NLR (continuous), CRP (5.3 μg/ml) | OS |
|  | 196 | NA | NA | NA | III-IV | anti-PD-1 | Multinational, NA | Retrospective analysis (Checkmate066) | IL-6 (6 pg/ml), CRP (5.3 μg/ml), |  |
|  | 293 | NA | NA | NA | III-IV | anti-CTLA-4 | Multinational, NA | Retrospective analysis (Checkmate067) | IL-6 (NA) |  |
|  | 290 | NA | NA | NA | III-IV | anti-CTLA-4 | Multinational, NA | Retrospective analysis (Checkmate067) | CRP (5.75 μg/ml) |  |
|  | 287 | NA | NA | NA | III-IV | anti-PD-1 | Multinational, NA | Retrospective analysis (Checkmate067) | IL-6 (11 pg/ml), CRP (5.75 μg/ml) |  |
|  | 290 | NA | NA | NA | III-IV | anti-PD-1+anti-CTLA-4 | Multinational, NA | Retrospective analysis (Checkmate067) | IL-6 (NA), CRP (5.75 μg/ml) |  |
| Arheden, 2020 [5] | 116 | 66 (27-98) | 54 (46.6) | NA | IV | anti-PD-1 or anti-CTLA-4 | Sweden, September 2015 - August 2017 | Retrospective observational study | LDH (ULN) | OS |
| Bhatia, 2019 [6] | 88 | 63.5 NA† | 31 (35.2) | NA | NA | anti-PD-1; anti-CTLA-4; anti-PD-1+ anti-CTLA-4 | USA, 2010 - 2018 | Retrospective observational study | LDH (ULN) | OS |
| Capone, 2018 [7] | 97 | 61 (21-85) | 45 (46.3) | NA | IV | anti-PD-1 | Italy, February 2015 – February 2017 | Retrospective observational study | NLR (5), dNLR (3), ANC (5.4), LDH (450 U/L) | OS, PFS |
| Damuzzo, 2016 [8] | 44 | 63 (33-83) | F15 (65.9) | NA | IV | anti-CTLA-4 | Italy, NA | Prospective observational study | S100B (NA), CRP (ULN), LDH (ULN) | OS |
| Eroglu, 2023 [9] | 30 | 72 (21-90) | 14 (47) | 19.6 m | III | anti-PD-1+ anti- CTLA-4 | USA, April 2020- March 2022 | Retrospective observational study | ctDNA (negative/positive) | PFS |
| Gray, 2015 [10] | 48 | NA | NA | NA | NA | BRAFi ; BRAFi/MEKi ; anti-PD-1; anti-CTLA-4 ; anti-PD-1+ anti-CTLA-4 | Australia, April 2013- February 2015 | Prospective observational study | ctDNA (detectable vs undetectable), LDH | PFS |
| Heidelberger, 2017 [11] | 63 | 65 (22-90) | 30 (47.7) | 7 m | III | anti-PD-1 | France, August 2014- February 2016 | Retrospective observational study | LDH (NA), NLR (NA), CRP (NA) | PFS |
| Herbertreau, 2018 [12] | 53 | 64 (27-90) | 24 (45.3) | 6.8 m | III, IV | anti-PD-1; anti-CTLA-4 + anti-PD-1 | France, NA | Retrospective observational study | ctDNA (detectable vs undetectable), LDH (ULN) | PFS |
| Hoejberg, 2012 [13] | 103 | 53 (29-74) | 43 (42) | NA | IV | immunotherapy (IL-2 ± IFN) | Denmark, February 1993- December 2003 | Phase II study | IL-6 (4.9 ng/l), LDH (ULN) | OS |
| Kelderman, 2014 [14] | 230 | 55 (22-88) | 69 (0.3) | Netherlands cohort: 17.9 m, UK cohort: 19.0 m | IV | anti-CTLA-4 | The Netherlands (multicenter), UK, NA | Retrospective observational real-world study | LDH (ULN) | OS, PFS |
| Khoja, 2016 [15] | 183 | 58 (24-89) | 68 (37.16) | 7.5 m | III-IV | anti-CTLA-4 | Canada, 2008 - 2015 | Retrospective observational study | NLR (4) | OS |
| Koguchi, 2015 [16] | 124 | 57 (23-90) | NA (38.7) | 31 m | NA | anti-CTLA-4 | USA, NA | Retrospective observational study | LDH (NA), ALC (NA) | OS |
| Krkowski, 2021 [17] | 138 | 62 (20-90) | 101 (40.1) | 15 m | IV | BRAFi/MEKi ; BRAFi; anti-PD-1; anti-CTLA-4; anti-CTLA-4 + anti-PD-1 | Sweden, 1^st^ January 2010- 3^rd^ December 2017 | Retrospective observational study | LDH (ULN), albumin (25 g/l), NLR (3) | PFS |
| Lee, 2017 [18] | 86 | NA | 34 (40) | 17.5 m | IV | anti-PD-1; anti-PD-1 + anti-CTLA-4 | Australia, July 2014 - December 2015 | Retrospective observational study | LDH (NA) | OS |
| Lee, 2020 [19] | 59 | NA | 18 (31) | 35.6 m | NA | anti-PD-1 | Australia, April 2014- November 2018 | **Prospective observational study** | ctDNDA | OS |
| Machiraju, 2023 [20] | 303 | 64 (61-66) | 124 (40.92) | NA | IV | anti-PD1 | Germany, May 2013- August 2026 | Retrospective observational study | LDH (ULN) | PFS |
| Mallardo, 2023 [21] | 78 | 61 (27-91) | 41 (52.6) | 51.8 m | III-IV | anti-PD-1 | Italy, 2016 April - 2018 June | Retrospective observational study | NLR (5.57) | OS, PFS |
| Musaelyan, 2022 [22] | 29 | 57 (53-62) | 13 (44.8) | NA | III | anti-PD-1 | Russia, September 2018 - July 2021 | Retrospective observational study | β2-microglobulin (2.5 mg/l), anti-TPO (50 IU/ml), IL-6 (10 pg/ml), IL-18 (NA), NPT (12 nmol/l), NLR (5) | PFS |
| Pedersen, 2020 [23] | 16 | 57 (44-57) | 5 (31) | 26 m | III- IV | anti-CTLA-4 ; anti-PD-1; anti-CTLA-4 + anti-PD-1 | Denmark, October 2016 - August 2017 | Prospective observational study | ctDNA (detectable vs undetectable) TNF-α (NA), MCP-1 (NA), IFN β (NA), cytokine profiles (low vs high) | PFS |
| Rogala, 2022 [24] | 207 | 57 (24-89) | 106 (51.2) | 16.8 m | III-IV | anti-PD-1 | Poland, December 2015 - January 2022 | Retrospective observational study | LDH (ULN) | OS, PFS |
| Rose, 2021 [25] | 230 | NA | 104 (42) | 20.5 m | IIIc-IV | anti-PD-1; anti-PD-1 + anti-CTLA-4 | Canada, 2012 - 2019 | Retrospective observational study | LDH (1,5 x ULN), NLR (5) | OS, PFS |
| Rossi, 2022 [26] | 87 | 62 (53-73) | 30 (34.5) | NA | IV | anti-PD-1; anti-CTLA-4; anti-PD-1 + anti-CTLA-4 | UK, The Netherlands, NA | **Prospective observational study** | IL-6 (2 pg/l), HGF (NA), MCP- (NA) | OS |
| Rosner, 2018 [27] | 209 | 60.5 (22-86.4) | 85 (40.6) | 13.1 m | IV | anti-CTLA-4 + anti-PD-1 | USA, NA | Retrospective observational study | Relative eosinophils (1,1), Absolute monocytes (0,8), Relative basophils (0,6), LDH (246 U/l), NLR (4.73) | OS |
| Schmidt, 2005a [28] | 321 | 51 | 181 (56.3) | 52 m | NA | immunotherapy (IL-2) | Denmarko, April 1990 - December 2003 | Retrospective observational study (clinical trial cohorts) | LDH (NA), Neutrophil counts (NA), Monocyte counts (NA) | OS |
| Schraag, 2019 [29] | 69 | 57 (26-82) | 27 (39.1) | NA | NA | anti-PD-1; anti-CTLA-4 | Germany, July 2006 – July 2016 | Retrospective observational study (registry) | LDH (250 U/l), S100B (0.15 μg/l) | OS |
| Seremet, 2019 [30] | 85 | 57 (28-82) | 37 (43.53) | 19.3 m | III, IV | anti-PD-1 | Belgium, September 2014 - April 2017 | Prospective observational study | LDH (ULN), ctDNA (500 copies/ml), CRP (ULN) | OS, PFS |
| Simentic, 2020 [31] | 38 | 63.4 (11.1) † | 9 (23.7) | NA | NA | anti-PD-1 | Croatia, March 2017 - August 2018 | Prospective observational study | S100B (change) | OS, PFS |
| Svedman, 2022 [32] | 90 | NA | 30 (33.3) | NA | IV | anti-CTLA-4; anti-PD-1  ; anti-CTLA-4 + anti-PD-1 | Sweden, 2012 - 2019 | Retrospective observational study | Thymidine kinase activity (60 Du/l), LDH (ULN) | OS, PFS |
| Trommer, 2022 [33] | 65 | 62.1 NA† | 38 | NA | IV | anti-PD-1 | Germany, September 2014 - November 2020 | Retrospective observational study | LDH (ULN), NLR (3) | OS, PFS |
| Tsutsumida, 2020 [34] | 68 | NA | 29 (42.64) | NA | NA | anti-PD-1+ anti-CTLA-4 | Japan, 31^st^ August, 2015 – 31^st^ March 2017 | Retrospective observational study | CRP (1 mg/l increase), NLR (4), LDH (ULN) | OS, PFS |
| Turiello, 2020 [35] | 453 | 61 (19- 94) | 182 (40.2) | 22 m | III-IV | anti-PD-1 | Multicenter (Italy, Germany, France, Switzerland, USA), NA | Retrospective observational study | LDH (ULN) | OS, PFS |
| Váraljai, 2020 [36] | 35 | NA | 38 (39.58) | NA | III | anti-PD-1 + anti-CTLA-4 | Germany, NA | Prospective observational study | ctDNA (detectable vs undetectable) | OS, PFS |
| Vila, 2021 [37] | 44 | 55 (29-76) | NA | 37.7 m | IV | anti-PD-1; anti-CTLA-4 | Spain, March 2016 – March 2020 | Retrospective observational study | LDH (234 U/l), NLR (5), dNLR (3) | OS |
| Zaragoza, 2016 [38] | 58 | 54.7 NA† | 25 (43) | 30.6 m | III-IV | anti-CTLA-4 | France, April 2008- February 2014 | Retrospective observational study | LDH (ULN), NLR (3) | OS |
| Zhao, 2022 [39] | 165 | NA | 80 (48.5) | NA | IV | anti-PD-1 | China, March 2015 - December 2019 | Retrospective observational study | LDH | OS |
| BRAF/MEK inhibitor therapy | | | | | | | | | | |
| Cocorocchio, 2020 [40] | 90 | 53 (27-81) | 28 (31.1) | 28 m | IV | BRAFi; BRAFi/MEKi | Italy, May 2010 - June 2016 | Retrospective observational study | ANC (7.5x10^6^/ml), ALC (1x10^6^/ml), LDH (ULN), NLR (5), | PFS |
| Czirbesz, 2017 [41] | 43 | 57 NA† | 22 (51.1) | 15.9 m | III-IV | BRAFi | Hungary, November 2012 - October 2015 | Retrospective observational study | LDH (NA) | OS |
| Depenni, 2018 [42] | 76 | 55 (24-86) | 55 (72.37) | 12 m | NA | BRAFi/MEKi | Italy, November 2013 - February 2017 | Retrospective observational real-world study | LDH (NA) | PFS, OS |
| Dutriaux, 2022 [43] | 275 | 57.6 (14.8) † | 109 (39.64) | 5.63 m | IV | BRAFi/MEKi | France, March 2015 -November 2016 | Phase IIIb, open-label, non-randomized access study | LDH (ULN) | PFS |
| Finon, 2018 [44] | 49 | 61.4 (47- 71.6) | 27 (55) | NA | III-IV | BRAFi | France, July 2011 - December 2014 | Retrospective observational study | NLR (4), LDH (NA), Neutrophyl count (NA), Lymphocyte count (NA) | PFS |
| Garlan, 2017 [45] | 11 | 60.2 (38-77) | 5 (45.5) | NA | IV | BRAFi | France, NA | Retrospective observational study (VEMUMELA trial) | ctDNA (detectable; undetectable) | OS |
| Gassenmeier, 2021 [46] | 191 | NA | 85 (44.5) | NA | IV | BRAFi | Germany, August 2010 - December 2016 | Retrospective observational study | LDH (NA), S100B (NA) | DSS, DSSap |
| Gray, 2015 [10] | 48 | NA | NA | NA | NA | BRAFi ; BRAFi/MEKi ; anti-PD-1; anti-CTLA-4 ; anti-PD-1+ anti-CTLA-4 | Australia, April 2013- February 2015 | Prospective observational study | ctDNA (detectable vs undetectable), LDH | PFS |
| Kozak, 2019 [47] | 62 | 50 (25-77) | 30 (48) | 55.4 m | III-IV | BRAFi | Poland, NA | Retrospective observational study | ctDNA (continuous variable) | OS, PFS |
| Krkowski, 2021 [17] | 114 | 62 (20-90) | 101 (40.1) | 15 m | IV | BRAFi/MEKi ; BRAFi | Sweden, 1^st^ January 2010- 3^rd^ December 2017 | Retrospective observational study | LDH (ULN), albumin (25 g/l), NLR (3) | PFS |
| Long, 2016 [48] | 24 | 54.5 (25–74) | 7 (29) | 47.1 m | IIIC -IV | BRAFi/MEKi | International, March 2010 - July 2011 | Open label Phase I-II clinical trial | LDH (ULN) | OS, PFS |
| Long, 2016 [48] | 54 | 57.5 (27–79) | 20 (37) | 47.1 m | IIIC -IV | BRAFi/MEKi | International, March 2010 - July 2011 | Open label Phase I-II clinical trial | LDH (ULN) | OS, PFS |
| Lu, 2018 [49] | 337 | 56 (21–86) | 137 (40.7) | NA | III- IV | BRAFi | USA, NA | Retrospective observational study (BRIM3) | ctDNA BRAFV600 (detectable vs undetectable), cHGF (NA) | OS |
| Ruggiero, 2022 [50] | 70 | 54 (26-89) | 34 (48.6) | NA | III-IV | BRAFi; BRAFi/MEKi | Italy, NA | Retrospective observational study | LDH (ULN), miR-579-3p GM, miR-4488 GM, miR-4443, miR-199b-5p, miR-9-5p, miR-204-5p | OS, PFS |
| S. Ugurel, 2014 [51] | 300 | 56.5 (24.8-78.5) | 136 (45.3) | 13 m | IV | BRAFi | Germany,Austria, NA | Retrospective observational study | LDH (ULN) | OS, PFS |
| Saiag, 2021 [52] | 856 | 58.5 NA† | 474 (55.3) | 5.6 m | III-IV | chemotherapy | France, March 2015 – November 2016 | Open-label, non-randomized, phase IIIb clinical trial | LDH (ULN) | PFS |
| Syeda, 2022 [53] | 345 | 56 (45-65) | 162 (47) | 20 m | III-IV | BRAFi; BRAFi /MEKi | USA, 4^th^ May 2012 – 30^th^ Nov 2012 | **Prospective biomarker analysis (samples:** COMBI-d and COMBI-MB) | ctDNA (detectable vs undetectable), LDH (ULN) | PFS, OS |
| Váraljai, 2020 [36] | 33 | NA | 38 (39.58) | NA | III | target therapy (MAPK) | Germany, NA | Prospective observational study | ctDNA (detectable vs undetectable) | OS, PFS |
| Other therapies | | | | | | | | | | |
| Aamdal, 2021 [1] | 151 | 63 (27-84) | 55 (36) | 68.1 m | III-IV | anti-CTLA-4 | Norway, (multicentre), January 2014 - March 2015 | Prospective, open label, single-armed, phase IV interventional clinical trial | CRP (10 mg/l), LDH (ULN), WBC (ULN), ANC (NA), ALC (NA), NLR (5) | OS, PFS |
| Allen, 2011 [54] | 39 | NA | 17 (43.59) | NA | IV | BRAFi/MEKi alpha-therapy | Australia, 12t^h^ August 2009 | Phase I clinical trial | LDH (225 U/l) | OS |
| Andressa, 2020 [4] | 191 | NA | NA | NA | III-IV | chemotherapy | Multinational, NA | Retrospective analysis (Checkmate066) | IL-6 (6 pg/ml),CRP (5.3 μg/ml), LDH (ULN), NLR (continous) | OS |
| Aoude, 2023 [55] | 106 | 66 (28-90) | 23 (21.7) | 37.13 m | III, IV | BRAFi/MEKi ; immunotherapy | Australia, May 2014 -November 2020 | Prospective observational study | ctDNA (detectable/undetectable), LDH (ULN) | DSS |
| Awada, 2021 [56] | 183 | 60 (24-93) | 95 (51.2) | 48.5 m | III-IV | various | Belgium, September 2014 - September 2019 | Single-centre exploratory observational study | ALB (ULN), LDH (ULN), CRP (2xULN), ALC (750/mm^3^), ANC (7500/mm^3^), NLR (5) | OS, PFS |
| Boerlin, 2022 [57] | 79 | 62 (24-88) | 28 (35) | 12 m | III-IV | BRAFi/MEKi; immunotherapy (ICI); chemotherapy | Switzerland, October 2015 - March 2021 | Retrospective observational study | ctDNA (BRAF/NRAS mutations, detectable/undetectable) | OS |
| Braune, 2020 [58] | 62 | 66 (28-92) | 21 (34) | NA | III-IV | surgery, BRAFi/MEKi ; immunotherapy and additional theraies | USA, NA | Single centre, nonrandomized, noninterventional, retrospective register study | ctDNA (detectable/undetectable) | PFS |
| B. Weide, 2013 [59] | 499 | 60 (49–70) ‡ | 207 (41.49) | NA | IV | chemotherapy; immunotherapy; biochemotherap | Germany, 2000 - 2010 | Retrospective observational study | LDH (ULN), S100B (ULN) | OS |
| C. Stopa, 2020 [60] | 287 | 63 (18-94) | 114 (60) | 12.6 | IV | various | Poland, 2013 - 2019 | Retrospective observational study | LDH (ULN) | OS, PFS |
| Cananzi, 2013 [61] | 44 | 53 (21-85) | 20 (45.45) | 43.5 m (who remained alive), 9.5 m (who died) | IV | Surgery of intraabdominal metastases | UK, September 1998 - November 2011 | Retrospective, observational study | NLR (5) | OS |
| Chiarion.Sileni, 2003 [62] | 140 | 60 (27-76) | 55 (39.2) | NA | NA | bio-chemotherapy ; chemotherapy | Italy, March 1997 - December 1999 | Prospective, randomized, multicenter study | LDH (NA) | OS |
| D.Desai, 2023 [63] | 334 | 63.0 (13.8) † | 97 (29.04) | NA | IV | NA | US, 1975 - 2017 | Retrospective observational study | LDH (1.5 ULN) | mortality risk |
| Desch, 2016 [64] | 533 | 64 (21-94) | 219 (41.1) | 60 m | I-IV | NA | Germany, NA | Retrospective observational study | CRP (5 mg/l), LDH (250 U/l), S100B (0.105 μg/l) D dimer (0.6 mg/l) | PFS |
| Eton, 2002 [65] | 183 | 49 (11) and 48 (12) | 74 (40.44) | 52 m | III-IV | Sequential Biochemotherapy Versus Chemotherapy | USA, October 1993 - May 1999 | Retrospective observational study | LDH (ULN) | OS |
| Fang, 2015 [66] | 1144 | (44.3-66.3) †† | **492** (43) | 74.8 m | I-IV | NA | USA, March 1998 - August 2009 | Prospective observational study | CRP (10 mg/l), Vitamin- D | OS, MSS, DFS |
| Forthun, 2019 [67] | 26 | 63 (29-77) | 11 (42) | NA | IV | anti-VEGF | Norway, April 2005- August 2009 | Phase II clinical trial | ctDNA (1), LDH (ULN) | OS, PFS |
| Gonzalez-Cao, 2018 [68] | 66 | 58 (28-84) | 34 (52.52) | NA | IV | BRAFi ; BRAFi/MEKi; anti-PD-1; chemotherapy | Spain, 2013 - 2015 | Clinical Trial, national, multi-center translational study | cfDNA BRAFV600 (10.5 pg/μl), ctDNA BRAFV600 (detectable vs undetectable) | OS, PFS |
| Gray, 2014 [69] | 44 | 58 (28-80) | 18 (41) | NA | NA | anti-VEGF; anti-VEGF + immunotherapy (high dose or low dose IFN) | USA, December 2001- April 2012 | Phase II clinical trial | LDH (NA) | PFS |
| Henry, 2013 [70] | 121 | 60.2 (15.6) † | 50 (41.32) | 17 m | I-IV | NA | France, March 2003 - November 2011 | Prospective observational study | S100B (0.15 ng/ml) | PFS, OS |
| Hoejberg, 2012 [13] | 103 | 53 (29-74) | 43 (42) | NA | IV | immunotherapy (IL-2 ± IFN) ± chemotherapy | Denmark, February 1993- December 2003 | Phase II study | IL-6 (4.9 ng/l), LDH (ULN) | OS |
| Hügel, 2016 [71] | 101 | 62.7 (59.9 - 23.3) | 51 (50.5) | 12 m | IV | chemotherapy; immunotherapy (IFN, PEG-intron); chemotherapy+ immunotherapy (IL-2); targeted therapy (sorafenib, imatinib) | Germany, 2007 - 2012 | Prospective observational study | HGF (1602.4 pg/ml), S100B (0.06 μg/dl) | OS, PFS |
| Ibrahim, 2020 [72] | 353 | 63 (17-92) | 124 (65) | NA | IIB-IIIC | various | Canada, 1 January 2006-January 2016 | Retrospective observational study | LDH (ULN) | OS |
| Interno, 2023 [73] | 105 | NA | 44 (42) | NA | NA | BRAFi/MEKi ; chemotherapy ; immunotherapy | NA, 2017 - 2021 | Retrospective observational study | LDH (ULN) | OS |
| Koguchi, 2015 [16] | 123 | 57 (19-88) | NA (36.4) | 31 m | NA | immunotherapy (GP-100 peptide) | USA, NA | Retrospective observational study | LDH (NA), ALC (NA) | OS |
| Kopecky, 2022 [74] | 66 | 66.5 (35-80) | 29 (43.9) | 19.1 m | NA | BRAFi/MEKi ; chemotherapy ; anti-PD-1; anti-CTLA-4; anti-CTLA-4 + anti-PD-1 | Czech Republic, October 2012 – October 2020 | Retrospective observational study | NLR (3), PLR (160), LMR (2), | OS, PFS |
| K.P.Wevers, 2013 [75] | 119 | 58.3 (24.7 - 93.2) | 56 (47.1) | 17 m | III | lymph node dissection | The Netherlands, 2004-2011 | Retrospective observational study | S100B (0.15 and 0.2 µg/l), LDH (250 U/l) | MSS |
| Lee, 2018 [76] | 161 | 52 (19-87) | 84 (52) | 60 m | III | anti-VEGF | UK (multicenter), NA | Retrospective observational study | ctDNA (detectable vs undetectable) | OS, DFI, DMFS |
| Lu, 2018 [49] | 338 | 52.5 (17–86) | 157 (53.6) | NA | III- IV | chemotherapy | USA, NA | Retrospective observational study (BRIM3) | ctDNA BRAFV600 (detectable vs undetectable), cHGF (NA) | OS |
| Marcus, 2014 [77] | 135 | 55.1 (21.6- 92.8) | NA | NA | NA | SRS | NA, 1998 - 2010 | Retrospective observational study | LDH (240 U/l) | OS |
| Meckbach, 2014 [78] | 215 | 64 (NA) | 96 (44.65) | 9 m | IV | chemotheraphy | Germany, 2000 - 2010 | Retrospective observational study | LDH (ULN) | OS |
| Moser, 2019 [79] | 567 | NA | NA | 22.4 m | NA | BRAFi/MEKi; anti-PD-1; anti-PD-1 + anti-CTLA-4 | USA, NsA | Retrospective observational real-world survival study | LDH (ULN) | OS |
| Neuman, 2007 [80] | 122 | 63 (21-91) | 45 (36.9) | 21 m | IV | chemotherapy; immunotherapy; combination therapy | USA, 1995 - 2005 | Prospective observational study | LDH (200 U/l) | OS |
| Partl, 2013 [81] | 87 | 63 (19-86) | 31 (35.6) | NA | IV | WBRT | Austria, 1988 - 2009 | Retrospective observational study | LDH (240 U/l) | OS |
| Partl, 2015 [82] | 234 | 64 (20-87) | 86 (36.8) | NA | IV | WBRT | Austria, 1988 – 2013 | Retrospective observational study | LDH (ULN) | OS |
| Reinert, 2020 [83] | 107 | 65 NA | 52 (58.6) | NA | I-IV | surgical, systemic | Germany, April 2013 - January 2015 | Retrospective observational study | LDH (250 U/l), CRP (0.5 μg/l), ALP (130 U/l), S100B (0.1 μg/l), | OS |
| Rogala, 2022 [24] | 33 | 57 (24-89) | 106 (51.2) | 16.8 m | III-IV | BRAFi/MEKi+ anti-PD-1 sequence | Poland, December 2015 - January 2022 | Retrospective observational study | LDH (ULN) | OS, PFS |
| S Kruijff, 2011 [84] | 62 | 56.9 (24.7- 93.2) | 32 (51.6) | NA | III | therapeutic lymph node dissection (TLND) | The Netherlands, February 2004 - December 2007 | Prospective observational study | S100B (0.15 mg/l) | DFS, DSS |
| Keilholz, 1996 [85] | 72 | NA | 30 (41.66) | NA | NA | immunotherapy (IFN, IL-2) | NA, 1987 -1992 | Retrospective observational study | LDH (240 U/l), | OS |
| S Valpione, 2018 [86] | 43 | 58.1 (18-85.1) | 18 (42) | 11.9 m | IV | BRAFi/MEKi; anti-PD-1; anti-CTLA-4; chemotherapy | UK, NA | Prospective observational study | **LDH (550 U/l)** | OS |
| Schmidt, 2005 [87] | 85 | 51 (29-70) | 37 (43.5) | NA | NA | immunotherapy (IL-2, IFN) ± chemotherapy | Denmark, February 1997 - March 2001 | Retrospective observational study | S100B (NA), LDH (NA), MART1-mRNA (NA), Tyrosinase-mRNA | OS |
| Soubrane, 2005 [88] | 125 | 46 (18-70) | 52 (41.60) | NA | NA | chemotherapy + immunotherapy (IL-2, IFN) | France, 1994 - 2002 | Retrospective observational study | IL-6 (5 pg/ml) | OS |
| Taques, 2020 [89] | 19 | 61 NA† | 12 (63.2) | 4.27 m | III, IV | NA | Brazil, March- November 2018 | Prospective observational study | ctDNA (detectable vs undetectable) | PFS |
| Tolmeijer, 2021 [90] | 53 | 61 (28-79) | 19 (36) | 12.3 m | IV | BRAFi/MEKi or immunotherapy (ICI) | The Netherlands, March 2017 - June 2020 | Prospective observational study | ctDNA (detectable vs undetectable) | PFS |
| Váraljai, 2021 [91] | 166 | 61 | 67 (40.3) | NA | III | various | Germany, NA | Retrospective observational study | ctDNA (23.6 ng/ml) | OS, PFS |
| Vereecken, 2009 [92] | 29 | 60(33–92) | 20 (68.9) | NA | III-IV | NA | Belgium, NA | Retrospective observational study | Gal-3 (8, 8-10, ›10 ng/ml), S100BB (0.1 μg/l), LDH (480 UI/l), CRP (1 mg/l), Gal-3 (8, 8-10, ›10 ng/ml), S100BB (0.1 μg/l), LDH (480 UI/l), CRP (1 mg/l) | OS |
| Vereecken, 2009 [92] | 54 | 59 (31–91) | 33 (61.1) | NA | III-IV | NA | Belgium, NA | Retrospective observational study | Gal-3 (8, 8-10, ›10 ng/ml), S100BB (0.1 μg/l), LDH (480 UI/l), CRP (1 mg/l), Gal-3 (8, 8-10, ›10 ng/ml), S100BB (0.1 μg/l), LDH (480 UI/l), CRP (1 mg/l) | OS |
| Yu, 2017 [93] | 226 | NA | NA | NA | NA | immunotherapy (IFN) | China, October 2010 - October 2016 | Retrospective observational study | LDH (ULN), Lymphocyte cells count (1.8x10^10), Neutrophil cells count (4x10^9), NLR (2.35), PLR (129) | OS |
| Zeijl, 2020 [94] | 1532 | 62 (19-94) | 607 (39.6) | NA | III  -IV | immunotherapy (ICI) ; BRAFi/MEKi | The Netherlands, 2014 - 2017 | Prospective observational study | LDH (ULN) | OS |
| Zhang, 2021 [95] | 60 | 57 (30-80) | 28 (46.7) | NA | I-IV | immunotherapy ± targeted therapy; chemotherapy ± targeted therapy | China, April 2018 - September 2020 | Prospective observational study | HSP90α (NA), LDH (NA) | PFS |
| Articles in the systematic review | | | | | | | | | | |
| A. Porter, 2001 [96] | 218 | 49 (18–80) | 97 (44.50) | 43 m | NA | wide local excision and sentinel lymph node biopsy | US, 1994 -1997 | Prospective observational study | IL-4 (7.8 pg/ml), IL-6, IL-10, IFN-g, TNF-a (15.6 pg/ml) | **5-year DFS** rates |
| Aglietta, 2023 [97] | 599 | 53 (23-90) | 230 (38.4) | NA | I-IV | BRAFi/MEKi | Italy, 2013-31^st^ October 2017 | Retrospective observational real-world study | LDH (ULN) | OS |
| Allen, 2011 [54] | 39 | NA | 17 (43.59) | NA | IV | BRAFi/MEKi alpha-therapy | Australia, 12t^h^ August 2009 | Phase I clinical trial | MIA (22 ng/dl) | OS |
| B. Weide, 2012 [98] | 855 | 62 (50-71) | 373 (43.63) | 9-25 m | I-IV | **s**urgical removal of metastases; **other treatments** | Germany, 1996 - 2010 | Retrospective observational study | LDH (ULN), S100B (ULN) | OS |
| Boerlin, 2022 [57] | 79 | 62 (24-88) | 28 (35) | 12 m | III-IV | BRAFi/MEKi; immunotherapy (ICI); chemotherapy | Switzerland, October 2015 - March 2021 | Retrospective observational study | CRP (ULN), S100B (ULN) | OS, PFS |
| Bolander, 2008 [99] | 98 | NA | 44 (44.90) | 68 m | NA | surgical resection | Sweden, NA- June 2006 | Retrospective, observational study. | combined S100BA1B + S100BBB (150 ng/l), S100BA1B (50 ng/l), S100BBB (50 ng/l) | OS, DSS |
| Cananzi, 2014 [61] | 44 | 53 (21-85) | 20 (45.45) | 43.5 m (who remained alive), 9.5 m (who died) | IV | Surgery of intraabdominal metastases | UK, September 1998 - November 2011 | Retrospective, observational study | LDH (ULN) | OS |
| Cao, 2007 [100] | 42 | 49 (26-73) | 19 (45.24) | NA | IV | chemotherapy + immunotherapy (IL-2 + IFN) | Spain, December 1996 - December 2002 | Retrospective observational study from two Phase II clinical trials | S-100 (0.20 µg/l, MIA (14 ng/ml), LDH (450 U/l), β2-μglobulin (2.5 mg/l) | DFS, OS |
| Chung, 2002 [101] | 70 | 51.5 (19-82) | 26 (37.17) | 25 m | NA | surgical resection | NA, August 1985 - October 1999 | Retrospective observational study | TA90 Immune Complex (TA90-IC) (Optical Density (OD) ≥ 0.410 at 405 nm on ELISA) | DFS, OS, recurrence rate |
| Corrie, 2018 [102] | 1343 | 56 (18-88) | 590 (43.93) | 76.8 m | II-IIIB | anti-VEGF | UK, July 2007 – March 2012 | Retrospective observational study (AVAST-M tria) | LDH (ULN), VEGF-R1 (NA) | OS, DFI, DMFI |
| Cybulska-Stopa, 2012 [103] | 427 | 51.4 (18-82) | 191 (44.73) | NA | III-IV | anti-PD-1; anti-CTLA-4; BRAFi; BRAFi/MEKi | Poland, 1995 - 2005 | Retrospective observational study | LDH (ULN), Aspartate Aminotransferase (NA), Alanine Aminotransferase (NA), Hemoglobin (NA) | OS, PFS |
| Damuzzo, 2016 [8] | 44 | 63 (33-83) | F15 (65.9) | NA | IV | anti-CTLA-4 | Italy, NA | Prospective observational study | IL-6 (NA) | OS |
| Deckers, 2020 [104] | 52 | 64 (53-59) ‡ | 22 (42.30) | 24.9 m | IV | NA | The Netherlands, 2010 - 2015 | Retrospective observational study | LDH (250 U/I), S100B (0.15 mg/l) | DSS |
| Decoster, 2015 [105] | 39 | 55 (31-84) | 18 (46.15) | NA | IV | targeted therapy (TKI) | Belgium, July 2008 - July 2009 | Phase II clinical trial | VEGF (continuous variable; mean: 186.3 ng/l), VEGFR1 (Mean: 101.4 ng/l), VEGFR2 (mean: 8920.6 ng/l), PlGF (mean: 46.5 ng/l) | PFS, OS, response |
| Domingo Doménech, 2007 [106] | 97 | 47.8 (22-72) | 50 (51.55) | 62.9 m | IIB-IIIC | immunotherapy (high-dose IFN-α2b) | Spain, NA | Prospective observational study | S100B (0.15 µg/l) | DFS, OS, relaps |
| Egberts, 2008 [107] | 105 | 56 (29-79) | 42 (0.40) | NA | IV | chemotherapy± immunotherapy (IFN, IL-2) | Germany, 1994 - 2001 | Retrospective observaional study | LDH (250 U/l), S100B (0.2 μg/dl) | OS |
| Fang, 2016 [108] | 1042 | 54.8 (44.2-66) | 452 (43.38) | 86.4 m | NA | NA | USA, August 1997 - August 2009 | Prospective observational study | Vitamin D (deficient: <20 ng/ml, insufficient: 20–30 ng/ml, sufficient: ≥30 ng/ml; cut-off for survival analysis: 16 ng/ml)  CRP (continuous variable) | OS, DSS, DFS |
| Faries, 2004 [109] | 75 | NA | 29 (38.66) | 50 m | III | immunotherapy (vaccine) | USA, NA | Retrospective observational study | MIA (8.5 ng/ml) | OS, recurrance |
| Felix, 2016 [110] | 77 | 60.4 (17.4) † | 35 (45.45) | 28 m | IIIC-IV | anti-CTLA-4 | France, June 2010 - September 2013 | Prospective observational study | LDH (500 U/l), S100B (0.15 µg/l), MIA (7.5 µg/l), soluble MICA (sMICA) (45 µg/l), Anti-MICA antibodies (Fluorescence intensity >2) | OS, response |
| Filia, 2013 [111] | 158 | NA | 73 (45.4) | NA | I/II-III | NA | UK, NA | Pilot study | OPN (49.5 ng/ml and 64.34 ng/ml) | OS, DSS |
| Foppen, 2016 [112] | 39 | 52.9 (26-84) | 16 (41.03) | NA | NA | BRAFi; BRAFi/MEKi ± anti-CTLA-4; anti-CTLA-4 ; chemotherapy + anti-CTLA-4 | The Netherlands, May 2010 -March 2015 | Retrospective observational study | LDH (248 U/l), S100BB (0.10 µg/l) | OS |
| Forschner, 2020 [113] | 19 | 51 (43-57) ‡ | 11 (57.89) | 0.936 m | IV | BRAFi/MEKi | Germany, January 2018 - NA | Prospective observational study | ctDNABRAFV600E/K (Presence vs. absence), LDH (248 U/l), S100B (0.10 µg/l) | PFS, OS, response |
| Fusi, 2009 [114] | 299 | 48 (18-71) | 120 (40.13) | 43.2 m | III | immunotherapy (IFN) | Germany, Belgium, Italy, The Netherlands, UK, France, November 2000- March 2006 | Prospective biomarker sub-study (EORTC 18991 trial) | CTC: Tyrosinase mRNA (RT-PCR), Mart-1/Melan-A mRNA (4.4 × 10⁻⁵ ratio to housekeeping gene PBGD) | DMFS, recurrence |
| Guida, 2003 [115] | 176 | 59 (26-76) | 71 (40.34) | 18 m | NA | chemotherapy± immunotherapy (IFN, IL-2) | Italy, 1^st^ March 1997- 31^st^ December 1999 | Phase III randomized multicenter trial | LDH (460 U/l), CRP (1.5 mg/dl), Fibrinogen (450 mg/dl), Leukocytes, Lymphocytes, Eosinophils, ESR | OS |
| Guida, 2006 [116] | 90 | 59 (26-76) | NA | 43.2 m | NA | chemotherapy ± immunotherapy (IFN, IL-2) | Italy, 1997-1999 | Prospective observational study | L-6, IL-8, IL-10, IL-12, soluble IL-2 receptor (terciles, NA) | OS |
| Henry, 2010 [117] | 90 | 59.9 (23-92) | 37 (41.11) | 18 m | I-IV | NA | France, March 2003 -March 2008 | Prospective observational study | p-proteasome (4300 ng/ml) | risk of pression, risk of death |
| Henry, 2013 [70] | 121 | 60.2 (15.6) † | 50 (41.32) | 17 m | I-IV | NA | France, March 2003 - November 2011 | Prospective observational study | p-proteasome (387 ng/ml), MIA (6.5 ng/ml), LDH 240 (UI/l) | PFS, OS |
| Hoffmann, 2011 [118] | 138 | 52.7 (1.2) † | 62 (44.93) | NA | III | Surgical | Germany, 1997-2002 | Prospective observational study | LDH (240 U/l), MIA (12 ng/ml) | relapse, recurrence, OS |
| Hoshimoto, 2012 [119] | 320 | 51.1 (18-81) | 125 (39.06) | 51.7 m | NA | immunotherapy (BCG/CanVaxin) | USA, 1998 - June 2010 | Phase III multicenter prospective study | CTCs detected by ddPCR: **MART-1, MAGE-A3,** and **GalNAc-T (≥2 markers positive** vs. **0–1 marker positive)** | DMFS, RFS, DSS |
| Hu, 2022 [120] | 161 | 68 (22-84) | 60 (37.27) | NA | IV | Immunotherapy (ICI); BRAFi/MEKi; immunotherapy (ICI) + BRAFi/MEKi | Australia 28^th^ May 2010 – 3^rd^ November 2017 | Retrospective observational study | LDH (ULN), NLR (3) | OS |
| Iivanainen, 2019 [121] | 44 | 66 (NA) | 15 (34.09) | NA | III-IV | anti-PD-1 | Finland 2014 - 2018 | Retrospective observational study | NLR (5), total leukocytes, total lymphocytes, LDH (ULN), CRP (9.5 mg/ml) | PFS, OS |
| J.van Not, 2023 [122] | 1278 | 61 (52-70) | 476 (37.25) | 30.4 m | IV | Immunother therapies  otherapy (ICI) | The Netherlands, 2013 - 2020 | Retrospective observational study | LDH (250–500, 500–750, ≥750) | OS |
| Keilholz, 1996 [85] | 72 | NA | 30 (41.66) | NA | NA | immunotherapy (IFN, IL-2) | NA, 1987 -1992 | Retrospective observational study | ALP (180 U/l) | OS |
| Keller, 2019 [123] | 22 | 66 (43-89) | 12 (54.54) | 30.4 m | IIIC-IV | anti-PD-1; anti-CTLA-4 ; anti-PD-1+ anti-CTLA-4, | France, February 2014 - November 2016 | Retrospective observational study | ctDNA (increase or decrease, 20 change) | OS, response |
| Khoja, 2013 [124] | 101 | 60 (32-98) | 48 (47.52) | 5.9 m | IV | chemotherapy ; anti-CTLA-4; BRAFi/MEKi | UK, December 2009 - December 2011 | Prospective observational study | CTC (2 positive/ 7.5ml) | OS, progression, response |
| Khoja, 2016 [15] | 183 | 58 (24-89) | 68 (37.16) | 7.5 m | III-IV | anti-CTLA-4 | Canada, 2008 - 2015 | Retrospective observational study | LDH (440 U/l), PLR (NA), ELR (NA) | OS in prognostic groups, PFS |
| Koyanagi, 2010 [125] | 87 | 48 (18-73) | 27 (31.03) | 15.4 m | IV | chemotherapy + immunotherapy | USA, NA | Prospective, multicenter phase II clinical trial | CTC by multimarker RT-qPCR:  (MART-1, GalNAc-T, PAX-3, MAGE-A3, Mitf.; detection of ≥1 markers) | PFS, OS |
| Kruijff, 2009 [126] | 56 | 57 (24-93) | 29 (51.76) | 14.2 m | IIIb-IIIc | **therapeutic lymph node dissection** | The Netherlands, January 2004 – January 2008 | Prospective observational study | S100B (0.15 µg/l), LDH (250 U/l) | DFS |
| Lee, 2019 [127] | 119 | 64 (20-90) | 41 (34) | 26 m | III, IV | surgical | Australia, October 2010 - July 2017 | **Prospective observational study** | ctDNA (detectable vs undetectable), LDH (ULN) | MSS |
| Levati, 2022 [128] | 57 | NA (20-82) | 20 (35.09) | NA | IIIc-IV | BRAFi; BRAFi/MEKi | Italy, 2013 - 2018 | Retrospective observational study | miR-1246 (8.637, determined by ROC analysis); miR-485-3p (0.013), miR-1246/miR-485-3p ratio (345.493) | PFS, OS |
| Li, 2014 [129] | 72 | NA | 37 (52.4) | NA | I-IV | NA | China, **May 2004 – June 2013** | Prospective observational study | miR-221 measured by RT-qPCR (median value 2.95) | OS, RFS |
| Li, 2018 [130] | 100 | 51 | 38 (38) | 26 m | I-IV | Immunotherapy (IFN) | China, January 2010 – December 2014 | Prospective observational study | CTC (6 CTCs per 7.5 ml blood), S100B (0.19 µg/l) | DSS, PFS |
| Li, 2021 [131] | 22 | 62.1 (14) | 8 (36.36) | 36 m | I-III | NA | China, January 2014 – December 2017 | Prospective observational study | MIA (914.7 pg/ml), LDH (285.5 U/l) | OS |
| Li, 2021 [131] | 22 | 60.1 (15.7) † | 11 (50) | 36 m | I-III | NA | China, January 2014 – December 2017 | Prospective observational study | MIA (914.7 pg/ml), LDH (285.5 U/l) | OS |
| Lin, 2018 [132] | 44 | 60 (24-81) | 10 (23) | NA | II-IV | Immunotherapy (bacillus Calmette-Guérin plus Canvaxin melanoma vaccine) | USA, NA | Prospective observational study | cfDNA Single Nucleotide Variants (SNVs): number of unique SNVs ("SNV load": >2 SNVs); total cumulative variant allele fraction ("SNV burden":>0.5 total cumulative VAF)  Copy Number Variations (CNVs): EGFR, ERBB2, and MET amplifications | OS, DFS |
| Lu, 2023 [133] | 54 | 60.5 (530-84) | 15 (27.77) | 38.4 m | III-IV | anti-PD-1, anti-PD-1 + anti-CTLA-4 | USA, October 2008 - October 2017 | Retrospective observational study | PD-1 level (≥ 2.22 pg/ml)  PD-L1 level (≥ 1.36 pg/ml)  PD-1/PD-L1 ratio (≥ 1.67 pg/ml) | mortality risk |
| Lucci, 2020 [134] | 243 | 57 (20-88) | 94 (38.6) | 17 m | III-IV | chemotherapy; targeted therapy; immunotherapy (anti-PD-1, IFN, IL-2, GP-100) | USA, February 2012 - June 2017 | Prospective observational study | CTC (≥1 CTC; ≥2 CTCs and ≥3 CTCs/ 7.5 ml) | RFS |
| Lucci, 2023 [135] | 325 | 54.3 (20–89) | 151 (46.5) | 48.3 m | III | immunotherapy; targeted therapy | USA, January 2012 – February 2022 | Prospective observational study | CTC (≥1 CTC/7.5 ml) | RFS |
| M Staudt, 2010 [136] | 265 | 58 (22) †† | 111 (41.9) | 5 m | IV | chemotherapy; neurosurgical operation | Germany, October 1986 – March 2003 | Retrospective observational study | LDH (2 x ULN) | OS |
| M.F.Sanmamed, 2017 [137] | 29 | 57.7 (33-79) | 15 (51.7) | 15 m | NA | anti-PD-1, anti-CTLA-4 +anti-PD-1 | USA (multicenter), Spain, November 2008 – May 2016 | Prospective observational study | IL-8 (early >9.2 decrease) | OS, response |
| Mahalingam, 2014 [138] | 14 | 61 (43-77) | 9 (64.29) | NA | IV | anti-VEGF+ targeted therapy (TKI) | USA, NA | Phase II clinical trial | VEGF (300 pg/ml), VEGFR1 and VEGFR2 (median-based cut-off, high/low) | TTP, PFS |
| Meral, 2001 [139] | 35 | 52.2 (31–80)§§ | 14 (40) | NA | III-IV | chemotherapy ; immunotherapy (IFN) | Turkey, NA | Prospective phase II study | MIA (18.7 ng/ml), S100B (0.08 µg/l), LDH (**453.7 U/l**) | OS |
| Pelizzari, 2023 [140] | 150 | 60.4 NA | 49 (32.7) | 33.8 m | NA | various | Italy, April 2007 – December 2018 | Retrospective observational study | LDH (2 x ULN) | OS |
| Prasmickatie, 2015 [141] | 275 | NA | 100 (36) | NA | II-III | Immunotherapy (IFN) | Multicenter, 1996 - 2004 | Retrospective observational cohort (RCT) | Osteopontin (OPN) (25 ng/ml) | PFS, OS |
| RAO, 2011 [142] | 44 | 59 (31-81) | 19 (43.18) | 10.1 m | III-IV | NA | USA, NA | Retrospective observational cohort | CTC (‹2 cell / 7.5 ml) | OS |
| S Diem, 2016 [143] | 66 | 56.2 (49.1-66.8) | 25 (37.9) | 9 m | IV | anti-PD-1 | UK, 2013 -2014 | Retrospective observational cohort | LDH (ULN and >10 from baseline) | OS, PFS |
| S Hall, 2018 [144] | 93 | 55.5 (27-86) | 32 (34) | 17 m | IV | Immunotherapy (ICI, IFN IL-2, vaccine); BRAFi/MEKi ; chemotherapy | USA, February 2012 - August 2017 | Prospective observational cohort | CTCs (›1 cell / 7.5 ml) | PFS |
| S Valpione, 2018 [86] | 43 | 58.1 (18-85.1) | 18 (42) | 11.9 m | IV | BRAFi/MEKi; anti-PD-1; anti-CTLA-4; chemotherapy | UK, NA | Prospective observational study | cell-free DNA (cfDNA) (89 pg/ml); a **cfDNA/tumor volume ratio** (shedding coefficient) cut-off **8 pg/(ml*cm³)** | OS |
| Sanmamed, 2014 [145] | 18 | 51 (11.9)† | 5 (27.77) | 14 m | IIIC-IV | BRAFi | Spain, April 2011 – April 2013 | Prospective observational study | LDH (536 U/l), MIA (**9 μg/l**), S100B (0.1 μg/l) | PFS |
| Sanmamed, 2015 [146] | 20 | 50 NA | 7 (35) | 14 m | IIIC-IV | BRAFi | Spain, April 2011 – May 2014 | Prospective observational study | cell-free BRAFV600E (cfBRAFV600E) (**Exploratory cut-off** 216 copies/ml) | OS, PFS |
| Schildbach, 2023 [147] | 138 | 59 | 67 (48.6) | 53.4 m | III | immunotherapy (IFN) | Germany, January 2011- 1st January 2022 | Retrospective observational study | LMR (3.5), CRP (3), dNLR (2), NLR (3.5), PLR (NA) | OS, RFS |
| Schmidt, 2006 [148] | 234 | 56 (25-86) | NA | 66 m | I-II | surgery | NA, February 1997 - December 2002 | Retrospective observational study | YKL-40 | PFS, OS |
| Schneider, 2021 [149] | 30 | 62 (43-84) | 11 (36.66) | NA | IV | surgery | Germany, 2013 - 2019 | Retrospective observational study | CRP (10 mg/dl), WBC (12 g/l), platelet count (300 g/l), neutrophil count (6.5 g/l), preoperative monocyte (0.65 g/l), LMR (2), NLR (4), PLR (145) | OS |
| Schouwenburg, 2018 [150] | 626 | 59 (23-90) | 277 (44) | 35.8 m | III, IV | BRAFi | The Netherlands, July 2012 - June 2015 | Retrospective observational study | LDH (ULN) | OS |
| Seetharamu, 2012 [151] | 181 | 63 (NA) | 72 (40) | 42.7 m | IV | NA | USA, August 2002 – June 2008 | Prospective observational study | LDH (313–618 U/l), albumin (3.5–5.0 g/dl) | OS |
| Shah, 2021 [152] | 158 | 56 (21–90) | 69 (43.67) | NA | NA | BRAFi; BRAFi/MEKi | USA, August 2002 – June 2008 | Retrospective observational study | LDH (NA), Absolute Lymphocyte Count (ALC)/Absolute Neutrophil Count (ANC) Ratio (NA) | OS, PFS |
| Silva, 2018 [153] | 83 | 57 (20-87) | 36 (43.4) | NA | I-IV | NA | UK, NA | Prospective observational study | CNAS (copy-number aberration score) in cfDNA (75th percentile) | OS |
| Sivan, 2012 [154] | 49 | NA | 25 (51) | 23 m | II-IV | immunotherapy (vaccine) | Israel, 1998 - 2010 | Retrospective observational study | Serum CEACAM1 (median value, NA), S100B (NA), LDH (ULN) | OS |
| Smit, 2005 [155] | 145 | 48 (11.8) † | 51 (35.17) | NA | IV | chemotherapy ± immunotherapy (cytokine based, IL-2, IFN) | The Netherlands, October 1998 – November 2002 | Prospective observational study | S100B (0.16 µg/l), LDH (ULN) | OS, response |
| Tan, 2019 [156] | 133 | 57 (22-93) | 67 (50.3) | 18 m | III, IV | immunotherapy (ICI) | Australia, March 2011 - December 2016 | Retrospective observational study | ctDNA (detectable/ undetectable; ≥ 1 copy is detectable) | OS, RFS |
| Tarhini, 2009 [157] | 670 | 51 (19-81) | 246 (36) | 93.6 m | IIB-III | immunotherapy (ganglioside vaccine, IFN) | USA, NA | Retrospective biomarker analysis (ECOG 1694) | S100B (0.15 µg/l) | OS, RFS |
| Tartour, 1996 [158] | 81 | 50 (20-66) | 42 (51.85) | NA | IV | immunotherapy (IL-2) | France, NA | Prospective observational study | CRP (10 mg/l), IL-6 (20 pg/ml), LDH (320 IU/l) | OS, response |
| Tas, 2005 [159] | 16 | 51 (27-72) | 6 (37.50) | NA | IV | chemotherapy | Turkey, NA | Prospective observational study | IL-6 (median: 30.55 pg/ml), TNF-α (median 64.95 mIU/ml), EPO (median 31.85 mIU/ml) | OS |
| Tawbi HA, 2022 [160] | 267 | 56 (46-66) | 119 (44.6) | 27.2 m | NA | immunotherapy (ICI) + BRAFi/ MEKi; BRAFi/MEKi | Multicenter, 2017 – 2020 | RCT | **(ctDNA)** detectable vs undetectable, CD4+/CD8+ ratio (2.9) | OS, PFS |
| Tawbi HA, 2022 [160] | 265 | 55 (47-65) ‡‡ | 106 (40) | 27.2 m | NA | immunotherapy (ICI) + BRAFi/ MEKi; BRAFi/MEKi | Multicenter, 2017 – 2020 | RCT | (ctDNA) detectable vs undetectable, CD4^+^/CD8^+^ ratio (2.9) | OS, PFS |
| Ugurel, 2001 [161] | 125 | 73 (28-82) | 29 (42.64) | NA | NA | chemotherapy; immunotherapy (IFN) | Germany, September, 1997 – November 1998 | Retrospective observational study | angiogenin, bFGF, VEGF, IL-8 | OS, PFS |
| Umemura, 2017 [162] | 140 | 64.1 NA † | 71 (50.71) | 24.2 m | I-IV | NA | Japan, June 2007 - June 2015 | Retrospective observational study | 5-S-CD (standard: 10.0 nmol/l, prognostic: 15.0 nmol/l)., LDH (222 IU/l) | MSS |
| Wilgenhof, 2013 [163] | 50 | 50 (31-80) | 22 (44) | 24.9 m | IV | anti-CTLA-4 | Belgium, April 2010 - May 2011 | Retrospective observational study | CRP (ULN), ALC (800/mm³), LDH (ULN) | OS |
| Wilson, 2019 [164] | 304 | 60 (18-93) | 102 (33.55). | 25 m | I-III | immunotherapy; targeted therapy; chemotherapy | US, August 2002 - December 2015 | Prospective observational study | **LDH** (normal vs. high) | OS |
| Wu, 2017 [165] | 48 | 67 (26-86) | 18 (37.5) | 33 m | III- IIIC | anti-PD-1 | USA, NA | Retrospective biomarker analysis of prospective clinical trial | Angiopoietin-2 (ANGPT2)  Pretreatment (3175 pg/ml)  Post-treatment change (fold change of 1.25) | OS, response |
| Wu, 2017 [165] | 43 | 59 (36-85) | 24 (55.8) | 33 m | III- IIIC | anti-PD-1+ anti-VEGF | USA, NA | Retrospective biomarker analysis of prospective clinical trial | Angiopoietin-2 (ANGPT2)  Pretreatment (3175 pg/ml)  Post-treatment change: fold change of 1.25 | OS, response |
| Wu, 2018[166] | 43 | NA | NA | NA | NA | anti-PD-1; anti-CTLA-4; anti-CTLA-4 + anti-VEGF | USA, NA | Prospective observational study | Gal-3 (7.088 ng/ml) | OS |

**Abbreviations:** AJCC: American Joint Committee on Cancer; ALB: albumin; ALP: alkaline phosphatase; ALC: absolute lymphocyte count; ANC: absolute neutrophil count; ANGPT2: angiopoietin-2; BM: brain metastasis; BRAFV600E: BRAF V600E mutation; CCL2: C-C motif chemokine ligand 2; CD: cluster of differentiation; CEACAM1: carcinoembryonic antigen-related cell adhesion molecule 1; CHI3L1: chitinase-3-like protein 1; CI: confidence interval; CNAS: copy number alteration score; CNV: copy number variation; CRP: C-reactive protein; CTC: circulating tumor cell; ctDNA: circulating tumor DNA; DFS: disease-free survival; DFI: disease-free interval; DMFI: distant metastasis-free interval; DMFS: distant metastasis-free survival; DSS: disease-specific survival; ECOG: Eastern Cooperative Oncology Group; EGFR: epidermal growth factor receptor; EPO: erythropoietin; ESR: erythrocyte sedimentation rate; HGF: hepatocyte growth factor; HR: hazard ratio; HSP90α: heat shock protein 90 alpha; IFN: interferon; IL-2: interleukin-2; IL-4: interleukin-4; IL-6: interleukin-6; IL-8: interleukin-8; IL-10: interleukin-10; IL-12: interleukin-12; IL-18: interleukin-18; LDH: lactate dehydrogenase; LMR: lymphocyte-to-monocyte ratio; MAGE-A3: melanoma antigen gene-A3; MAPK: mitogen-activated protein kinase; MART-1: melanoma antigen recognized by T cells 1; MCP-1: monocyte chemoattractant protein-1; MEK: mitogen-activated protein kinase kinase; MET: mesenchymal-epithelial transition factor; MHC: major histocompatibility complex; MICA: MHC class I polypeptide-related sequence A; MIA: melanoma inhibitory activity; MM: malignant melanoma; MSS: melanoma-specific survival; NA: not available; NL: Netherlands; NLR: neutrophil-to-lymphocyte ratio; NPT: neopterin; OR: odds ratio; OS: overall survival; PD: progressive disease; PD-1: programmed cell death protein 1; PDL-1: programmed death-ligand 1; PEG-Intron: pegylated interferon alfa-2b; PFS: progression-free survival; PIGF: placental growth factor; PLR: platelet-to-lymphocyte ratio; PR: partial response; PS: performance status; RECIST: Response Evaluation Criteria in Solid Tumors; RFS: recurrence-free survival; RT-PCR: reverse transcriptase polymerase chain reaction; RT-qPCR: quantitative reverse transcriptase PCR; S100: S100 calcium-binding protein; SD: standard deviation; SLN: sentinel lymph node; SNV: single nucleotide variant; SRS: stereotactic radiosurgery; TA90-IC: Tumor associated antigen 90 immune complex; TKI: tyrosine kinase inhibitor; TNF-α: tumor necrosis factor-alpha; TPO: thyroid peroxidase; TTP: time to progression; UK: United Kingdom; ULN: upper limit of normal; VAF: variant allele frequency; VEGF: vascular endothelial growth factor; WBC: white blood cell count; WBRT: whole-brain radiotherapy; YKL-40: human cartilage glycoprotein-3

# **Systematic review results not eligible for quantitative synthesis**

## **Supplementary Table S2:** Serum biomarker level associations with melanoma patient outcomes

| **Biomarker**  **First author, year** | **Cut-off** (time point if not baseline) | **Main result, conclusion** |
| --- | --- | --- |
| **Serum Enzyme Biomarkers** | | |
| **LDH** | | |
| A. Wilson, 2019 [164] | NA | High LDH level was an independent predictor of worse OS (HR: 1.65 (95% CI: 1.25–2.20), P=.001). |
| Aglietta, 2023 [97] | ULN | **Elevated LDH** at baseline was associated with **shorter PFS (5.3 vs. 9.9 months).** |
| B. Weide, 2012 [98] | ULN | Elevated LDH (HR: 1.6 (95% CI: 1.3–2.0), P<.001) was an independent prognostic factor in stage IV MM for OS and DMFS. |
| Cananzi, 2014 [61] | NA | Elevated LDH levels after surgery were linked to worsened OS (P=.008). |
| Cao, 2007 [100] | 450 U/L | **LDH levels were not a significant prognostic marker** for OS and DFS, or monitoring response/progression (P>0.05). |
| Braune, 2020 [58] | 225 U/L | Elevated levels detected 53.6% of active disease cases vs ctDNA levels 76.8%. |
| Corrie, 2018 [102] | ULN | LDH levels neither baseline or after fitting as a time-dependent continuous covariate were not found to be prognostic of DFI, DMFI nor OS. |
| Cybluska-Stopa, 2012 [103] | ULN | Elevated LDH (HR: 1.70 (1.22–2.41) P=.0023) was an independent negative prognostic factor for OS (median OS 5.2 vs 9.6 months (P<.0001)). |
| Deckers, 2020 [104] | 250 U/l | Elevated LDH was predictive for shorter OS (P=.026; median OS 6.7 vs. 28.9 months (P=.019)). |
| Egberts, 2008 [107] | 250 U/L | Elevated LDH levels were significantly associated with **shorter OS (median OS: 10.2 vs. 3.7 months).** It was not significant in multivariate analysis. |
| Felix, 2016 [110] | 500 U/l | Low LDH (9.7 vs. 5.1 months (P=.0001)) levels at baseline and week 3-6 correlated with better response and OS. |
| Foppen, 2016 [112] | 248 U/l | Elevated LDH (OS 3.1 vs. 18.6 weeks, P<.001)) was associated with worsened OS |
| Forschner, 2020 [113] | NA | Elevated LDH (P=.034) levels at baseline were associated with worse OS. |
| Guida, 2003 [115] | 460 U/L | High LDH (HR 1.89 (95% CI: 1.17–3.06, P=.009)) levels were independently predicted worse OS. |
| Henry, 2013 [70] | 230 IU/l | Elevated LDH levels were significantly associated with reduced OS. |
| Hofmann, 2011 [118] | 240 U/L | LDH levels were elevated in patients experiencing relapse (244.71 U/L vs. 162.55 U/L (P=.028)). |
| Hu, 2023 [120] | ULN | Elevated LDH was an independent prognostic factor for OS. |
| Ivanainen, 2019 [121] | NA | LDH levels were not predictive for PFS and OS. |
| J. van Not, 2023 [122] | 250–500, 500–750, ≥750 | LDH was the strongest prognostic factor; elevated LDH was significantly associated with worse OS (median OS: 15.4 months vs. 5.1 months; P<.001). |
| Khoja, 2013 [124] | 550 U/L | Elevated LDH level was associated with worsened OS in univariate analysis. |
| Khoja, 2016 [15] | 440 U/L | LDH (HR = 1.00 (P<.0001)) was an independent prognostic biomarker in ipilimumab-treated MM for OS. |
| Kruijff, 2009 [126] | NA | LDH was not significant for DFS. |
| M. Staudt, 2010 [136] | normal/elevated | LDH levels were independent predictors of OS in patients with BM. (HR: 2.7 (95% CI: 1.8–4.0) for ≤2× ULN; HR: 7.3 (95% CI: 3.7–14.3) for >2× ULN vs. normal; both P<.001) |
| Meral, 2001 [139] | 453.7 U/L | Elevated LDH levels before treatent were associated with shortened OS. |
| Pelizzari, 2023 [140] | Normal, 1 - 2 x ULN, ≥2 x ULN | Elevated LDH (≥2 x UNL) had independent prognostic value in patients with BM for OS (HR: 1.83 (95% CI: 1.07–3.11), P=.02). |
| S. Diem, 2016 [143] | ULN and change (10%) | Baseline LDH level and early LDH change (>10% and ≤10% increase: OS 4.3 vs. 15.7 months (P<.006)) predicted OS and treatment response. Increasing LDH levels indicated poor prognosis on-treatment. |
| Sanmamed, 2014 [145] | 436 U/L | High baseline LDH levels were associated with poor prognosis in BRAFMEKi-treated patients. |
| Seetharamu, 2012 [151] | 320 UI/L | LDH independently predicted OS (median OS: normal LDH 15.5 vs. high 9.6 months, P=.0004). |
| Shah, 2020 [152] | NA | LDH independently predicted PFS and OS (HR: 1.17, HR: 1.23; P<.0001) in BRAF/MEK inhibitor-treated patients |
| Smit, 2005 [155] | NA | Elevated baseline LDH was a strong negative prognostic factor and an independent predictor of OS both before (P=.0002) and after treatment (P<.0001). |
| Tartour, 1996 [158] | 320 IU/L | High LDH was correlated significantly with **response rate (**4% vs 33%, P=.02); it was **not significant for OS (**P=.12**).** |
| Tas, 2005 [159] | NA | Elevated LDH (P=.01) was significantly correlated with reduced OS. |
| Wilgenhof, 2013 [163] | ULN | **Elevated baseline LDH** was **significantly associated with worse OS (**P=.017). |
| Boerlin, 2022 [57] | ULN | Elevated LDH levels were associated with worse OS only in univariate analysis. (P=.017) |
| Gassenmeier, 2021 [46] | 250 U/L | **Elevated LDH levels were not prognostic for DSSap (**HR: 1.48, P=.067**).** |
| Schouwenburg, 2018 [150] | Normal (<250 U/L), 1–2× ULN, ≥2× ULN | Elevated LDH (1–2× ULN: HR 1.6 (1.3–2.0) P<.0001; ≥2× ULN: HR 2.2 (1.8–2.8) P<.0001), poor ECOG PS, symptomatic brain metastases, and more than 3 metastatic organ sites independently predicted worse OS in vemurafenib-treated patients. Combining these into a **risk score** stratified patients effectively. |
| **Alkaline Phosphatase (ALP)** | | |
| Reinert, 2020 [83] | 130 U/L | ALP levels were not predictive for OS (P=.04). |
| **Aspartate Aminotransferase** | | |
| Cybluska-Stopa, 2012 [103] | NA | Elevated AspAT was an independent negative prognostic factor of OS (median OS: 5.1 vs. 7.5 month). |
| **Tumor-Associated Protein Biomarkers** | | |
| **S100B proteins** | | |
| B. Weide, 2012 [98] | NA | Elevated S100 was an independent prognostic factor in stage IV MM for OS (HR: 1.7 (95% CI: 1.4–2.1), P<.001) and distant metastatis. |
| Boerlin, 2022 [57] | ULN | Elevated S100 levels were associated with worse PFS (HR: 4.74; P<.0001). |
| Bolander, 2008 [99] | S100A1B + S100BB (150 ng/L),S100A1B (50 ng/L), S100BB (50 ng/L) | S100B-B was associated with increased risk of relapse (P=.016). All 3 markers (S100, S100-A1B, S100-BB) were associated with OS and DSS (P<.001). S100A4 was not prognostic (P=.96). |
| Braune, 2020 [58] | 0.105 mg/L | Elevated S100 levels were detected 46.4% of active disease cases. |
| Cao, 2007 [100] | 0.20 µg/L | Elevated S100 is prognostic for OS (P=.021) and DFS (P=.011). |
| Deckers, 2020 [104] | 0.15 mg/ml | Elevated S100 correlated with tumor burden, it was not prognostic for OS. |
| Domingo-Domènech, 2007 [106] | 0.15 µg/l | Elevated S100 levels were independent predictors of worse DFS and OS (HR: 3.31 (95% CI: 1.10–9.89), P=.032). Dynamic changes of S100 during therapy further stratified prognosis (OS: HR: 1.017 (95% CI: 1.008–1.026), P<.0001; DFS: HR: 1.029, P<.0001) |
| Egberts, 2008 [107] | 0.2 μg/dL | S100 (**OR=2.22 (P=.005))** was an independent prognostic factor predicting OS and is superior to LDH (median OS: 14.9 vs. 5.1 months). |
| Felix, 2016 [110] | 0.15 µg/L | Low (11.7 vs. 6.7 months (P=.011)) levels of S100 at baseline and week 3-6 correlated with better response and OS. |
| Foppen, 2016 [112] | 0.10 µg/L | Elevated S100 levels were associated with worsened OS (median OS 5.1 vs. 24.9 weeks (P=.03)). |
| Forschner, 2020 [113] | NA | There was no significant difference in between elevated S100 level and OS. |
| Gassenmeier, 2021 [46] | 0.1 μg/L | Elevated S100 levels were significantly associated with DSSap (HR: 2.17, P=.00062) in BRAFMEKi-treated patients. S100 were superior to LDH in predicting best response, and detecting disease progression earlier than imaging. |
| Kruijff, 2009 [126] | 0.15 µg/l | Elevated preoperative S100 was independently associated with worse DFS (HR: 2.6 (95% CI: 1.1–6.1), P=.03). |
| Meral, 2001 [139] | 0.08 μg/l | Elevated S100 levels predicted an unfavourable prognosis but it was not evaluable for survival impact. |
| Sanmamed, 2014 [145] | 0.1 µg/l | Elevated S100 at baseline correlated with tumor burden, but it was not predictive for PFS. At best response, elevated S100 levels were associated with significantly longer PFS (15 vs 3 months, P=.002). |
| Smit, 2005 [155] | 0.16 µg/l | Normal S100 at baseline was linked to better OS (median OS 14.0 vs. 6.6 months (P=.0002)), but it was not significant in multivariable analysis. **After treatment** S100B were **independently associated with OS.** |
| Tarhini, 2009 [157] | 0.15 µg/l | Elevated baseline S100 levels were associated with worse OS (HR: 1.39 (95% CI: 1.01–1.92), P=.043). Increasing S100 level during follow-up was associated with the worst RFS and OS (HR: 1.44 (95% CI: 1.06–1.95), P=.021). |
| **MIA (Melanoma Inhibitory Activity)** | | |
| Allen, 2011 [54] | 22 ng/dl | High baseline MIA levels were prognostic for OS (HR: 2.2 (95% CI: 1.0–4.8), P=.047). |
| Cao, 2007 [100] | 14 ng/ml | MIA levels **increased during disease progression** (P=.028), but it were not prognostic for OS and DFS. |
| Faries, 2004 [109] | 8.5 ng/ml | Elevated MIA at 4-6 months was a **strong independent predictor** of poor DFS (HR: 5.98 (95% CI: 3.25–10.99), P<.001) and OS (HR: 9.23 (95% CI: 4.78–17.84), P<.001; multivariate HR: 13.14 (95% CI: 5.36–32.20), P<.00). |
| Felix, 2016 [110] | 7.5 μg/L | Decreased MIA levels at week 3 were associated with better response (14.4 vs 19.0 μg/L in CR, PR, SD vs PD (P=.049)). MIA levels were not associated with OS. |
| Henry, 2013 [70] | 9.5 ng/ml | Elevated MIA levels were significantly associated with reduced OS, |
| Hofmann, 2011 [118] | 12 ng/ml | Elevated MIA levels were associated with high recurrence risk **(OR=5.2 (P<.0001)),** it also predicted relapse risk, but were not prognostic for OS. |
| Meral, 2001 [139] | 18.7 ng/mL | A decrease in MIA levels during treatment predicted better OS (HR: 3.67 (95% CI: 1.05–12.84), P=.04). |
| Sanmamed, 2014 [145] | 9 µg/l | High MIA at baseline was correlated with high tumor burden and partial (vs. complete) response (P=.026), but it was not predictive of PFS. Elevated MIA was associated with longer PFS (24 vs 4 months, P=.035) at 1-month follow-up. At best response MIA was linked to longer PFS (8 vs 4 months, P=.07), levels increased at progression (P=.002). |
| **serum CEACAM1 (Carcinoembryonic Antigen-Related Cell Adhesion Molecule 1)** | | |
| Sivan, 2012 [154] | increased/decreased | A decrease in serum CEACAM1 levels were associated with a better OS, but the result was not significant. |
| **TA90 Immune Complex** | | |
| Chung, 2002 [101] | positive:absorbance ≥ 0.410 OD at 405 nm | TA90 immune complex was the only independent predictor of DFS and OS (p= 0.0001) in sentinel-positive patients: OS 92% (negative) vs. 18% (positive). |
| **YKL-40 (Chitinase-3-like Protein 1 / CHI3L1)** | | |
| Schmidt, 2006 [148] | normal/elevated | YKL-40 is an independent prognostic biomarker for RFS (HR: 1.6 (1.1–2.5; P=.03)) and OS (HR: 1.8 (1.2–2.6; P=.002)) in stage I to II melanoma patients. |
| **P-proteasome** | | |
| Henry, 2010 [117] | 4300 ng/ml | High p-proteasome levels were associated with significantly worse PFS (HR: 7.34 (95% CI: 3.54–15.21), P<.0001) and OS (HR: 5.92 (95% CI: 2.84–12.33), P<.0001) in stage I-II melanoma patients. |
| Henry, 2013 [70] | 351 ng/ml | Elevated p-proteasome levels were significantly associated with reduced OS and independently predicted poorer PFS (HR: 2.97 (95% CI: 1.11–7.95), P=.030). |
| **Galectin-3** | | |
| Vereecken, 2009 [92] | 10 ng/ml | Elevated Galectin-3 levels were associated with worsened OS (HR: 4.64, P=.0001). |
| Wu, 2018 [166] | 7.088 ng/ml | Elevated pre-treatment serum Galectin-3 was associated with reduced OS and response rates. (HR: 4.8 (95% CI: 1.4–16.8), P=.01) |
| **Osteopontin (OPN)** | | |
| Filia, 2013 [111] | 103.14 ng/ml | High OPN levels were not associated with DSS or OS. OPN levels may reflect disease burden. |
| Prasmickaite, 2015 [141] | 25 ng/ml | OPN levels were higher in melanoma patients than controls but **not associated with OS (P=.87) or RFS (P=.75).** |
| **Circulating Melanoma Cell-Associated Biomarkers** | | |
| **Tyrosinase mRNA** | | |
| Schmidt, 2005 [87] | positive/negative | Positive tyrosinase-mRNA was associated with worsened OS (HR: 1.6, 95% CI: 1.1–2.6, P=.04). |
| **MART-1 mRNA** | | |
| Schmidt, 2005 [87] | positive/negative | MART-1 mRNA was not predictive for OS. |
| **Circulating Tumor Cells (CTCs)** | | |
| Fusi, 2009 [114] | Tyrosinase mRNA, Mart-1/Melan-A mRNA (4.4 × 10⁻⁵ ratio) | Serial detection of CTCs (HR 2.23 (95% CI: 1.40–3.55; P<.001)) was a moderate time-dependent prognostic marker for DMFS in stage III melanoma. |
| Hoshimoto, 2012 [119] | MART-1, MAGE-A3, GalNAc-T (≥ 2 positive vs. 0-1 positive) | Detection of ≥ 2 CTC biomarkers were associated with poor DM-DFS (HR: 2.13 (95% CI: 1.20–3.76), P=.009), MSS (HR: 1.88 (95% CI: 1.02–3.47), P=.043), and RFS (HR: 1.70 (95% CI: 1.01–2.87), P=.046). It was a prognostic factor for recurrence and survival in Stage III melanoma patients with SLN metastasis. |
| Khoja, 2013 [124] | < 2 positive, ≥ 2 negative/ 7.5 ml | Elevated CTC count were associated with worse OS (**HR:** 2.403 (95% CI: 1.303–4.430), P=.005). CTCs < 2 during treatment predicted better OS (**HR:** 0.34 (95% CI: 0.14–0.81), P=.015). Increased CTCs often predicted radiologic progression. |
| Koyanagi, 2010 [125] | 0, 1, 2 | Increase in CTCs on therapy was an independent predictor of poor PFS (HR: 12.6 (95% CI: 4.78–33.4), P<.0001) and OS (HR: 6.11 (95% CI: 2.37–15.7), P=.0005); the number of markers also correlated with poor prognosis. |
| Li, 2018 [130] | ≥ 6/7.5 ml baseline;  change after treatment | High baseline CTC count was associated with worse DSS (HR = 2.262 (95% CI: 1.199–4.267), P=.012). Increasing CTC count after treatment predicted worse DSS (HR: 2.913 (95% CI: 1.304–6.507), P=.009).Alteration of CTC count before and after treatment was an indicator of therapy response and prognosis. |
| Lucci, 2020 [134] | ≥ 1/ 7.5 ml | ≥1 CTC was independently associated with relapse at both 6 (HR: 3.13 (95% CI: 1.21–8.09), P=.018) and 54 months (HR: 2.25 (95% CI: 1.25–4.06), P=.006). |
| Lucci, 2023 [135] | ≥ 1/ 7.5 ml | CTCs were detected in 76% of patients before radiologic relapse; CTC positivity was significantly associated with worse RFS (HR: 3.36 (95% CI: 2.25–5.04). |
| RAO, 2010 [142] | ≥ 2 / 7.5 ml | Patients with ≥2 CTCs had significantly worse OS (HR: 3.2 (95% CI: 1.6–6.5), P=.001). |
| S. Hall, 2018 [144] | NA | ≥1 CTC at baseline was independently predicted PFS within 180 days (HR: 4.69 (95% CI: 1.59–13.77), P=.005). |
| **Circulating Nucleic Acid Biomarkers** | | |
| **cfDNA** | | |
| Gonzalez-Cao, 2018 [68] | cfDNA_BRAFV600_ quantity: 10.5 pg/μL  Early dynamics of cfDNA_BRAFV600_ (Clearance vs persistence after therapy) | Elevated cfDNA levels were correlated with worse OS (HR:0.25, P=.0004) and PFS (HR: 0.39, P=.009). Dynamics of early cfDNA changes were a strong, early predictor of progression (Sensitivity 73%, specificity 100%) and survival (OS: 5.3 vs 21.9 months, p < .0001). |
| Lin, 2018 [132] | SNV load (2) | Higher SNV load was independently associated with poor OS and DFS in multivariable analysis |
| Lin, 2018 [132] | CNV amplification (EGFR/MET) | cfDNA ampification indicated residual disease and had potential impact on OS. |
| Lin, 2018[132] | VAF levels | VAF levels increased after relapse, correlating with tumor burden and progression (P=.019) |
| S. Valpione, 2018 [86] | 89 pg/ml | cfDNA (total and per tumor volume) was superior to LDH as a prognostic marker; high levels independently associated with worse OS (HR = 2.22 (95% CI: 1.18–5.55), P=.004). |
| Sanmamed, 2015 [146] | BRAFV600E 216 copies/mL | Low baseline levels correlated ith longer OS (22.7 vs 8.6 m P = .001) and PFS (9 vs 3 m P = .024) |
| Silva, 2018 [153] | CNAS score (>75th percentile) | Mortality was higher with detectable cfDNA and CNAS. (HR: 3.4 (95% CI: 1.5–7.9), P=.005) and elevated cfDNA (HR 2.3 (95% CI: 1.1–4.8), P=.032). CNAS can **distinguish high-risk resected melanoma** and predict survival independently of stage and cfDNA concentration. |
| **cfDNA/tumour volume ratio** (shedding coefficient) | | |
| S. Valpione, 2018 [86] | **8 pg/(mL*cm³)** | Patients with high shedding coefficient had worse OS (HR 2.7 (P=.029)). |
| Tawbi HA, 2022 [160] | shedding (detectable vs undetectable) | Detectable ctDNA (HR: 0.75 (95% CI: 0.58–0.96)) was associated with better PFS. |
| **ctDNA** | | |
| Aoude, 2023 [55] | decrease/increase (after treatment initiation, 12 months) | Decreasing ctDNA levels correlated with tumor shrinkage and treatment response. Increasing ctDNA levels correlated with radiological disease progression. |
| Braune, 2020 [58] | detectable/undetectable | Detectable ctDNA post-surgery predicted shorter PFS (P=.018). ctDNA dynamics within 30 days of treatment predicted RECIST response in 19/20 patients. |
| Forschner, 2020 [113] | detecable/undetectable | Detectable ctDNA (P=.08) at baseline was associated with worse OS. Rising ctDNA levels often indicated progressive disease before imaging |
| Forthun, 2019 [67] | dynamics (1%) | ctDNA dynamics (>1%) were correlated with radiological progression, response, and relapse, and shortened PFS (HR: 6.76; P=.00) and OS (HR: 6.78, P=.002). |
| Keller, 2019 [123] | Increase vs. Decrease (early change ≥ 20% within ~26 days) | Early ctDNA increase was associated with early progression and shorter PFS (median 21 days vs. 145 days; P=.001), and decreased ctDNA was associated with response. |
| Kozak, 2019 [47] | detectable/undetectable  (4–8 weeks after treatment) | Patients with **undetectable cfDNA after 1–2 months of therapy** had **longer PFS and OS.** |
| Lee, 2017 [18] | detectable/undetectable (week 12) | Early ctDNA clearance predicted response (Sensitivity: 79%, Specificity: 94%, Positive Predictive Value: 98%, Negative Predictive Value: 59%). |
| Lee, 2018 [76] | detectable/undetectable  (median: 8.3 weeks) | Postoperative ctDNA detectability was a strong and independent predictor of early relapse, distant metastasis, and OS. |
| Lin, 2018 [132] | SNV burden | Higher SNV burden was associated with poor OS |
| Seremet, 2019 [30] | detectable/undetectable (serial measurements, every 3 weeks) | Baseline and dynamic ctDNA levels were independently predicted objective response (lower ctDNA at responders (CR/PR): median: 0 copies/mL vs. 31 copies/mL in non-responders; mean: 43 vs. 1199 copies/mL (P=.0052)). Early clearance of ctDNA (week 3–9) was associated with prolonged PFS and OS. |
| Tan, 2019 [156] | ≥1 copy/mL at baseline;  ≥1 mutant/mL postoperative | **Detectable ctDNA levels predicted RFS (HR: 2.9, P=.002; 90% vs 49% relapsed; median RFS: 6.2 vs 10.8 months) and DMFS (HR: 2.9, P=.003; median DMFS: 7.4 vs 10.8 months) before surgery.**  **Detectable ctDNA levels predicted RFS (HR: 10, P<.001; 100% vs 41% relapsed, median RFS: 3.5 vs 13.5 months) and DMFS (HR: 11, P<.001) postoperatively. (In multivariate: RFS HR: 11, P<.001; DMFS HR: 9, p< 0.01)).** |
| **miRNAs** | | |
| Levati, 2022 [128] | miR-2045p | Decreased baseline miR-2045p level associated with poorer PFS (HR: 2.33 (95% CI: 1.13–4.80), P=.02) and OS (HR: 2.54 (95% CI: 1.25–5.13), P=.01). |
| Levati, 2022 [128] | miR-485-3p (0.013) | **Decreased miR-485-3p** and elevated **miR-1246/miR-485-3p ratio** were **independent predictors of PFS.** |
| Levati, 2022 [128] | miR-1246 (8.637) | MiR-1246 level did **not remain significant** in multivariate analysis. MiR-1246/miR-485-3p ratio showed a trend toward significance (HR: 2.28, P=.082) for OS. |
| Li, 2014 [129] | miR221 expression (high/low) | High serum miR-221 was independently associated with worse 5-year OS (22.1% vs. 54.6%) and DFS (12.5% vs. 45.2%); correlated with tumor thickness and metastasis. (OS: HR = 3.19 (95% CI: 1.78–6.78), P=.007; DFS: HR = 2.12 (95% CI: 1.96–8.55), P=.01) |
| Ruggiero, 2022 [50] | miR-579-3p high/low | High miR-579-3p was associated with better PFS (HR < 1, P<.05). |
| Ruggiero, 2022 [50] | miR-488 high/low | High miR-488 was associated with worse PFS (HR > 1, P<.05). |
| **Immune Cells, Hematologic Biomarkers/ Cell-based hematologic biomarker** | | |
| **ALC** | | |
| Aamdal, 2022 [1] | 1.1 × 10⁹/L | ALC was not associated with OS (HR: 1.10 (95% CI: 0.72–1.70), P=.661) |
| Awada, 2021 [56] | 750/mm³ | Decreased ALC was correlated with worse PFS and OS. |
| Wilgenhof, 2013 [163] | 800/mm³ at week 6 | ALC was useful for early OS prediction (P=.01). |
| Zaragoza, 2016 [38] | NA | ALC was not an independent predictor of OS (P=.20). |
| **ANC** | | |
| Aamdal, 2021 [1] | 7.3 × 10⁹/L | ANC were not associated with OS (HR: 1.34 (95% CI: 0.78–2.30), P=.293). |
| Zaragoza, 2016 [38] | 7.5 × 10⁹/L | Elevated ANC was significantly associated with worse OS (HR: 3.28; P=.007). |
| **Leukocyte count** | | |
| Ivanainen, 2019 [121] | NA | Leukocyte count were not predictive for OS and PFS. |
| **Lymphocyte count** | | |
| Guida, 2003 [115] | NA | **Low absolute lymphocyte count** (P=.0049) and low lymphocyte percentage (P<.0001) were associated with worse OS, but it were not significant in multivariate analysis. |
| Yu, 2017 [93] | 1.8×10⁹/L | Lymphocyte count were not significantly associated with OS and RFS independently. |
| **Eosinophils** | | |
| Guida, 2003 [115] | NA | Eosinophils were not prognostic for OS. |
| Rosner, 2018 [27] | 1.1% | Higher relative eosinophil levels were associated with improved OS (HR: 2.38, P=.007). |
| **Absolute monocyte count** | | |
| Rosner, 2018 [27] | 0.8 × 10⁹/L | Higher levels of monocytes were associated with worse OS (HR: 2.75, P=.01). |
| Schmidt, 2005a [28] | ULN | Monocyte levels were significantly predicted OS. |
| **Relative Basophils** | | |
| Rosner, 2018 [27] | 0.6% | Higher levels of relative basophils were trended toward to improved OS (HR:1.85, P=.08). |
| **WBC** | | |
| Aamdal, 2021[1] | 10 × 10⁹/L | Elevated WBC were associated with OS (HR: 1.89, p = .010 (univariate)). |
| **Neutrophils** | | |
| Schmidt, 2005a [28] | ULN | Elevated neutrophil count was associated with worsened OS (HR: 1.4, P=.02) |
| Yu, 2017 [93] | 4×10⁹/L | Elevated neutrophil count trended toward worsened OS and RFS. |
| **Immune Cell Phenotype Markers (Flow Cytometry)** | | |
| **T-cell subsets** | | |
| Machiraju, 2023 [20] | Frequencies analyzed (NA) | Young patients (≤40 years) had **lower CD45RA - CD27 - effector memory CD8^+^ T-cell frequencies** at baseline, which was **linked to worse prognosis and PFS.** |
| Tawbi HA, 2022 [160] | CD4^+^/CD8^+^ T-cell ratio | High CD4^+^/CD8^+^ ratio (HR: 0.58 (95% CI: 0.40–0.84) was associated with better PFS. |
| **Inflammatory and Ratio Biomarkers** | | |
| **CRP** | | |
| Boerlin, 2022 [57] | ULN | Elevated CRP levels were associated with worse OS (HR: 4.36; P=.013) and PFS (HR: 3.27; P=.003). |
| Fang, 2016 [108] | continous variable | Elevated CRP levels were associated with poorer OS (HR 1.17 (1.07–1.27)), MSS, and DFS. |
| Guida, 2003 [115] | 1.5 mg/gl | Lower CRP levels before treatment were associated with better OS. |
| Ivanainen, 2019 [121] | 9.5 mg/ml | Elevated CRP predicted poor PFS (2.0 vs. 7.0 months (P=.005)) and OS (10.0 vs. not reached, (P=.000004)) in anti-PD-1-treated patients. |
| Schildbach, 2023 [147] | 3 mg/L | Elevated CRP levels were associated with shortened RFS (HR: 3.36, P<.001) |
| Schneider, 2021 [149] | 10 mg/dl | Elevated levels of CRP were associated with poor OS in melanoma patients with BM. |
| Tartour, 1996 [158] | 10 mg/L | Elevated CRP level independently predicted poor IL-2 response (response rates: low CRP 40% vs. high CRP 8% (P=.001)) and worse OS (P<.0001). |
| Wilgenhof, 2013 [163] | 5 x ULN | CRP was a strong negative prognostic marker for OS (HR 0.08 (95% CI: 0.03–0.23). |
| **NLR** | | |
| Andressa, 2020 [4] | Continuous variable | Elevated NLR correlated significantly with worse OS in CheckMate-066 and -067 several arms (HR ~1.06 - 1.13). |
| Hu, 2023 [120] | 3 | Elevated NLR was an independent prognostic factor for OS. |
| Ivanainen, 2019 [121] | 2.65 | **High NLR** was associated with **shorter** PFS (2.0 vs. 7.0 months, P=.02)), and OS (7.0 vs. 19.0 months, (P=.009)), but NLR (>5) was not an independent predictive factor for PFS (P=.21) or OS (P=.056) in multivariate analysis. |
| Schildbach, 2023 | 3.5, continuous variable | High NLR **was a significant independent predictor** of shorter RFS. (HR 1.34, 95% CI: 1.05–1.71, P=.019), but it **was not significant** when included in the multivariate model for OS. |
| Schneider, 2021 [149] | 4 | Elevated NLR was associated with poor OS (35 vs. 6 months, P=.004)) in melanoma patients with BM. |
| **dNLR** | | |
| Schildbach, 2023 | 2 | Elevated dNLR was a signicant independent predictor for OS (HR 2.43, 95% CI: 1.19–4.97, P=.015), but it **was not significant** when included in the multivariate model for OS. |
| **PLR** | | |
| Kopecky, 2022 [74] | 160 | High PLR was associated with worsened OS (HR: 3.27, P<.001 (uni)) and PFS (HR: 2.08, P=.05 (multi)). |
| Schneider, 2021 [149] | 145 | Elevated PLR was associated with poor OS (35 vs. 6 months (P=.033)) in melanoma patients with BM. |
| Yu, 2017 [93] | 129 | Elevated PLR was associated with shortened OS (P=.01) and RFS (P=.002). |
| **LMR** | | |
| Kopecky, 2020 [74] | 2 | Low LMR was associated with worsened OS (HR: 0.31, p 0.01) and PFS (HR: 0.38, p = .03 (multi)). |
| Schildbach, 2023 [147] | 3.5 | Low LMR was associated with shortened RFS (HR: 2.20, P=.003). |
| Schneider, 2021 [149] | 2 | Low LMR was associated with poor OS (25 vs. 5 months (P=.006)) in melanoma petients with BM.. |
| **ESR** | | |
| Guida, 2003 [115] | NA | High ESR was significantly associated with worse OS (P=.022), but it was not significant in multivariate analysis. |
| **Fibrinogen** |  |  |
| Guida, 2003 [115] | 450 mg/dL | High fibrinogen levels were independently predicted poor OS (HR 1.99 (95% CI: 1.24–3.19, P=.004)). |
| Guida, 2006 [116] | normal/high | High fibrinogen levels had an independent impact on OS and were one of the stronger factors (HR 3.31 (95% CI: 1.54–7.13), P=.002). |
| **Soluble Cytokines and Immune Factors** | | |
| **Interleukin-4 (IL-4)** | | |
| A. Porter, 2001 [96] | 7.8 pg/ml | IL-4 were not prognostic for OS. |
| **Interleukin-6 (IL-6)** |  |  |
| A. Porter, 2001 [96] | NA | IL-6 levels were not prognostic for OS. |
| Damuzzo, 2016 [8] | NA | Elevated IL-6 levels independently predicted worsened OS (HR: 4.76, 95% CI: 1.35–16.76, P=.015). |
| Guida, 2006 [116] | terciles, NA | Elevated IL-6 levels were associated with worse OS (P=.005). |
| Tartour, 1996 [158] | 20 pg/mL | Elevated IL-6 correlated significantly with response rate (P=.007) and was **not significant for OS after adjusting.** |
| Tas, 2005 [159] | median: 30.55 pg/mL | Elevated IL-6 levels were correlated with reduced OS (P=.002). |
| **Interleukin-8 (IL-8)** |  |  |
| Guida, 2006 [116] | (low: 0-3, intermediate: 4-20, high: 21 pg/ml)) | IL-8 levels were not able to discriminate between patients with different survival. |
| M.F.Sanmamed, 2017 [137] | 9.2% increase vs. ≤9.2% change after 2–3 weeks of therapy | Early IL-8 decrease was associated with better response and longer OS (HR: 3.7 (95% CI: 1.5–9.5), P=.003) during anti-PD-1 therapy. Increasing IL-8 level predicted poor response; |
| Ugurel, 2001 [161] | 226.8 pg/ml | IL-8 was an independent prognostic marker for poor OS (P<.00005) and PFS (P<.00005). |
| **Interleukin-10 (IL-10)** | | |
| A. Porter, 2001 [96] | NA | IL-10 levels were not prognostic |
| Guida, 2006 [116] | NA | IL-10 levels were undetectable in the majority of patients, the results are not reported. |
| **Intgerleukin-12** | | |
| Guida, 2006 [116] | (low: 0-54, intermediate: 55-114, high: 115 mg/ml) | High IL-12 (P=.01) levels were associated with worse OS. IL-12 levels progressively rose during treatment in long-surviving patients. |
| **Interleukin-18** | | |
| Musaelyan, 2022 [22] | 273 pg/ml | High IL-18 levels trended toward shorter PFS, but it was not significant. |
| **IFN-γ** | | |
| A. Porter, 2001 [96] | NA | IFN-γ detectable levels independently predicted recurrence. |
| **IFN-β** | | |
| Pedersen, 2020 [23] | median split | High IFN-β levels trended toward longer PFS (HR: 0.22, P=.063). |
| **TNF-α** | | |
| A. Porter, 2001 [96] | 15.6 pg/ml | TNF-α levels were not prognostic for OS. |
| Pedersen, 2020 [23] | median split | Elevated TNF-α was associated with longer PFS (HR: 0.081, P=.019). |
| Tas, 2005 [159] | median 64.95 mIU/mL | TNF-α levels had no prognostic significance for OS in stage IV melanoma. |
| **MCP1 (CCL2)** | | |
| Pedersen, 2020 [23] | median split | High MCP1 was associated with longer PFS (HR: 0.073, P=.016). |
| **serum Interleukin-2 Receptor (sIL-2R)** | | |
| Guida, 2006 [116] | (low: 442, intermediate: 443-1027, high: 1028 U/ml)) | High sIL-2R (P=.01) levels were independently associated with worse OS. sIL-2R (HR 2.72 (95% CI: 1.03–7.17, P=.043)). sIL-2R levels progressively rose during treatment in long-surviving patients. |
| **Soluble immune checkpoint proteins** | | |
| Lu, 2023 [133] | sPD1/PDL-1 ratio (median:.67 pg/ml) | Baseline high PD-1/PD-L1 ratio associated with superior OS (median 118.9 vs. 41.4 weeks; log-rank P=.003). High change of soluble PD-L1 significantly increased mortality (HR: 1.31, 95% CI: 1.11–1.58). High change in PD-1/PD-L1 ratio reduced mortality risk (HR: 0.70, 95% CI: 0.55–0.84). |
| Machiraju, 2023 [20] | sPDL1, sTIM3 | Elevated sPDL1 (P=.05) and sTIM3 (P=.054) were associated with shortened PFS in young patients (≤40 years), especially those experiencing disease progression within 6 months. |
| **β2-microglobulin** | | |
| Cao, 2007 [100] | 2.5 mg/l | Elevated β2-MG was not prognostic for **DFS or OS**, it was increased at **disease progression** (P=.030). |
| Musaelyan, 2022 [22] | 12 nmol/l | Elevated β2-MG levels were associated with shorter PFS (HR: 0.09, P=.008). |
| **serum MICA** | | |
| Felix, 2016 [110] | 45 μg/L (30th percentile) | serum MICA levels were not associated with OS. Lower sMICA levels at baseline were associated with increased risk of developing immune-related adverse events (P=.049), |
| Koguchi, 2015 [16] | ≥247 pg/mL (80th percentile) | Elevated sMICA levels were associated with worsened OS (HR: 1.75, P=.042 (phase III)). |
| **Anti-MICA antibodies** | | |
| Felix, 2016 [110] | Fluorescence Intensity: 2 | It was not significantly associated neither with response or OS. |
| **Angiogenic and Growth Factors** | | |
| **Vascular Endothelial Growth Factor (VEGF)** | | |
| Corrie, 2018 [102] | NA | Neither baseline plasma nor serum VEGF were prognostic for OS |
| Decoster, 2005 [105] | mean 186.3 ng/L | No correlation between survival and baseline VEGF levels was observed. |
| Koguchi, 2015 [16] | ≥157 pg/mL | Elevated VEGF levels were associated with poor OS. |
| Mahalingam, 2014 [138] | 300 pg/ml | Low VEGF was associated with longer TTP (50 vs. 15 weeks; P=.02). |
| Ugurel, 2001 [161] | 363.8 pg/mL | VEGF was an independent prognostic marker for poor OS (P=.001) and PFS (P=.0036). |
| **Vascular Endothelial Growth Factor Receptor 1 (VEGFR1)** | | |
| Corrie, 2018 [102] | NA | Neither baseline plasma nor serum VEGFR1 was prognostic for OS. |
| Decoster, 2005 [105] | mean:101.4 ng/L | High VEGFR1 levels were associated with a worse OS, although did not reach significance. |
| Mahalingam, 2014 [138] | median (NA) | Elevated VEGFR1 levels showed trends, but it was not significant. |
| **Vascular Endothelial Growth Factor Receptor 2(VEGFR2)** | | |
| Decoster, 2005 [105] | mean:8920.6 ng/L | No correlation between survival and baseline VEGFR2 levels were observed. |
| Mahalingam, 2014 [138] | median based (high/low) | Elevated VEGFR2 levels showed trends, but it was not significant. |
| **Placental Growth Factor (PIGF)** | | |
| Decoster, 2005 [105] | mean:46.5 ng/L | High PlGF levels were associated with a worse OS, although did not reach significance. |
| **Hepatocyte Growth Factor (HGF)** | | |
| Hügel, 2016 [71] | 1602.4 pg/mL | Elevated HGF levels were associated with worse OS (HR: 1.27, P=.024) and PFS (HR: 1.39, P=.001). Increasing levels at median 84 days trended toward association with disease progression (P=.073). |
| Lu, 2018 [49] | 438 pg/mL | Elevated HGF levels were associated with worse OS (HR 1.24, P=.045). HGF at progression was not predictive of postprogression survival. |
| **Angiopoietin-2 (ANGPT2)** | | |
| Wu, 2017 [165] | 3175 pg/ml and ≥1.25 fold change | High baseline ANGPT2 associated with worse OS (HR 2.48; 95% CI: 1.5–4.1; P=.0003). Early ANGPT2 increase were correlated with OS (HR ~2) and response rates in patients receiving immune ICI. Large ANGPT2 fold changes were associated with shortened OS. |
| **basic Fibroblast Growth Factor (bFGF)** | | |
| Ugurel, 2001 [161] | 3.19 pg/ml | bFGF was an independent prognostic marker for poor OS (P<.00005) and PFS (P<.00005). |
| **Angiogenin** | | |
| Ugurel, 2001 [161] | 407.1 ng/ml | Angiogenin levels were not prognostic for OS or PFS. |
| **Metabolic and Nutritional Biomarkers** | | |
| **Albumin** | | |
| Seethamaru, 2012 [151] | 3.5-50 g/dl | Low albumin levels were associated with **worse survival (**median OS: 14.9 vs. 9.3 months (P=.001). |
| Krakowski, 2021 [17] | 25 g/l | Low albumin levels were associated with worse PFS (HR: 1.06, P=.011) and OS (HR: 1.11, P<.001) in ICI treated patients, and it was a predictor of response to MAPKis. |
| **D-dimer** | | |
| Desch, 2016[64] | 0.6 mg/L | Elevated D-dimer levels were significantly associated with PFS (HR: 2.47, P=.012) in stage III and worse OS in stage IV melanoma patients. Dynamic increase in D-dimer levels may aid in monitoring disease progression. |
| **Free T3** | | |
| Zhao, 2022 [39] | 4.535 pmol/L | High free T3 levels were associated with better OS (HR: 0.554 (0.355–0.865), P=.009). |
| **Vitamin D** | | |
| Fang, 2016 [108] | (<16, <20, <30 ng/mL) | Lower vitamin D levels independently predicted poorer OS (HR: 1.85), MSS (HR: 1.52), and DFS (HR 2.00), even after adjusting for CRP and other covariates. |
| **5-S-cysteinyl-dopa** |  |  |
| Umemura, 2017 [162] | 15 nmol/l | 5-S-CD was a better marker than LDH for prognosis and relapse detection in follow-up. Elevated levels at diagnosis predicted poor OS (P=.015). |
| **Thyroid Peroxidase Antibodies** | | |
| Zhao, 2022 [39] | 13.09 U/ml | High A-TPO levels were associated with better OS (HR: 0.567 (0.381–0.904), P=.0016). |
| **Erythropoeitn (EPO)** | | |
| Tas, 2005 [159] | median 31.85 mIU/mL), | EPO levels had no prognostic significance for OS in stage IV melanoma. |
| **Other Soluble Protein Markers** | | |
| **HSP90α** |  |  |
| Zhang, 2021 [95] | 82 ng/ml | High HSP90α levels were associated with shorter PFS (8.0 vs. 3.5 months; P=.096 (not significant overall); P=.008 (significant in non-immunotherapy subgroup)). Decreasing **HSP90α correlated with treatment response**, while an increase was associated with progression. |

Abbreviations: AJCC: American Joint Committee on Cancer; ALB: albumin; ALC: absolute lymphocyte count; ALP: alkaline phosphatase; ANC: absolute neutrophil count; ANGPT2: angiopoietin-2; anti-TPO: anti-thyroid peroxidase antibodies; BM: brain metastasis; BRAFi: BRAF inhibitor; BRAFV600E: BRAF V600E mutation; CEACAM1: carcinoembryonic antigen-related cell adhesion molecule 1; CCL2: C-C motif chemokine ligand 2; CD: cluster of differentiation; CD45RA: cluster of differentiation 45 isoform RA; CHI3L1: chitinase-3-like protein 1; CI: confidence interval; CNAS: copy number alteration score; CNV: copy number variation; CR: complete response; CRP: C-reactive protein; CTC: circulating tumor cell; ddPCR: droplet digital PCR; DFS: disease-free survival; DFI: disease-free interval; DM-DFS: distant metastasis disease-free survival; DMFI: distant metastasis-free interval; DMFS: distant metastasis-free survival; DSS: disease-specific survival; dNLR: derived neutrophil-to-lymphocyte ratio; ECOG: Eastern Cooperative Oncology Group; EGFR: epidermal growth factor receptor; EPO: erythropoietin; Gal-3: galectin-3; HGF: hepatocyte growth factor; HR: hazard ratio; HSP90α: heat shock protein 90 alpha; ICI: immune checkpoint inhibitor; IFN: interferon; IFN β: interferon beta; IL-6: interleukin-6; IL-18: interleukin-18; LDH: lactate dehydrogenase; ULN: upper limit of normal ratio; Log: logarithm; LMR: lymphocyte-to-monocyte ratio; MART-1: melanoma antigen recognized by T cells 1; MAGE-A3: melanoma antigen gene-A3; MAPK: mitogen-activated protein kinase; MCP-1: monocyte chemoattractant protein-1; MCP1: monocyte chemoattractant protein-1; MEK: mitogen-activated protein kinase kinase; MEKi: MEK inhibitor; MHC: major histocompatibility complex; MIA: melanoma inhibitory activity; miR: microRNA; MICA: MHC class I polypeptide-related sequence A; MM: malignant melanoma; MSS: melanoma-specific survival; NA: not available; Neu: neutrophils; NL: Netherlands; NLR: neutrophil-to-lymphocyte ratio; NPT: neopterin; OPN: osteopontin; OR: odds ratio; OS: overall survival; PD-1: programmed cell death protein 1; PD-1-: programmed cell death protein 1-negative; PD-L1: programmed death-ligand 1; PDL-1: programmed death-ligand 1; PEG-Intron: pegylated interferon alfa-2b; PFS: progression-free survival; PlGF: placental growth factor; PIGF: placental growth factor; PR: partial response; PS: performance status; RECIST: Response Evaluation Criteria in Solid Tumors; RFS: recurrence-free survival; RT-PCR: reverse transcriptase polymerase chain reaction; RT-qPCR: quantitative reverse transcriptase PCR; SD: standard deviation; SE: standard error; S100: S100 calcium-binding protein; SLN: sentinel lymph node; SNV: single nucleotide variant; SII: systemic immune-inflammation index; SRS: stereotactic radiosurgery; TA90: tumor-associated antigen 90; TG: thyroglobulin; TKI: tyrosine kinase inhibitor; TK activity: thymidine kinase activity; TNF-α: tumor necrosis factor-alpha; TPO: thyroid peroxidase; TTP: time to progression; UK: United Kingdom; ULN: upper limit of normal; UNL: upper normal limit; VAF: variant allele frequency; VEGF: vascular endothelial growth factor; VEGFR1: VEGF receptor 1; VEGFR2: VEGF receptor 2; WBRT: whole-brain radiotherapy; WBC: white blood cell count

# **Overall Survival: Univariate analyses**

## **Supplementary Figure S1:** Forest plot of univariate Hazard Ratios (HR) of overall survival (OS) and baseline serum biomarker level associations in melanoma patients, regardless of therapeutic agent.

**Abbreviations:** ALC: absolute lymphocyte count; ALP: alkaline phosphatase; ANC: absolute neutrophil count; A-TPO: anti-thyroid peroxidase antibody; CD37 activity: cluster of differentiation 37 activity; cfDNA: cell-free DNA; cHGF: circulating hepatocyte growth factor; CI: confidence interval; CRP: C-reactive protein; CTC: circulating tumor cells; ctDNA: circulating tumor DNA; dNLR: derived neutrophil-to-lymphocyte ratio; FT3: free triiodothyronine; FT4: free thyroxine; Gal-3: galectin-3; HGF: hepatocyte growth factor; HR: hazard ratio; IL-6: interleukin-6; IL-8: interleukin-8; Leu: leukocytes; LDH: lactate dehydrogenase; LMR: lymphocyte-to-monocyte ratio; Ly: lymphocytes; MART1-mRNA: melanoma antigen recognized by T cells 1 messenger RNA; MIA: melanoma inhibitory activity; miR: microRNA; mono: monocytes; Neu: neutrophils; NLR: neutrophil-to-lymphocyte ratio; PLR: platelet-to-lymphocyte ratio; S-100: S100 calcium-binding protein; SII index: systemic immune-inflammation index; TG: thyroglobulin; TSH: thyroid-stimulating hormone; VEGF: vascular endothelial growth factor; WBC: white blood cell count

## **Supplementary Table S2:** Pairwise comparisons of prognostic associations between serum biomarkers for OS.

| Group1 | Group2 | k1 | k2 | Ratio [CI_lower_; CI_upper_] | se | z | p |
| --- | --- | --- | --- | --- | --- | --- | --- |
| CRP | ctDNA | 11 | 15 | 0.7934 [0.5427; 1.1599] | 0.1938 | -1.1946 | 0.2322 |
| CRP | dNLR | 11 | 3 | 0.8660 [0.4569; 1.6412] | 0.3262 | -0.4412 | 0.6591 |
| CRP | IL-6 | 11 | 7 | 0.6421 [0.4170; 0.9888] | 0.2203 | -2.0110 | 0.0443 |
| CRP | LDH | 11 | 44 | 0.8803 [0.6496;1.1929] | 0.1551 | -0.8223 | 0.4109 |
| CRP | Neu | 11 | 3 | 1.0330 [0.5506; 1.9379] | 0.3210 | 0.1010 | 0.9195 |
| CRP | NLR | 11 | 11 | 0.8257 [0.5500; 1.2394] | 0.2072 | -0.9242 | 0.3554 |
| CRP | S100 | 11 | 8 | 0.8440 [0.5384; 1.3231] | 0.2294 | -0.7394 | 0.4597 |
| CRP | VEGF | 11 | 3 | 1.1928 [0.6654; 2.1380] | 0.2978 | 0.5920 | 0.5539 |
| ctDNA | dNLR | 15 | 3 | 1.0915 [0.5761; 2.0680] | 0.3260 | 0.2686 | 0.7883 |
| ctDNA | IL-6 | 15 | 7 | 0.8094 [0.5258; 1.2459] | 0.2201 | -0.9611 | 0.3365 |
| ctDNA | LDH | 15 | 44 | 1.1096 [0.8192; 1.5028] | 0.1548 | 0.6716 | 0.5018 |
| ctDNA | Neu | 15 | 3 | 1.3020 [0.6942; 2.4419] | 0.3209 | 0.8224 | 0.4109 |
| ctDNA | NLR | 15 | 11 | 1.0407 [0.6936;1.5616] | 0.2070 | 0.1928 | 0.8471 |
| ctDNA | S100 | 15 | 8 | 1.0638 [0.6789;1.6670] | 0.2292 | 0.2700 | 0.7871 |
| ctDNA | VEGF | 15 | 3 | 1.5034 [0.8389;2.6941] | 0.2976 | 1.3699 | 0.1707 |
| dNLR | IL-6 | 3 | 7 | 0.7415 [0.3790;1.4509] | 0.3425 | -0.8733 | 0.3825 |
| dNLR | LDH | 3 | 44 | 1.0165 [0.5595;1.8469] | 0.3046 | 0.0538 | 0.9571 |
| dNLR | Neu | 3 | 3 | 1.1928 [0.5294;2.6878] | 0.4145 | 0.4254 | 0.6706 |
| dNLR | NLR | 3 | 11 | 0.9535 [0.4952;1.8358] | 0.3342 | -0.1426 | 0.8866 |
| dNLR | S100 | 3 | 8 | 0.9746 [0.4924;1.9293] | 0.3484 | -0.0737 | 0.9412 |
| dNLR | VEGF | 3 | 3 | 1.3773 [0.6329;2.9976] | 0.3968 | 0.8069 | 0.4197 |
| IL-6 | LDH | 7 | 44 | 1.3709 [0.9504;1.9774] | 0.1869 | 1.6878 | 0.0914 |
| IL-6 | Neu | 7 | 3 | 1.6087 [0.8301;3.1174] | 0.3375 | 1.4084 | 0.1590 |
| IL-6 | NLR | 7 | 11 | 1.2859 [0.8160;2.0263] | 0.2320 | 1.0835 | 0.2786 |
| IL-6 | S100 | 7 | 8 | 1.3144 [0.8021;2.1539] | 0.2520 | 1.0850 | 0.2779 |
| IL-6 | VEGF | 7 | 3 | 1.8575 [1.0008;3.4475] | 0.3155 | 1.9625 | 0.0497 |
| LDH | Neu | 44 | 3 | 1.1734 [0.6529;2.1089] | 0.2991 | 0.5347 | 0.5929 |
| LDH | NLR | 44 | 11 | 0.9380 [0.6704;1.3124] | 0.1714 | -0.3737 | 0.7086 |
| LDH | S100 | 44 | 8 | 0.9588 [0.6510;1.4121] | 0.1975 | -0.2130 | 0.8313 |
| LDH | VEGF | 44 | 3 | 1.3549 [0.7919;2.3182] | 0.2740 | 1.1086 | 0.2676 |
| Neu | NLR | 3 | 11 | 0.7993 [0.4193;1.5239] | 0.3292 | -0.6804 | 0.4963 |
| Neu | S100 | 3 | 8 | 0.8171 [0.4167;1.6021] | 0.3435 | -0.5880 | 0.5565 |
| Neu | VEGF | 3 | 3 | 1.1547 [0.5350;2.4923] | 0.3925 | 0.3664 | 0.7141 |
| NLR | S100 | 11 | 8 | 1.0222 [0.6378;1.6384] | 0.2407 | 0.0913 | 0.9273 |
| NLR | VEGF | 11 | 3 | 1.4446 [0.7921;2.6345] | 0.3066 | 1.1997 | 0.2302 |
| S100 | VEGF | 8 | 3 | 1.4132 [0.7519;2.6560] | 0.3219 | 1.0742 | 0.2827 |

Note: Pairwise comparisons were performed using meta-regression–based indirect analyses. Relative effects represent differences in the strength of prognostic associations between biomarkers.
Abbreviations: CRP: C-reactive protein; ctDNA: circulating tumor DNA; dNLR: derived neutrophil-to-lymphocyte ratio; IL-6: interleukin-6; LDH: lactate dehydrogenase; Neu: neutrophil count; NLR: neutrophil-to-lymphocyte ratio; S100B: S100B protein; VEGF: vascular endothelial growth factor; CI: confidence interval; SE: standard error.

## **Supplementary Figure S2:** Forest plot of univariate Hazard Ratios (HR) of verall survival (OS) and baseline serum biomarker level associations in melanoma patients subgrouped based on therapeutic agent.

**Abbreviations:** ALC: absolute lymphocyte count; ALP: alkaline phosphatase; ANC: absolute neutrophil count; A-TPO: anti-thyroid peroxidase antibody; BRAF–MEK inhibitors: BRAF and MEK kinase inhibitors; cHGF: circulating hepatocyte growth factor; CI: confidence interval; CRP: C-reactive protein; CTC: circulating tumor cell; CTII: circulating type II interferon (IFN-γ); ctDNA: circulating tumor DNA; dNLR: derived neutrophil-to-lymphocyte ratio; FT3: free triiodothyronine; FT4: free thyroxine; Gal-3: galectin-3; HR: hazard ratio; IL-6: interleukin-6; IL-8: interleukin-8; LDH: lactate dehydrogenase; Leu: leukocytes; LMR: lymphocyte-to-monocyte ratio; Ly: lymphocytes; MART1-mRNA: melanoma antigen recognized by T cells 1 messenger RNA; MIA: melanoma inhibitory activity protein; miR: microRNA; Mono: monocytes; Neu: neutrophils; NLR: neutrophil-to-lymphocyte ratio; p-proteasome: circulating 20S proteasome; PLR: platelet-to-lymphocyte ratio; S100: S100 calcium-binding protein; SII: systemic immune-inflammation index; TG: thyroglobulin; TK activity: thymidine kinase activity; TSH: thyroid-stimulating hormone; VEGF: vascular endothelial growth factor; WBC: white blood cell count

## **Supplementary Table S3:** Pairwise comparisons of prognostic associations between serum biomarkers across therapeutic subgroups.

| group1 | group2 | k1 | k2 | Ratio [CI_lower_; CI_upper_] | se | z | p |
| --- | --- | --- | --- | --- | --- | --- | --- |
| BRAFMEKi, ctDNA | BRAFMEKi, LDH | 7 | 6 | 0.7141 [0.4270; 1.1942] | 0.2624 | -1.2836 | 0.1993 |
| BRAFMEKi, ctDNA | ICI, CRP | 7 | 6 | 1.1666 [0.7230; 1.8825] | 0.2441 | 0.6312 | 0.5279 |
| BRAFMEKi, ctDNA | ICI, ctDNA | 7 | 5 | 0.4708 [0.2525; 0.8776] | 0.3178 | -2.3710 | 0.0177 |
| BRAFMEKi, ctDNA | ICI, IL-6 | 7 | 6 | 0.6672 [0.4088; 1.0889] | 0.2499 | -1.6191 | 0.1054 |
| BRAFMEKi, ctDNA | ICI, LDH | 7 | 23 | 0.8669 [0.5903; 1.2730] | 0.1961 | -0.7286 | 0.4662 |
| BRAFMEKi, ctDNA | ICI, Neu | 7 | 3 | 1.0883 [0.5700; 2.0779] | 0.3300 | 0.2563 | 0.7977 |
| BRAFMEKi, ctDNA | ICI, NLR | 7 | 9 | 0.8616 [0.5410; 1.3721] | 0.2374 | -0.6275 | 0.5304 |
| BRAFMEKi, ctDNA | ICI, S100 | 7 | 3 | 0.7891 [0.4002; 1.5557] | 0.3463 | -0.6840 | 0.4940 |
| BRAFMEKi, ctDNA | Other therapies, CRP | 7 | 5 | 0.9119 [0.5403; 1.5392] | 0.2671 | -0.3451 | 0.7300 |
| BRAFMEKi, ctDNA | Other therapies, LDH | 7 | 15 | 1.1373 [0.7558; 1.7114] | 0.2085 | 0.6170 | 0.5373 |
| BRAFMEKi, ctDNA | Other therapies, S100 | 7 | 5 | 0.9585 [0.5528; 1.6620] | 0.2808 | -0.1510 | 0.8799 |
| BRAFMEKi, LDH | ICI, CRP | 6 | 6 | 1.6337 [0.9721; 2.7456] | 0.2649 | 1.8532 | 0.0638 |
| BRAFMEKi, LDH | ICI, ctDNA | 6 | 5 | 0.6592 [0.3426; 1.2686] | 0.3340 | -1.2477 | 0.2122 |
| BRAFMEKi, LDH | ICI, IL-6 | 6 | 6 | 0.9344 [0.5502; 1.5868] | 0.2702 | -0.2512 | 0.8016 |
| BRAFMEKi, LDH | ICI, LDH | 6 | 23 | 1.2140 [0.7867; 1.8733] | 0.2213 | 0.8761 | 0.3810 |
| BRAFMEKi, LDH | ICI, Neu | 6 | 3 | 1.5240 [0.7741; 3.0003] | 0.3456 | 1.2192 | 0.2228 |
| BRAFMEKi, LDH | ICI, NLR | 6 | 9 | 1.2066 [0.7267; 2.0033] | 0.2587 | 0.7259 | 0.4679 |
| BRAFMEKi, LDH | ICI, S100 | 6 | 3 | 1.1050 [0.5443; 2.2431] | 0.3612 | 0.2764 | 0.7822 |
| BRAFMEKi, LDH | Other therapies, CRP | 6 | 5 | 1.2771 [0.7289; 2.2376] | 0.2861 | 0.8547 | 0.3927 |
| BRAFMEKi, LDH | Other therapies, LDH | 6 | 15 | 1.5926 [1.0099; 2.5117] | 0.2324 | 2.0022 | 0.0453 |
| BRAFMEKi, LDH | Other therapies, S100 | 6 | 5 | 1.3422 [0.7470; 2.4119] | 0.2990 | 0.9843 | 0.3250 |
| ICI, CRP | ICI, ctDNA | 6 | 5 | 0.4035 [0.2156; 0.7553] | 0.3199 | -2.8374 | 0.0045 |
| ICI, CRP | ICI, IL-6 | 6 | 6 | 0.5719 [0.3486; 0.9382] | 0.2526 | -2.2124 | 0.0269 |
| ICI, ctDNA | ICI, LDH | 5 | 23 | 1.8415 [1.0537; 3.2183] | 0.2848 | 2.1435 | 0.0321 |
| ICI, ctDNA | Other therapies, LDH | 5 | 15 | 2.4159 [1.3589; 4.2949] | 0.2936 | 3.0048 | 0.0027 |
| ICI, IL-6 | Other therapies, LDH | 6 | 15 | 1.7045 [1.1112; 2.6146] | 0.2183 | 2.4429 | 0.0146 |
| Other therapies, CRP | Other therapies, LDH | 5 | 15 | 1.2471 [0.7826; 1.9873] | 0.2377 | 0.9288 | 0.3530 |
| Other therapies, LDH | Other therapies, S100 | 15 | 5 | 0.8428 [0.5132; 1.3841] | 0.2531 | -0.6758 | 0.4992 |

Note: Pairwise comparisons were performed using meta-regression–based indirect analyses. Relative effects represent differences in the strength of prognostic associations between biomarkers.
Abbreviations: BRAFMEKi: BRAF and MEK inhibitor therapy; ICI: immune checkpoint inhibitor therapy; CRP: C-reactive protein; ctDNA: circulating tumor DNA; IL-6: interleukin-6; LDH: lactate dehydrogenase; Neu: neutrophil count; NLR: neutrophil-to-lymphocyte ratio; S100: S100B protein; CI: confidence interval; SE: standard error; k: number of studies.

## **Supplementary Figure S3:** Forest plot of univariate Hazard Ratios (HR) of overall survival (OS) and baseline lactate dehydrogenase (LDH) level associations in stage III-IV stage melanoma patients.

**Abbreviations:** CI: confidence interval; HR: hazard ratio; Log: logarithm; SE: standard error

## **Supplementary Figure S4:** Forest plot of univariate Hazard Ratios (HR) of overall survival (OS) and baseline serum lactate dehydrogenase (LDH) level associations subgrouped based on therapeutic agent and disease stage in melanoma patients.

Abbreviations: BRAF–MEK inhibitors: BRAF and MEK kinase inhibitors; CI: confidence interval; HR: hazard ratio; Log: logarithm; SE: standard error

## **Supplementary Figure S5:** Forest plot of univariate Hazard Ratios (HR) of overall survival (OS) and baseline circulating tumor DNA (ctDNA) level associations in stage III-IV stage melanoma patients.

Abbreviations: CI: confidence interval; HR: hazard ratio; Log: logarithm; SE: standard error

## **Supplementary Figure S6**: Forest plot of univariate Hazard Ratios (HR) of overall survival (OS) and baseline serum circulating tumor DNA (ctDNA) level associations subgrouped based on therapeutic agent and disease stage in melanoma patients.

Abbreviations: BRAF–MEK inhibitors: BRAF and MEK kinase inhibitors; CI: confidence interval; HR: hazard ratio; Log: logarithm; SE: standard error.

## **Supplementary Figure S7:** Forest plot of univariate Hazard Ratios (HR) of overall survival (OS) and baseline S100 level associations in stage III-IV stage melanoma patients.

Abbreviations: CI: confidence interval; HR: hazard ratio; Log: logarithm; SE: standard error

## **Supplementary Figure S8:** Forest plot of univariate Hazard Ratios (HR) of overall survival (OS) and baseline serum S100 level associations subgrouped based on therapeutic agent and disease stage in melanoma patients.

Abbreviations: BRAF–MEK inhibitors: BRAF and MEK kinase inhibitors; CI: confidence interval; HR: hazard ratio; Log: logarithm; SE: standard error.

## **Supplementary Figure S9:** Forest plot of univariate Hazard Ratios (HR) of overall survival (OS) and baseline neutrophil-to-lymphocyte ratio (NLR) associations in stage III-IV stage melanoma patients.

Abbreviations: BRAF–MEK inhibitors: BRAF and MEK kinase inhibitors; CI: confidence interval; HR: hazard ratio; Log: logarithm; SE: standard error.

## **Supplementary Figure S10:** Forest plot of univariate Hazard Ratios (HR) of overall survival (OS) and baseline neutrophil-to-lymphocyte ratio (NLR) associations subgrouped based on therapeutic agent and disease stage in melanoma patients.

Abbreviations: BRAF–MEK inhibitors: BRAF and MEK kinase inhibitors; CI: confidence interval; HR: hazard ratio; Log: logarithm; SE: standard error.

## **Supplementary Figure S11:** Forest plot of univariate Hazard Ratios (HR) of overall survival (OS) and baseline interleukin-6 (IL-6) level associations in stage III-IV stage melanoma patients.

Abbreviations: BRAF–MEK inhibitors: BRAF and MEK kinase inhibitors; CI: confidence interval; HR: hazard ratio; Log: logarithm; SE: standard error.

## **Supplementary Figure S12:** Forest plot of univariate Hazard Ratios (HR) of overall survival (OS) and baseline serum interleukin-6 (IL-6) level associations subgrouped based on therapeutic agent and disease stage in melanoma patients.

Abbreviations: BRAF–MEK inhibitors: BRAF and MEK kinase inhibitors; CI: confidence interval; HR: hazard ratio; Log: logarithm; SE: standard error.

## **Supplementary Figure S13:** Forest plot of univariate Hazard Ratios (HR) of overall survival (OS) and baseline C-reactive protein (CPR) level associations in stage III-IV stage melanoma patients.

Abbreviations: BRAF–MEK inhibitors: BRAF and MEK kinase inhibitors; CI: confidence interval; HR: hazard ratio; Log: logarithm; SE: standard error.

## **Supplementary Figure S14:** Forest plot of univariate Hazard Ratios (HR) of overall survival (OS) and baseline serum C-reactive protein (CRP) level associations subgrouped based on therapeutic agent and disease stage in melanoma patients.

Abbreviations: BRAF–MEK inhibitors: BRAF and MEK kinase inhibitors; CI: confidence interval; HR: hazard ratio; Log: logarithm; SE: standard error.

## **Supplementary Figure S15:** Forest plot of univariate Hazard Ratios (HR) of overall survival (OS) and serum biomarker levels association in melanoma patients during follow-up.

**Abbreviations:** BRAF–MEK inhibitors: BRAF and MEK kinase inhibitors; CI: confidence interval; ctDNA: circulating tumor DNA; HR: hazard ratio; LDH: lactate dehydrogenase; LMR: lymphocyte-to-monocyte ratio; Log: logarithm; NLR: neutrophil-to-lymphocyte ratio; PLR: platelet-to-lymphocyte ratio; SE: standard error

# **Overall Survival: Mutlivariable-Analyses**

## **Supplementary Figure S16:** Forest plot of multivariable-adjusted HRs for OS associated with baseline serum LDH in melanoma patients.

Abbreviations: EUR: European Region; WPR: Western Pacific Region; AMR: Region of the Americas; HR: hazard ratio; CI: confidence interval.

## **Supplementary Table S4:** Multivariable random-effects meta-regression model for the association between baseline LDH levels and OS.

| Variable | Estimate | SE | z | p | CI_lower_ | CI_upper_ |
| --- | --- | --- | --- | --- | --- | --- |
| Intercept | 0.4342 | 0.1239 | 3.5055 | 0.0005 | 0.1915 | 0.6770 |
| Cut-off value | 0.0008 | 0.0004 | 2.2699 | 0.0232 | 0.0001 | 0.0016 |

Abbreviations: SE: Standard Error; CI: Confidence Interval

## **Supplementary Figure S17:** Forest plot of multivariable-adjusted HRs for OS associated with baseline serum IL-6 in melanoma patients.

Abbreviations: EUR: European Region; AMR: Region of the Americas; HR: hazard ratio; CI: confidence interval.

## **Supplementary Table S5:** Multivariable random-effects meta-regression model for the association between baseline IL-6 levels and OS.

| Variable | Estimate | SE | z | p | CI_lower_ | CI_upper_ |
| --- | --- | --- | --- | --- | --- | --- |
| Intercept | 0.3765 | 0.1582 | 2.3800 | 0.0173 | 0.0665 | 0.6866 |
| Cut-off value | 0.0591 | 0.0205 | 2.8818 | 0.0040 | 0.0189 | 0.0994 |

Abbreviations: SE: Standard Error; CI: Confidence Interval

## **Supplementary Figure S18:** Forest plot of multivariable-adjusted HRs for OS associated with baseline serum NLR in melanoma patients.

Abbreviations: AMR: Region of the Americas; EUR: European Region; WPR: Western Pacific Region; HR: hazard ratio; CI: confidence interval.

## **Supplementary Table S6:** Multivariable random-effects meta-regression model for the association between baseline NLR levels and OS.

| Variable | Estimate | SE | z | p | CI_lower_ | CI_upper_ |
| --- | --- | --- | --- | --- | --- | --- |
| NLR | 0.6215 | 0.1499 | 4.1458 | <0.0001 | 0.3277 | 0.9153 |

Abbreviations: SE: Standard Error; CI: Confidence Interval

## **Supplementary Figure S19:** Forest plot of multivariable-adjusted HRs for OS associated with baseline ctDNA levels in melanoma patients.

Abbreviations: AMR: Region of the Americas; EUR: European Region; WPR: Western Pacific Region; HR: hazard ratio; CI: confidence interval.

## **Supplementary Figure S20:** Forest plot of multivariable-adjusted HRs for OS associated with baseline S100B levels in melanoma patients.

Abbreviations: EUR: European Region; HR: hazard ratio; CI: confidence interval.

## **Supplementary Figure S21:** Forest plot of multivariable-adjusted HRs for OS associated with baseline CRP levels in melanoma patients.

Abbreviations: AMR: Region of the Americas; EUR: European Region; HR: hazard ratio; CI: confidence interval.

## **Supplementary Table S7:** Multivariable random-effects meta-regression model for the association between baseline CRP levels and OS.

| Variable | Estimate | SE | z | p | CI_lower_ | CI_upper_ |
| --- | --- | --- | --- | --- | --- | --- |
| Intercept | 0.3317 | 0.0777 | 4.2671 | <0.0001 | 0.1793 | 0.4841 |
| Other therapies | 0.5241 | 0.1268 | 4.1320 | <0.0001 | 0.2755 | 0.7727 |

Abbreviations: SE: Standard Error; CI: Confidence Interval

# **Progression-Free Survival: Univariate analyses**

## **Supplementary Figure S22:** Forest plot of univariate Hazard Ratios (HR) of progression-free survival (PFS) and baseline serum biomarker level associations in melanoma patients, regardless of therapeutic agent.

**Abbreviations:** ALC: absolute lymphocyte count; ANC: absolute neutrophil count; anti-TPO: anti-thyroid peroxidase antibody; CD37 activity: cluster of differentiation 37 activity; cfDNA: cell-free DNA; CI: confidence interval; CRP: C-reactive protein; CTC: circulating tumor cell; ctDNA: circulating tumor DNA; dNLR: derived neutrophil-to-lymphocyte ratio; HGF: hepatocyte growth factor; HR: hazard ratio; HSP90α: heat shock protein 90 alpha; IL-6: interleukin-6; IL-18: interleukin-18; log: logarithm; LMR: lymphocyte-to-monocyte ratio; Ly: lymphocytes; MIA: melanoma inhibitory activity; Neu: neutrophils; p-proteasome: circulating 20S proteasome; PLR: platelet-to-lymphocyte ratio; S100: S100 calcium-binding protein; SE: standard error; SII index: systemic immune-inflammation index; TK activity: thymidine kinase activity; WBC: white blood cell count

## **Supplementary Table S8:** Pairwise comparisons of prognostic associations between serum biomarkers and PFS regardless of therapeutic agent.

| group1 | group2 | k1 | k2 | Ratio [CI_lower; CI_upper] | SE | z | p |
| --- | --- | --- | --- | --- | --- | --- | --- |
| CRP | ctDNA | 3 | 15 | 0.5874 [0.3198; 1.0787] | 0.3102 | -1.7156 | 0.0862 |
| CRP | LDH | 3 | 27 | 0.7495 [0.4236; 1.3262] | 0.2911 | -0.9903 | 0.3220 |
| CRP | NLR | 3 | 11 | 0.7122 [0.3860; 1.3141] | 0.3125 | -1.0859 | 0.2775 |
| CRP | S100 | 3 | 5 | 0.8226 [0.4030; 1.6792] | 0.3641 | -0.5362 | 0.5918 |
| ctDNA | LDH | 15 | 27 | 1.2761 [0.9018; 1.8059] | 0.1772 | 1.3764 | 0.1687 |
| ctDNA | NLR | 15 | 11 | 1.2126 [0.8026; 1.8318] | 0.2105 | 0.9156 | 0.3599 |
| ctDNA | S100 | 15 | 5 | 1.4006 [0.8068; 2.4313] | 0.2814 | 1.1972 | 0.2312 |
| LDH | NLR | 27 | 11 | 0.9502 [0.6661; 1.3555] | 0.1813 | -0.2819 | 0.7780 |
| LDH | S100 | 27 | 5 | 1.0975 [0.6590; 1.8279] | 0.2603 | 0.3575 | 0.7207 |
| NLR | S100 | 11 | 5 | 1.1551 [0.6620; 2.0154] | 0.2840 | 0.5076 | 0.6117 |

Note: Pairwise comparisons were performed using meta-regression–based indirect analyses. Relative effects represent differences in the strength of prognostic associations between biomarkers.
Abbreviations: CRP: C-reactive protein; ctDNA: circulating tumor DNA; LDH: lactate dehydrogenase; Neu: neutrophil count; confidence interval; SE: standard error; k: number of studies

## **Supplementary Figure S23:** Forest plot of univariate Hazard Ratios (HR) of progression free survival (PFS) and baseline serum biomarker level associations in melanoma patients subgrouped based on therapeutic agent.

**Abbreviations:** ALC: absolute lymphocyte count; ANC: absolute neutrophil count; anti-TPO: anti-thyroid peroxidase antibody; CD37 activity: cluster of differentiation 37 activity; CI: confidence interval; CRP: C-reactive protein; CTC: circulating tumor cell; ctDNA: circulating tumor DNA; dNLR: derived neutrophil-to-lymphocyte ratio; HR: hazard ratio; IL-6: interleukin-6; IL-18: interleukin-18; LDH: lactate dehydrogenase; log: logarithm; Ly: lymphocytes; Neu: neutrophils; NLR: neutrophil-to-lymphocyte ratio; S100: S100 calcium-binding protein; SE: standard error; TK activity: thymidine kinase activity; WBC: white blood cell count.

## **Supplementary Table S9:** Pairwise comparisons of prognostic associations between serum biomarkers and PFS across therapeutic subgroups.

| group1 | group2 | k1 | k2 | Ratio [CI_lower; CI_upper] | se | z | p |
| --- | --- | --- | --- | --- | --- | --- | --- |
| BRAFMEKi, ctDNA | BRAFMEKi, LDH | 7 | 8 | 1.1323 [0.6448; 1.9882] | 0.2873 | 0.4324 | 0.6654 |
| BRAFMEKi, ctDNA | ICI, ctDNA | 7 | 4 | 0.6081 [0.2750; 1.3445] | 0.4048 | -1.2288 | 0.2191 |
| BRAFMEKi, ctDNA | ICI, LDH | 7 | 11 | 1.2911 [0.7680; 2.1705] | 0.2650 | 0.9641 | 0.3350 |
| BRAFMEKi, ctDNA | ICI, NLR | 7 | 8 | 1.1650 [0.6654; 2.0397] | 0.2857 | 0.5346 | 0.5929 |
| BRAFMEKi, ctDNA | Other therapies, ctDNA | 7 | 4 | 0.9895 [0.4920; 1.9900] | 0.3565 | -0.0297 | 0.9763 |
| BRAFMEKi, ctDNA | Other therapies, LDH | 7 | 8 | 0.9730 [0.5342; 1.7725] | 0.3060 | -0.0894 | 0.9288 |
| BRAFMEKi, ctDNA | Other therapies, S100 | 7 | 4 | 1.3051 [0.6519; 2.6128] | 0.3542 | 0.7518 | 0.4522 |
| BRAFMEKi, LDH | ICI, ctDNA | 8 | 4 | 0.5370 [0.2489; 1.1588] | 0.3924 | -1.5843 | 0.1131 |
| BRAFMEKi, LDH | ICI, LDH | 8 | 11 | 1.1403 [0.7046; 1.8454] | 0.2456 | 0.5345 | 0.5930 |
| BRAFMEKi, LDH | ICI, NLR | 8 | 8 | 1.0290 [0.6087; 1.7394] | 0.2679 | 0.1066 | 0.9151 |
| BRAFMEKi, LDH | Other therapies, ctDNA | 8 | 4 | 0.8739 [0.4467; 1.7094] | 0.3423 | -0.3938 | 0.6937 |
| BRAFMEKi, LDH | Other therapies, LDH | 8 | 8 | 0.8594 [0.4874; 1.5152] | 0.2893 | -0.5238 | 0.6004 |
| BRAFMEKi, LDH | Other therapies, S100 | 8 | 4 | 1.1526 [0.5921; 2.2439] | 0.3399 | 0.4179 | 0.6760 |
| ICI, ctDNA | ICI, LDH | 4 | 11 | 2.1234 [1.0153; 4.4407] | 0.3764 | 2.0003 | 0.0455 |
| ICI, ctDNA | ICI, NLR | 4 | 8 | 1.9160 [0.8899; 4.1255] | 0.3913 | 1.6617 | 0.0966 |
| ICI, ctDNA | Other therapies, ctDNA | 4 | 4 | 1.6272 [0.6794; 3.8972] | 0.4456 | 1.0926 | 0.2746 |
| ICI, ctDNA | Other therapies, LDH | 4 | 8 | 1.6002 [0.7216; 3.5484] | 0.4063 | 1.1571 | 0.2472 |
| ICI, ctDNA | Other therapies, S100 | 4 | 4 | 2.1463 [0.8994; 5.1216] | 0.4437 | 1.7211 | 0.0852 |
| ICI, LDH | ICI, NLR | 11 | 8 | 0.9024 [0.5595; 1.4553] | 0.2439 | -0.4214 | 0.6735 |
| ICI, LDH | Other therapies, ctDNA | 11 | 4 | 0.7664 [0.4062; 1.4459] | 0.3239 | -0.8216 | 0.4113 |
| ICI, LDH | Other therapies, LDH | 11 | 8 | 0.7536 [0.4463; 1.2725] | 0.2673 | -1.0583 | 0.2899 |
| ICI, LDH | Other therapies, S100 | 11 | 4 | 1.0108 [0.5385; 1.8975] | 0.3213 | 0.0334 | 0.9733 |
| ICI, NLR | Other therapies, ctDNA | 8 | 4 | 0.8493 [0.4352; 1.6572] | 0.3411 | -0.4790 | 0.6319 |
| ICI, NLR | Other therapies, LDH | 8 | 8 | 0.8352 [0.4751; 1.4682] | 0.2878 | -0.6257 | 0.5315 |
| ICI, NLR | Other therapies, S100 | 8 | 4 | 1.1202 [0.5768; 2.1753] | 0.3386 | 0.3352 | 0.7375 |
| Other therapies, ctDNA | Other therapies, LDH | 4 | 8 | 0.9834 [0.4873; 1.9843] | 0.3582 | -0.0467 | 0.9627 |
| Other therapies, ctDNA | Other therapies, S100 | 4 | 4 | 1.3190 [0.6021; 2.8896] | 0.4001 | 0.6919 | 0.4890 |
| Other therapies, LDH | Other therapies, S100 | 8 | 4 | 1.3413 [0.6677; 2.6942] | 0.3559 | 0.8250 | 0.4093 |

Note: Pairwise comparisons were performed using meta-regression–based indirect analyses. Relative effects represent differences in the strength of prognostic associations between biomarkers.
Abbreviations: BRAFMEKi: BRAF and MEK inhibitor therapy; ICI: immune checkpoint inhibitor therapy; CRP: C-reactive protein; ctDNA: circulating tumor DNA; IL-6: interleukin-6; LDH: lactate dehydrogenase; NLR: neutrophil-to-lymphocyte ratio; S100: S100B protein; CI: confidence interval; SE: standard error; k: number of studies

## **Supplementary Figure S24:** Forest plot of univariate Hazard Ratios (HR) of progression-free survival (PFS) and baseline lactate dehydrogenase (LDH) level associations in stage III-IV stage melanoma patients.

Abbreviations: CI: confidence interval; HR: hazard ratio; Log: logarithm; SE: standard error.

## **Supplementary Figure S25:** Forest plot of univariate Hazard Ratios (HR) of progression-free survival (PFS) and baseline serum lactate dehydrogenase (LDH) level associations subgrouped based on therapeutic agent and disease stage in melanoma patients.

Abbreviations: BRAF–MEK inhibitors: BRAF and MEK kinase inhibitors; CI: confidence interval; HR: hazard ratio; Log: logarithm; SE: standard error

## **Supplementary Figure S26:** Forest plot of univariate Hazard Ratios (HR) of progression-free survival (PFS) and baseline circulating tumor DNA (ctDNA) level associations in stage III-IV stage melanoma patients.

Abbreviations: confidence interval; HR: hazard ratio; Log: logarithm; SE: standard error

## **Supplementary Figure S27:** Forest plot of univariate Hazard Ratios (HR) of progression-free survival (PFS) and baseline serum circulating tumor DNA (ctDNA) level associations subgrouped based on therapeutic agent and disease stage in melanoma patients.

Abbreviations: BRAF–MEK inhibitors: BRAF and MEK kinase inhibitors; CI: confidence interval; HR: hazard ratio; Log: logarithm; SE: standard error

## **Supplementary Figure S28:** Forest plot of univariate Hazard Ratios (HR) of progression-free survival (PFS) and baseline serum circulating tumor DNA (ctDNA) level associations in different therapeutic subgroups in melanoma patients.

Abbreviations: BRAF–MEK inhibitors: BRAF and MEK kinase inhibitors; CI: confidence interval; HR: hazard ratio; Log: logarithm; SE: standard error

## **Supplementary Figure S29:** Forest plot of univariate Hazard Ratios (HR) of progression-free survival (PFS) and baseline neutrophil-to-lymphocyte ratio (NLR) associations in stage III-IV stage melanoma patients.

Abbreviations: CI: confidence interval; HR: hazard ratio; Log: logarithm; SE: standard error

## **Supplementary Figure S30:** Forest plot of univariate Hazard Ratios (HR) of progression-free survival (PFS) and baseline neutrophil-to-lymphocyte ratio (NLR) associations subgrouped based on therapeutic agent and disease stage in melanoma patients.

Abbreviations: BRAF–MEK inhibitors: BRAF and MEK kinase inhibitors; CI: confidence interval; HR: hazard ratio; Log: logarithm; SE: standard error

## **Supplementary Figure S31:** Forest plot of univariate Hazard Ratios (HR) of progression-free survival (PFS) and serum biomarker levels association in melanoma patients during follow-up.

**Abbreviations:** CI: confidence interval; ctDNA: circulating tumor DNA; HR: hazard ratio; LDH: lactate dehydrogenase; LMR: lymphocyte-to-monocyte ratio; log: logarithm; NLR: neutrophil-to-lymphocyte ratio; PLR: platelet-to-lymphocyte ratio; S100: S100 calcium-binding protein; SE: standard error

# **Progression-Free-Survival: Multivariable analyses**

## **Supplementary Figure S32:** Forest plot of multivariable-adjusted HRs for PFS associated with baseline LDH levels in melanoma.

Abbreviations: AMR: Region of the Americas; EUR: European Region; HR: hazard ratio; CI: confidence interval.

## **Supplementary Table S10:** Multivariable random-effects meta-regression model for the association between baseline LDH levels and PFS.

| Estimate | SE | z | p | CI_lower_ | CI_upper_ |
| --- | --- | --- | --- | --- | --- |
| 0.5354 | 0.0920 | 5.8171 | <.0001 | 0.3550 | 0.7157 |

Abbreviations: SE: Standard Error; CI: Confidence Interval

## **Supplementary Figure S33:** Forest plot of multivariable-adjusted HRs for PFS associated with baseline ctDNA levels in melanoma.

Abbreviations: EUR: European Region; WPR: Western Pacific Region; HR: hazard ratio; CI: confidence interval.

## **Supplementary Figure S34:** Forest plot of multivariable-adjusted HRs for PFS associated with baseline NLR levels in melanoma.

Abbreviations: EUR: European Region; WPR: Western Pacific Region; HR: hazard ratio; CI: confidence interval.

## **Supplementary Figure S35:** Forest plot of multivariable-adjusted HRs for PFS associated with baseline CRP levels in melanoma.

Abbreviations: EUR: European Region; WPR: Western Pacific Region; HR: hazard ratio; CI: confidence interval.

# **Distant metastasis disease-free- (DM-DFS), disease-free-(DSS) and recurrance-free (RFS) survival: univariate analyses**

## **Supplementary Figure S36:** Forest plot of univariate Hazard Ratios (HR) of distant metastasis disease-free survival (DM-DFS) and serum biomarker levels association in melanoma patients during follow-up.

**Abbreviations:** CI: confidence interval; ctDNA: circulating tumor DNA; HR: hazard ratio; log: logarithm; S100: S100 calcium-binding protein; SE: standard error

## **Supplementary Figure S37:** Forest plot of univariate Hazard Ratios (HR) of disease-specific survival (DSS) and baseline serum biomarker level associations in melanoma patients, regardless of therapeutic agent.

**Abbreviations:** CI: confidence interval; CRP: C-reactive protein; ctDNA: circulating tumor DNA; HR: hazard ratio; LDH: lactate dehydrogenase; log: logarithm; S100: S100 calcium-binding protein; SE: standard error

## **Supplementary Table S11:**Pairwise comparisons of prognostic associations between serum biomarkers for DSS.

| group1 | group2 | k1 | k2 | Ratio [CI_lower_; CI_upper_] | se | z | p |
| --- | --- | --- | --- | --- | --- | --- | --- |
| ctDNA | LDH | 4 | 5 | 0.736 [0.4930;1.0998]3 | 0.2047 | -1.4952 | 0.1349 |
| ctDNA | S100 | 4 | 3 | 1.0120 [0.6574;1.5577] | 0.2201 | 0.0541 | 0.9568 |
| LDH | S100 | 5 | 3 | 1.3743 [0.8880;2.1272] | 0.2229 | 1.4267 | 0.1537 |

Note: Pairwise comparisons were performed using meta-regression–based indirect analyses. Relative effects represent differences in the strength of prognostic associations between biomarkers.
Abbreviations: ctDNA: circulating tumor DNA; LDH: lactate dehydrogenase; CI: confidence interval; SE: standard error.

## **Supplementary Figure S38:** Forest plot of univariate Hazard Ratios (HR) of recurrence-free survival (RFS) and baseline serum biomarker level associations in melanoma patients, regardless of therapeutic agent.

**Abbreviations:** CI: confidence interval; CRP: C-reactive protein; CTC: circulating tumor cell; ctDNA: circulating tumor DNA; HR: hazard ratio; LDH: lactate dehydrogenase; LMR: lymphocyte-to-monocyte ratio; log: logarithm; NLR: neutrophil-to-lymphocyte ratio; PLR: platelet-to-lymphocyte ratio; SE: standard error.

# **Distant metastasis disease-free survival and disease-free survival: multivariable analyses**

## **Supplementary Figure S39:** Forest plot of multivariable-adjusted HRs for DM-DFS associated with baseline S100B levels in melanoma patients.

## **Supplementary Figure S40:** Forest plot of multivariable-adjusted HRs for DSS associated with baseline LDH levels in melanoma patients.

## **Supplementary Figure S41:** Forest plot of multivariable-adjusted HRs for DSS associated with baseline S100B levels in melanoma.

# **Risk of Bias assessment**

## **Supplementary Table S12:** Risk of bias and concerns regarding applicability assessment using the QUIPS (Quality In Prognosis Studies) tool.

| **Author,Year** | **Study participation** | **Study attrition** | **Prognostic Factor measurement** | **Outcome Measures** | | | | | | **Study Confounding** | **Statistical Analysis Reporting** | **Overall Risk of Bias** |
| --- | --- | --- | --- | --- | --- | --- | --- | --- | --- | --- | --- | --- |
|  |  |  |  | **OS** | **PFS** | **DSS** | **DFS** | **DMFS** | **RFS** |  |  |  |
| Aamadal_2021 [1] | moderate | low | low | low | low | NA | NA | NA | NA | low | low | moderate |
| Abu-Sbeih_2019 [2] | moderate | low | low | low | low | NA | NA | NA | NA | unclear | low | moderate |
| Amaral_2020 [3] | moderate | low | low | low | NA | NA | NA | NA | NA | low | low | modetare |
| Andressa_2020 [4] | low | low | low | low | NA | NA | NA | NA | NA | low | low | low |
| Aoude_2023 [55] | moderate | moderate | low | NA | NA | low | NA | NA | NA | unclear | low | high |
| Arheden_2020 [5] | moderate | low | low | low | NA | NA | NA | NA | NA | low | low | moderate |
| Awada_2021 [56] | low | low | low | low | low | NA | NA | NA | NA | unclear | low | moderate |
| Bhatia_2019 [6] | low | low | low | low | NA | NA | NA | NA | NA | low | low | low |
| Boerlin_2022 [57] | moderate | low | low | low | low | NA | NA | NA | NA | low | low | moderate |
| Braune_2020 [58] | high | low | low | NA | low | NA | NA | NA | NA | high | moderate | high |
| Capone_2018 [7] | moderate | moderate | low | low | low | NA | NA | NA | NA | moderate | low | high |
| Chiarion.Sileni_2003 [62] | low | unclear | low | low | NA | NA | NA | NA | NA | low | low | moderate |
| Cocorocchio_2020 [40] | moderate | low | low | NA | low | NA | NA | NA | NA | low | low | moderate |
| Czirbesz_2017 [41] | moderate | moderate | moderate | low | low | NA | NA | NA | NA | moderate | moderate | high |
| Damuzzo_2016 [8] | low | low | low | low | NA | NA | NA | NA | NA | unclear | low | low |
| Desch_2016 [64] | moderate | low | low | low | low | NA | NA | NA | NA | unclear | low | moderate |
| Eroglu_2023 [9] | moderate | low | low | NA | low | NA | low | NA | NA | high | low | high |
| Fang_2015 [66] | moderate | unclear | low | low | NA | low | low | NA | NA | unclear | low | moderate |
| Filia_2013 [111] | low | low | moderate | low | NA | low | NA | NA | NA | moderate | low | moderate |
| Finon_2018 [44] | moderate | low | low | NA | low | NA | NA | NA | NA | low | low | moderate |
| Forthun_2019 [67] | high | low | moderate | low | low | NA | NA | NA | NA | moderate | low | high |
| Garlan_2017 [45] | high | moderate | low | low | low | NA | NA | NA | NA | moderate | moderate | high |
| Gassenmeier_2021 [46] | low | moderate | low | NA | low | low | NA | NA | NA | moderate | low | moderate |
| Gonzalet-Cao_2018 [68] | low | low | moderate | low | low | NA | NA | NA | NA | moderate | low | moderate |
| Gray_2014[69] | moderate | low | low | low | low | NA | NA | NA | NA | low | low | moderate |
| Gray_2015[10] | moderate | low | low | NA | low | NA | NA | NA | NA | low | low | moderate |
| Heidelberger_2017 [11] | moderate | low | low | NA | low | NA | NA | NA | NA | unclear | low | moderate |
| Herbertreau_2018 [12] | moderate | low | low | low | low | NA | NA | NA | NA | moderate | low | moderate |
| Hoejberg_2012 [13] | moderate | low | low | low | NA | NA | NA | NA | NA | low | low | moderate |
| Hügel_2016 [71] | moderate | moderate | low | low | NA | NA | NA | NA | NA | low | low | moderate |
| Interno_2023 [73] | moderate | low | low | low | NA | NA | NA | NA | NA | low | low | moderate |
| K.P.Wevers_2013 [75] | low | Moderate | moderate | NA | NA | NA | low | NA | NA | low | moderate | high |
| Koguchi_2015 [16] | low | low | low | low | NA | NA | NA | NA | NA | low | low | low |
| Kopecky_2020 [74] | moderate | low | low | low | low | NA | NA | NA | NA | low | low | moderate |
| Kozak, 2019 [47] | low | moderate | low | low | low | NA | NA | NA | NA | low | low | moderate |
| Krkowski, 2021 [17] | moderate | moderate | low | low | low | NA | NA | NA | NA | low | low | moderate |
| Lee, 2017 [18] | low | moderate | low | low | low | NA | NA | NA | NA | low | low | moderate |
| Lee, 2018 [76] | low | low | low | low | NA | NA | NA | NA | NA | low | low | low |
| Lee, 2019 [127] | low | low | moderate | low | NA | NA | NA | NA | NA | unclear | low | moderate |
| Lee, 2020 [19] | low | low | low | low | NA | NA | NA | NA | NA | moderate | low | moderate |
| Long, 2016b [48] | low | low | low | low | low | NA | NA | NA | NA | moderate | low | moderate |
| Lu, 2018 [49] | low | low | low | low | NA | NA | NA | NA | NA | low | low | low |
| Mallardo, 2023 [21] | moderate | moderate | low | NA | low | NA | NA | NA | NA | moderate | low | moderate |
| Marcus, 2014 [77] | moderate | low | low | low | NA | NA | NA | NA | NA | moderate | low | moderate |
| Musaelyan, 2022 [22] | low | moderate | low | NA | moderate | NA | NA | NA | NA | unclear | low | high |
| Neuman, 2007 [80] | moderate | low | low | low | NA | NA | NA | NA | NA | low | low | moderate |
| Pedersen, 2020 [23] | moderate | low | moderate | NA | low | NA | NA | NA | NA | moderate | low | moderate |
| Reinert, 2020 [83] | moderate | moderate | low | low | NA | NA | NA | NA | NA | low | low | moderate |
| Rogala, 2022 [24] | moderate | low | low | low | low | NA | NA | NA | NA | unclear | low | moderate |
| Rose, 2021 [25] | moderate | low | low | low | low | NA | NA | NA | NA | low | low | moderate |
| Rossi, 2022 [26] | low | moderate | low | low | NA | NA | NA | NA | NA | low | low | moderate |
| Ruggiero, 2022 [50] | moderate | low | moderate | low | low | NA | NA | NA | NA | unclear | moderate | high |
| S. Ugurel, 2014 [51] | moderate | low | low | low | low | NA | NA | NA | NA | low | low | moderate |
| S.Rosner, 2018 [27] | moderate | low | moderate | moderate | NA | NA | NA | NA | NA | low | moderate | high |
| Schildbach, 2023 [147] | moderate | low | low | low | NA | NA | NA | NA | low | unclear | low | moderate |
| Schmidt, 2005 [87] | low | low | low | low | NA | NA | NA | NA | NA | low | low | low |
| Schmidt, 2005a [28] | low | low | low | low | NA | NA | NA | NA | NA | low | low | low |
| Schouwenburg, 2018 [150] | low | low | low | low | NA | NA | NA | NA | NA | low | low | low |
| Schraag, 2019 [29] | moderate | low | moderate | low | NA | NA | NA | NA | NA | low | low | moderate |
| Seremet, 2019 [30] | low | moderate | low | low | low | NA | NA | NA | NA | unclear | low | moderate |
| Simentic, 2020 [31] | moderate | low | low | low | low | NA | NA | NA | NA | moderate | low | moderate |
| Svedman, 2022 [32] | low | moderate | low | low | low | NA | NA | NA | NA | unclear | low | moderate |
| Syeda, 2022 [53] | low | low | low | low | low | NA | NA | NA | NA | moderate | low | moderate |
| Tan, 2019 [156] | low | low | low | NA | NA | NA | low | NA | low | low | low | low |
| Taques, 2020 [89] | moderate | low | low | NA | low | NA | NA | NA | NA | high | low | high |
| Tolmeijer, 2021 [90] | low | moderate | low | NA | low | low | NA | NA | NA | unclear | low | moderate |
| Trommer, 2022 [33] | moderate | low | moderate | low | NA | NA | NA | NA | NA | low | low | moderate |
| Turiello, 2020 [35] | moderate | low | low | low | low | NA | NA | NA | NA | unclear | low | moderate |
| Váraljai, 2020 [36] | low | moderate | low | moderate | moderate | NA | NA | NA | NA | unclear | low | high |
| Váraljai, 2021 [91] | low | moderate | low | low | low | NA | NA | NA | NA | low | low | moderate |
| Vereecken, 2009 [92] | high | low | low | low | NA | NA | NA | NA | NA | moderate | low | high |
| Vila, 2021 [37] | high | moderate | low | moderate | NA | NA | NA | NA | NA | low | low | high |
| Yu, 2017 [93] | moderate | low | low | low | NA | NA | NA | NA | low | low | low | moderate |
| Zaragoza, 2016 [38] | moderate | low | moderate | moderate | NA | NA | NA | NA | NA | low | low | high |
| Zeijl, 2020 [94] | low | moderate | low | low | NA | NA | NA | NA | NA | low | moderate | moderate |
| Zhang, 2021 [95] | low | moderate | moderate | NA | low | NA | NA | NA | NA | unclear | low | moderate |
| Zhao, 2022 [39] | high | low | low | low | low | NA | NA | NA | NA | low | low | high |
| A. Porter, 2021[96] | low | moderate | low | low | NA | NA | NA | NA | NA | low | low | moderate |
| Wilson, 2019 [164] | low | moderate | moderate | low | NA | NA | NA | NA | NA | low | low | moderate |
| Aglietta, 2023[97] | moderate | moderate | moderate | low | NA | NA | NA | NA | NA | moderate | low | high |
| Allen, 2011[54] | moderate | high | low | low | NA | NA | NA | NA | NA | moderate | low | high |
| B.Weide, 2012[98] | moderate | low | moderate | low | NA | NA | NA | NA | NA | moderate | moderate | high |
| Weide B, 2013[59] | moderate | low | moderate | low | NA | NA | NA | NA | NA | low | low | moderate |
| Bolander, 2008[99] | high | moderate | low | low | NA | low | NA | NA | NA | low | low | high |
| Cananzi, 2014[61] | moderate | low | low | moderate | moderate | NA | NA | NA | NA | low | low | moderate |
| Cao, 2007[100] | low | low | low | low | NA | NA | low | NA | NA | moderate | low | moderate |
| Chung, 2002[101] | low | low | low | low | NA | NA | low | NA | NA | low | moderate | moderate |
| Corrie, 2018[102] | low | low | low | low | NA | NA | NA | NA | NA | low | low | low |
| Cybulska-Stopa, 2012 [103] | low | low | moderate | low | NA | NA | NA | NA | NA | moderate | low | moderate |
| Cybluska-Stopa, 2020 [60] | moderate | low | low | low | NA | NA | NA | NA | NA | low | low | moderate |
| D.Desai, 2023[63] | low | low | low | low | NA | NA | NA | NA | NA | unclear | low | low |
| Deckers, 2020[104] | moderate | moderate | moderate | low | NA | NA | NA | NA | NA | moderate | moderate | high |
| Decoster, 2015[105] | moderate | moderate | low | moderate | moderate | NA | NA | NA | NA | high | moderate | high |
| Depenni, 2018[42] | low | low | moderate | NA | low | NA | NA | NA | NA | low | moderate | moderate |
| Domingo Doménech, 2007[106] | low | moderate | low | low | NA | NA | low | NA | NA | low | low | moderate |
| Dutriaux, 2022[43] | moderate | moderate | low | NA | low | NA | NA | NA | NA | unclear | low | moderate |
| Eton, 2002 [65] | moderate | low | low | NA | low | NA | NA | NA | NA | low | moderate | moderate |
| Fang, 2016[108] | high | unclear | moderate | low | NA | low | low | NA | NA | low | low | high |
| Faries, 2004[109] | moderate | low | low | moderate | NA | moderate | NA | NA | NA | low | low | high |
| Felix, 2016[110] | low | moderate | low | low | NA | NA | NA | NA | NA | low | low | moderate |
| Foppen, 2016 [112] | moderate | low | moderate | low | NA | NA | NA | NA | NA | low | low | moderate |
| Forschner, 2020[113] | moderate | low | moderate | low | NA | NA | NA | NA | NA | moderate | low | moderate |
| Egberts, 2008[107] | low | moderate | low | low | NA | NA | NA | NA | NA | low | low | moderate |
| Fusi, 2009 [114] | high | low | low |  | NA | NA | NA | low | NA | moderate | moderate | high |
| Guida, 2003 [115] | low | low | moderate | low | NA | NA | NA | NA | NA | moderate | low | moderate |
| Guilda, 2006[116] | high | low | low | low | low | NA | NA | NA | NA | moderate | moderate | high |
| Henry, 2010[117] | moderate | moderate | moderate | low | low | NA | NA | NA | NA | low | low | high |
| Henry, 2013[70] | moderate | moderate | low | low | low | NA | NA | NA | NA | low | low | moderate |
| Hoffmann, 2011[118] | low | low | low | NA | NA | low | low | NA | NA | low | low | low |
| Hoshimoto, 2012[119] | moderate | moderate | low | low | NA | NA | NA | NA | NA | low | low | moderate |
| Hu, 2022[120] | moderate | low | low | low | low | NA | NA | NA | NA | moderate | low | moderate |
| Ibrahim, 2020[72] | moderate | low | low | low | NA | NA | NA | NA | NA | low | low | moderate |
| Iivanainen, 2019[121] | moderate | low | low | low | low | NA | NA | NA | NA | low | low | moderate |
| J.van Not, 2023[122] | moderate | moderate | moderate | low | NA | NA | NA | NA | NA | low | low | high |
| Keilholz, 1996[85] | moderate | unclear | low | low | NA | NA | NA | NA | NA | moderate | low | moderate |
| Kelderman, 2014[14] | low | low | low | low | low | NA | NA | NA | NA | moderate | low | moderate |
| Keller, 2019[123] | moderate | low | low | NA | low | NA | NA | NA | NA | moderate | low | moderate |
| Khoja, 2013[124] | low | low | low | low | low | NA | NA | NA | NA | low | low | Low |
| Khoja, 2016[15] | moderate | low | low | low | NA | NA | NA | NA | NA | low | low | moderate |
| Koyanagi, 2010[125] | low | moderate | low | low | low | NA | NA | NA | NA | moderate | low | moderate |
| Kruijff, 2009[126] | low | moderate | low | NA | NA | NA | low | NA | NA | unclear | low | moderate |
| Levati, 2022[128] | high | moderate | low | low | low | NA | NA | NA | NA | moderate | low | high |
| Li, 2014[129] | high | low | low | low | NA | NA | low | NA | NA | unclear | low | high |
| Li, 2018[130] | low | moderate | low | NA | NA | low | NA | NA | NA | low | low | moderate |
| Li, 2021[131] | moderate | low | moderate | low | NA | NA | NA | NA | NA | moderate | low | high |
| Lin, 2018[132] | low | moderate | low | low | NA | NA | NA | NA | NA | moderate | low | moderate |
| Lu, 2023[133] | moderate | moderate | low | low | NA | NA | NA | NA | NA | moderate | low | high |
| Lucci, 2020[134] | low | low | low | NA | NA | NA | NA | NA | low | unclear | low | low |
| Lucci, 2023[135] | low | low | low | NA | NA | NA | NA | NA | low | low | low | low |
| M Staudt, 2010[136] | moderate | low | low | low | NA | low | NA | NA | NA | moderate | moderate | high |
| M.F.Sanmamed, 2017[137] | low | low | low | low | NA | NA | NA | NA | NA | moderate | low | moderate |
| Mahalingam, 2014[138] | moderate | moderate | low | NA | low | NA | NA | NA | NA | moderate | low | high |
| Meckbach, 2014[78] | moderate | low | low | low | NA | NA | NA | NA | NA | moderate | low | moderate |
| Meral, 2001[139] | moderate | moderate | moderate | low | NA | NA | NA | NA | NA | moderate | moderate | high |
| Moser, 2019[79] | moderate | low | low | low | NA | NA | NA | NA | NA | low | low | moderate |
| Partl, 2013[81] | moderate | low | low | low | NA | NA | NA | NA | NA | low | low | moderate |
| Partl, 2015[82] | moderate | low | low | low | NA | NA | NA | NA | NA | low | low | moderate |
| Pelizzari, 2023[140] | moderate | moderate | low | low | low | NA | NA | NA | NA | low | low | moderate |
| Prasmickatie, 2015[141] | low | low | low | low | NA | NA | NA | NA | low | moderate | low | moderate |
| RAO, 2011[142] | low | moderate | low | low | NA | NA | NA | NA | NA | high | low | high |
| S Hall, 2018[144] | Low | low | low | NA | NA | NA | NA | NA | low | low | low | low |
| S Diem, 2016[143] | low | Moderate | low | low | NA | NA | NA | NA | NA | low | low | moderate |
| S Kruijff, 2011[84] | low | low | low | NA | NA | low | low | NA | NA | low | low | low |
| S Valpione, 2018[86] | low | low | moderate | low | NA | NA | NA | NA | NA | moderate | low | moderate |
| Saiag, 2021[52] | low | moderate | low | NA | low | NA | NA | NA | NA | low | low | moderate |
| Sanmamed, 2014[145] | moderate | low | low | low | low | NA | NA | NA | NA | moderate | low | moderate |
| Sanmamed, 2015[146] | moderate | low | low | low | low | NA | NA | NA | NA | moderate | low | moderate |
| Schmidt, 2006[148] | moderate | low | moderate | low | NA | NA | NA | NA | low | low | low | moderate |
| Schneider, 2021[149] | moderate | low | low | low | NA | NA | NA | NA | NA | moderate | moderate | high |
| Seetharamu, 2012[151] | low | moderate | low | low | NA | NA | NA | NA | NA | moderate | low | moderate |
| Shah, 2021[152] | moderate | low | moderate | low | low | NA | NA | NA | NA | moderate | moderate | high |
| Silva, 2018[153] | low | moderate | low | low | NA | NA | NA | NA | NA | low | low | moderate |
| Sivan, 2012[154] | moderate | low | moderate | moderate | NA | NA | NA | NA | NA | high | moderate | high |
| Smit, 2005[155] | low | low | low | low | NA | NA | NA | NA | NA | low | low | low |
| Soubrane, 2005[88] | low | low | low | low | NA | NA | NA | NA | NA | low | low | low |
| Tarhini, 2009[157] | low | moderate | low | low | NA | NA | NA | NA | low | low | low | moderate |
| Tartour, 1996[158] | moderate | moderate | low | low | NA | NA | NA | NA | NA | low | low | moderate |
| Tas, 2005[159] | moderate | high | moderate | low | NA | NA | NA | NA | NA | low | low | high |
| Tawbi HA, 2022[160] | low | moderate | moderate | low | low | NA | NA | NA | NA | low | low | moderate |
| Tsutsumida, 2020[34] | low | low | low | low | low | NA | NA | NA | NA | unclear | low | low |
| Ugurel, 2001[161] | moderate | moderate | low | low | low | NA | NA | NA | NA | low | low | moderate |
| Umemura, 2017[162] | high | low | moderate | NA | NA | moderate | NA | NA | NA | high | moderate | high |
| Wilgenhof, 2013[163] | low | low | low | low | low | NA | NA | NA | NA | low | low | moderate |
| Wu, 2017[165] | moderate | moderate | moderate | low | NA | NA | NA | NA | NA | low | moderate | high |
| Wu, 2018[166] | high | unclear | moderate | moderate | NA | NA | NA | NA | NA | high | moderate | high |

**Abbreviations:** DFS: disease-free survival; DMFS: distant metastasis-free survival; DSS: disease-specific survival; OS: overall survival; PFS: progression-free survival; RFS: recurrence-free survival

O1- QUIPS tool

|  | Low | Moderate | High | N/A |
| --- | --- | --- | --- | --- |
| Study participation | 41.72% | 49.69% | 8.59% | 0% |
| Study attrition | 61.96% | 0% | 0% | 3.67% |
| Prognostic factor measurment | 77.3% | 22.4% | 0% | 0% |
| Outcome measurement | 93.3% | 6.7% | 0% | 0% |
| Study confounding | 51.53% | 28.83% | 4.9% | 14.72% |
| Statistical analysis reporting | 86.5% | 13.5% | 0% | 0% |
| Overall risk of bias | 12.88% | 59.5% | 27% | 0% |

## **Supplementary Figure S42**: Proportion of studies with low, high, or unclear risk of bias.

Abbreviations: QUIPS: Quality in Prognosis Studies

# **Publication Bias**

## **Supplementary Figure S43:** Funnel plot for studies of overall survival (OS) and baseline serum biomarker level associations in melanoma patients, independently from therapeutic agent.

**Abbreviations:** ALC: absolute lymphocyte count; ALP: alkaline phosphatase; ANC: absolute neutrophil count; A-TPO: anti-thyroid peroxidase antibody; CD37 activity: cluster of differentiation 37 activity; cfDNA: cell-free DNA; cHGF: circulating hepatocyte growth factor; CI: confidence interval; CRP: C-reactive protein; CTC: circulating tumor cells; ctDNA: circulating tumor DNA; dNLR: derived neutrophil-to-lymphocyte ratio; FT3: free triiodothyronine; FT4: free thyroxine; Gal-3: galectin-3; HGF: hepatocyte growth factor; HR: hazard ratio; IL-6: interleukin-6; IL-8: interleukin-8; Leu: leukocytes; LDH: lactate dehydrogenase; LMR: lymphocyte-to-monocyte ratio; Ly: lymphocytes; MART1-mRNA: melanoma antigen recognized by T cells 1 messenger RNA; MIA: melanoma inhibitory activity; miR: microRNA; mono: monocytes; Neu: neutrophils; NLR: neutrophil-to-lymphocyte ratio; PLR: platelet-to-lymphocyte ratio; S-100: S100 calcium-binding protein; SII index: systemic immune-inflammation index; TG: thyroglobulin; TSH: thyroid-stimulating hormone; VEGF: vascular endothelial growth factor; WBC: white blood cell count

## **Supplementary Figure S44:** Funnel plot for studies assessing overall survival (OS) and baseline serum biomarker level associations in melanoma patients based on therapeutic agent.

**Abbreviations:** ALC: absolute lymphocyte count; ALP: alkaline phosphatase; ANC: absolute neutrophil count; A-TPO: anti-thyroid peroxidase antibody; BRAF–MEK inhibitors: BRAF and MEK kinase inhibitors; cHGF: circulating hepatocyte growth factor; CI: confidence interval; CRP: C-reactive protein; CTC: circulating tumor cell; CTII: circulating type II interferon (IFN-γ); ctDNA: circulating tumor DNA; dNLR: derived neutrophil-to-lymphocyte ratio; FT3: free triiodothyronine; FT4: free thyroxine; Gal-3: galectin-3; HR: hazard ratio; IL-6: interleukin-6; IL-8: interleukin-8; LDH: lactate dehydrogenase; Leu: leukocytes; LMR: lymphocyte-to-monocyte ratio; Ly: lymphocytes; MART1-mRNA: melanoma antigen recognized by T cells 1 messenger RNA; MIA: melanoma inhibitory activity protein; miR: microRNA; Mono: monocytes; Neu: neutrophils; NLR: neutrophil-to-lymphocyte ratio; p-proteasome: circulating 20S proteasome; PLR: platelet-to-lymphocyte ratio; S100: S100 calcium-binding protein; SII: systemic immune-inflammation index; TG: thyroglobulin; TK activity: thymidine kinase activity; TSH: thyroid-stimulating hormone; VEGF: vascular endothelial growth factor; WBC: white blood cell count

## **Supplementary Figure S45:** Funnel plot for studies assessing overall survival (OS) and baseline lactate dehydrogenase (LDH) level associations in stage III-IV stage melanoma patients.

## **Supplementary Figure S46:** Funnel plot for studies assessing overall survival (OS) and baseline serum lactate dehydrogenase (LDH) level associations subgrouped based on therapeutic agent and disease stage in melanoma patients.

## **Supplementary Figure S47:** Funnel plot for studies assessing overall survival (OS) and baseline circulating tumor DNA (ctDNA) level associations in stage III-IV stage melanoma patients.

## **Supplementary Figure S48:** Funnel plot for studies assessing overall survival (OS) and baseline serum circulating tumor DNA (ctDNA) level associations subgrouped based on therapeutic agent and disease stage in melanoma patients.

## **Supplementary Figure S49:** Funnel plot for studies assessing overall survival (OS) and baseline S100 level associations in stage III-IV stage melanoma patients.

## **Supplementary Figure S50:** Funnel plot for studies assessing overall survival (OS) and baseline serum S100 level associations subgrouped based on therapeutic agent and disease stage in melanoma patients.

## **Supplementary Figure S51:** Funnel plot for studies assessing overall survival (OS) and baseline neutrophil-to-lymphocyte ratio (NLR) associations in stage III-IV stage melanoma patients.

## **Supplementary Figure S52:** Funnel plot for studies assessing overall survival (OS) and baseline neutrophil-to-lymphocyte ratio (NLR) associations subgrouped based on therapeutic agent and disease stage in melanoma patients.

## **Supplementary Figure S53:** Funnel plot for studies assessing overall survival (OS) and baseline interleukin-6 (IL-6) level associations in stage III-IV stage melanoma patients.

## **Supplementary Figure S54:** Funnel plot for studies assessing overall survival (OS) and baseline serum interleukin-6 (IL-6) level associations subgrouped based on therapeutic agent and disease stage in melanoma patients.

## **Supplementary Figure S55:** Funnel plot for studies assessing overall survival (OS) and baseline C-reactive protein (CRP) level associations in stage III-IV stage melanoma patients.

## **Supplementary Figure S56:** Funnel plot for studies assessing overall survival (OS) and baseline serum C-reactive protein (CRP) level associations subgrouped based on therapeutic agent and disease stage in melanoma patients.

## **Supplementary Figure S57:** Funnel plot for studies assessing overall survival (OS) and serum biomarker levels association in melanoma patients during follow-up.

**Abbreviations:** ctDNA: circulating tumor DNA; LDH: lactate dehydrogenase; LMR: lymphocyte-to-monocyte ratio; NLR: neutrophil-to-lymphocyte ratio; PLR: platelet-to-lymphocyte ratio

## **Supplementary Figure S58:** Funnel plot for studies assessing progression-free (PFS) and baseline serum biomarker level associations in melanoma patients, regardless of therapeutic agent.

**Abbreviations:** ALC: absolute lymphocyte count; ANC: absolute neutrophil count; anti-TPO: anti-thyroid peroxidase antibody; CD37 activity: cluster of differentiation 37 activity; cfDNA: cell-free DNA; CI: confidence interval; CRP: C-reactive protein; CTC: circulating tumor cell; ctDNA: circulating tumor DNA; dNLR: derived neutrophil-to-lymphocyte ratio; HGF: hepatocyte growth factor; HR: hazard ratio; HSP90α: heat shock protein 90 alpha; IL-6: interleukin-6; IL-18: interleukin-18; log: logarithm; LMR: lymphocyte-to-monocyte ratio; Ly: lymphocytes; MIA: melanoma inhibitory activity; Neu: neutrophils; p-proteasome: circulating 20S proteasome; PLR: platelet-to-lymphocyte ratio; S100: S100 calcium-binding protein; SE: standard error; SII index: systemic immune-inflammation index; TK activity: thymidine kinase activity; WBC: white blood cell count

## **Supplementary Figure S59:** Funnel plot for studies assessing progression-free survival (PFS) and baseline serum biomarker level associations in melanoma patients subgrouped based on therapeutic agent.

**Abbreviations:** ALC: absolute lymphocyte count; ANC: absolute neutrophil count; anti-TPO: anti-thyroid peroxidase antibody; CD37 activity: cluster of differentiation 37 activity; CI: confidence interval; CRP: C-reactive protein; CTC: circulating tumor cell; ctDNA: circulating tumor DNA; dNLR: derived neutrophil-to-lymphocyte ratio; HR: hazard ratio; IL-6: interleukin-6; IL-18: interleukin-18; LDH: lactate dehydrogenase; log: logarithm; Ly: lymphocytes; Neu: neutrophils; NLR: neutrophil-to-lymphocyte ratio; S100: S100 calcium-binding protein; SE: standard error; TK activity: thymidine kinase activity; WBC: white blood cell count.

## **Supplementary Figure S60:** Funnel plot for studies assessing progression-free survival (PFS) and baseline lactate dehydrogenase (LDH) level associations in stage III-IV stage melanoma patients.

## **Supplementary Figure S61**: Funnel plot for studies assessing progression-free survival (PFS) and baseline serum lactate dehydrogenase (LDH) level associations, subgrouped based on therapeutic agent and disease stage in melanoma patients.

## **Supplementary Figure S62:** Funnel plot for studies assessing progression-free survival (PFS) and baseline circulating tumor DNA (ctDNA) level associations in stage III-IV stage melanoma patients.

## **Supplementary Figure S63:** Funnel plot for studies assessing progression-free survival (PFS) and baseline serum circulating tumor DNA (ctDNA) level associations subgrouped based on therapeutic agent and disease stage in melanoma patients.

## **Supplementary Figure S64**: Funnel plot for studies assessing progression-free survival (PFS) and baseline neutrophil-to-lymphocyte ratio (NLR) associations in stage III-IV stage melanoma patients.

## **Supplementary Figure S65:** Funnel plot for studies assessing progression-free survival (PFS) and baseline neutrophil-to-lymphocyte ratio (NLR) associations subgrouped based on therapeutic agent and disease stage in melanoma patients.

## **Supplementary Figure S66:** Funnel plot for studies assessing progression-free survival (PFS) and serum biomarker levels association in melanoma patients during follow-up.

Abbreviations: ctDNA: circulating tumor DNA; LDH: lactate dehydrogenase; LMR: lymphocyte-to-monocyte ratio; NLR: neutrophil-to-lymphocyte ratio; PLR: platelet-to-lymphocyte ratio; S100: S100 calcium-binding protein

## **Supplementary Figure S67:** Funnel plot for studies assessing distant metastasis disease-free survival (DM-DFS) and serum biomarker levels association in melanoma patients during follow-up.

**Abbreviations:** ctDNA: circulating tumor DNA; S100: S100 calcium-binding protein

## **Supplementary Figure S68:** Funnel plot for studies assessing disease-specific survival (DSS) and baseline serum biomarker level associations in melanoma patients, regardless of therapeutic agent.

**Abbreviations:** CRP: C-reactive protein; ctDNA: circulating tumor DNA; LDH: lactate dehydrogenase; S100: S100 calcium-binding protein

## **Supplementary Figure S69:** Funnel plot for studies assessing recurrence-free survival (RFS) and baseline serum biomarker level associations in melanoma patients, regardless of therapeutic agent.

Abbreviations: CRP: C-reactive protein; CTC: circulating tumor cell; ctDNA: circulating tumor DNA; LDH: lactate dehydrogenase; LMR: lymphocyte-to-monocyte ratio; NLR: neutrophil-to-lymphocyte ratio; PLR: platelet-to-lymphocyte ratio

# **Multivariate-model diagnostics**

## **Supplementary Figure S70:** Fitted versus observed values from the multivariate meta-analysis model assessing baseline LDH levels and OS,demonstrating adequate model fit.

## **Supplementary Figure S71:** Funnel plot of the multivariate meta-analysis model for baseline LDH and OS.

## **Supplementary Figure S72:** Normal Q–Q plot of standardized residuals for the multivariate meta-analysis of baseline LDH and OS, indicating no major deviation from normality.

## **Supplementary Figure S73:** Study-level effect estimates from the multivariate random-effects meta-analysis model for baseline LDH and OS.

## **Supplementary Figure S74:** Fitted versus observed values from the multivariate meta-analysis model assessing baseline IL-6 levels and OS, demonstrating adequate model fit.

## **Supplementary Figure S75:** Funnel plot of the multivariate meta-analysis model for baseline IL-6 and OS.

## **Supplementary Figure S76:** Normal Q–Q plot of standardized residuals for the multivariate meta-analysis of baseline IL-6 and OS, indicating no major deviation from normality.

## **Supplementary Figure S77:** Study-level effect estimates from the multivariate random-effects meta-analysis model for baseline IL-6 and OS.

## **Supplementary Figure S78:** Funnel plot of the multivariate meta-analysis model for baseline NLR and OS.

## **Supplementary Figure S79:** Normal Q–Q plot of standardized residuals for the multivariate meta-analysis of baseline NLR and OS, indicating no major deviation from normality.

## **Supplementary Figure S80:** Study-level effect estimates from the multivariate random-effects meta-analysis model for baseline NLR and OS.

## **Supplementary Figure S81:** Funnel plot of the multivariate meta-analysis model for baseline ctDNA and OS.

## **Supplementary Figure S82:** Funnel plot of the multivariate meta-analysis model for baseline S100B and OS.

## **Supplementary Figure S83:** Fitted versus observed values from the multivariate meta-analysis model assessing baseline CRP levels and OS, demonstrating adequate model fit.

## **Supplementary Figure S84:** Funnel plot of the multivariate meta-analysis model for baseline CRP and OS.

## **Supplementary Figure S85:** Normal Q–Q plot of standardized residuals for the multivariate meta-analysis of baseline CRP and OS, indicating no major deviation from normality.

## **Supplementary Figure S86:** Study-level effect estimates from the multivariate random-effects meta-analysis model for baseline CRP and OS.

## **Supplementary Figure S87:** Funnel plot of the multivariate meta-analysis model for baseline LDH and PFS.

## **Supplementary Figure S88:** Normal Q–Q plot of standardized residuals for the multivariate meta-analysis of baseline LDH and PFS, indicating no major deviation from normality.

## **Supplementary Figure S89:** Funnel plot of the multivariate meta-analysis model for baseline ctDNA and PFS.

## **Supplementary Figure S90:** Funnel plot of the multivariate meta-analysis model for baseline NLR and PFS.

## **Supplementary Figure S91:** Funnel plot of the multivariate meta-analysis model for baseline CRP and PFS.

## **Supplementary Figure S92:** Funnel plot of the multivariate meta-analysis model for baseline LDH and DSS.

## **Supplementary Figure S93:** Funnel plot of the multivariate meta-analysis model for baseline S100B and DSS.

# **SupplementaryReferences**

1	Aamdal, E. et al. Ipilimumab in a real-world population: A prospective Phase IV trial with long-term follow-up. Int J Cancer **150**, 100-111 (2022). <https://doi.org/doi:10.1002/ijc.33768>

2	Abu-Sbeih, H. et al. Immune checkpoint inhibitor-induced colitis as a predictor of survival in metastatic melanoma. Cancer Immunol Immunother **68**, 553-561 (2019). <https://doi.org/10.1007/s00262-019-02303-1>

3	Amaral, T. et al. Combined immunotherapy with nivolumab and ipilimumab with and without local therapy in patients with melanoma brain metastasis: a DeCOG* study in 380 patients. J Immunother Cancer **8** (2020). <https://doi.org/doi:10.1136/jitc-2019-000333>

4	Laino, A. S. et al. Serum interleukin-6 and C-reactive protein are associated with survival in melanoma patients receiving immune checkpoint inhibition. J Immunother Cancer **8** (2020). <https://doi.org/10.1136/jitc-2020-000842>

5	Arheden, A. et al. Real-world data on PD-1 inhibitor therapy in metastatic melanoma. Acta Oncol **58**, 962-966 (2019). <https://doi.org/doi:10.1080/0284186x.2019.1620966>

6	Bhatia, A. et al. MRI radiomic features are associated with survival in melanoma brain metastases treated with immune checkpoint inhibitors. Neuro Oncol **21**, 1578-1586 (2019). <https://doi.org/doi:10.1093/neuonc/noz141>

7	Capone, M. et al. Baseline neutrophil-to-lymphocyte ratio (NLR) and derived NLR could predict overall survival in patients with advanced melanoma treated with nivolumab. J Immunother Cancer **6**, 74 (2018). <https://doi.org/10.1186/s40425-018-0383-1>

8	Damuzzo, V. et al. Clinical implication of tumor-associated and immunological parameters in melanoma patients treated with ipilimumab. Oncoimmunology **5**, e1249559 (2016). <https://doi.org/doi:10.1080/2162402x.2016.1249559>

9	Eroglu, Z. et al. Circulating tumor DNA-based molecular residual disease detection for treatment monitoring in advanced melanoma patients. Cancer **129**, 1723-1734 (2023). <https://doi.org/doi:10.1002/cncr.34716>

10	Gray, E. S. et al. Circulating tumor DNA to monitor treatment response and detect acquired resistance in patients with metastatic melanoma. Oncotarget **6**, 42008-42018 (2015). <https://doi.org/doi:10.18632/oncotarget.5788>

11	Heidelberger, V. et al. Clinical parameters associated with anti-programmed death-1 (PD-1) inhibitors-induced tumor response in melanoma patients. Invest New Drugs **35**, 842-847 (2017). <https://doi.org/doi:10.1007/s10637-017-0476-6>

12	Herbreteau, G. et al. Quantitative monitoring of circulating tumor DNA predicts response of cutaneous metastatic melanoma to anti-PD1 immunotherapy. Oncotarget **9**, 25265-25276 (2018). <https://doi.org/doi:10.18632/oncotarget.25404>

13	Hoejberg, L. et al. Serum interleukin-6 as a prognostic biomarker in patients with metastatic melanoma. Melanoma Res **22**, 287-293 (2012). <https://doi.org/doi:10.1097/CMR.0b013e3283550aa5>

14	Kelderman, S. et al. Lactate dehydrogenase as a selection criterion for ipilimumab treatment in metastatic melanoma. Cancer Immunol Immunother **63**, 449-458 (2014). <https://doi.org/doi:10.1007/s00262-014-1528-9>

15	Khoja, L. et al. The full blood count as a biomarker of outcome and toxicity in ipilimumab-treated cutaneous metastatic melanoma. Cancer Med **5**, 2792-2799 (2016). <https://doi.org/doi:10.1002/cam4.878>

16	Koguchi, Y. et al. Serum Immunoregulatory Proteins as Predictors of Overall Survival of Metastatic Melanoma Patients Treated with Ipilimumab. Cancer research **75**, 5084‐5092 (2015). <https://doi.org/doi:10.1158/0008-5472.CAN-15-2303>

17	Krakowski, I. et al. Impact of modern systemic therapies and clinical markers on treatment outcome for metastatic melanoma in a real-world setting. J Eur Acad Dermatol Venereol **35**, 105-115 (2021). <https://doi.org/doi:10.1111/jdv.16678>

18	Lee, J. H. et al. Circulating tumour DNA predicts response to anti-PD1 antibodies in metastatic melanoma. Ann Oncol **28**, 1130-1136 (2017). <https://doi.org/doi:10.1093/annonc/mdx026>

19	Lee, J. H. et al. Longitudinal Monitoring of ctDNA in Patients with Melanoma and Brain Metastases Treated with Immune Checkpoint Inhibitors. Clin Cancer Res **26**, 4064-4071 (2020). <https://doi.org/doi:10.1158/1078-0432.Ccr-19-3926>

20	Machiraju, D. et al. Rapid disease progression on immune checkpoint inhibitors in young patients with stage IV melanoma. Front Med (Lausanne) **10**, 1117816 (2023). <https://doi.org/doi:10.3389/fmed.2023.1117816>

21	Mallardo, D. et al. CD39 and LDHA affects the prognostic role of NLR in metastatic melanoma patients treated with immunotherapy. J Transl Med **21**, 610 (2023). <https://doi.org/doi:10.1186/s12967-023-04419-6>

22	Musaelyan, A. A. et al. Inflammatory and autoimmune predictive markers of response to anti-PD-1/PD-L1 therapy in NSCLC and melanoma. Exp Ther Med **24**, 557 (2022). <https://doi.org/doi:10.3892/etm.2022.11495>

23	Pedersen, J. G. et al. Inflammatory Cytokines and ctDNA Are Biomarkers for Progression in Advanced-Stage Melanoma Patients Receiving Checkpoint Inhibitors. Cancers (Basel) **12** (2020). <https://doi.org/doi:10.3390/cancers12061414>

24	Rogala, P. et al. Long Term Results and Prognostic Biomarkers for Anti-PD1 Immunotherapy Used after BRAFi/MEKi Combination in Advanced Cutaneous Melanoma Patients. Cancers (Basel) **14** (2022). <https://doi.org/doi:10.3390/cancers14092123>

25	Rose, A. A. N. et al. Biologic subtypes of melanoma predict survival benefit of combination anti-PD1+anti-CTLA4 immune checkpoint inhibitors versus anti-PD1 monotherapy. J Immunother Cancer **9** (2021). <https://doi.org/doi:10.1136/jitc-2020-001642>

26	Rossi, N. et al. Circulating inflammatory proteins associate with response to immune checkpoint inhibition therapy in patients with advanced melanoma. EBioMedicine **83**, 104235 (2022). <https://doi.org/doi:10.1016/j.ebiom.2022.104235>

27	Rosner, S. et al. Peripheral blood clinical laboratory variables associated with outcomes following combination nivolumab and ipilimumab immunotherapy in melanoma. Cancer Med **7**, 690-697 (2018). <https://doi.org/10.1002/cam4.1356>

28	Schmidt, H. et al. Elevated neutrophil and monocyte counts in peripheral blood are associated with poor survival in patients with metastatic melanoma: a prognostic model. Br J Cancer **93**, 273-278 (2005). <https://doi.org/doi:10.1038/sj.bjc.6602702>

29	Schraag, A. et al. Baseline clinical and imaging predictors of treatment response and overall survival of patients with metastatic melanoma undergoing immunotherapy. Eur J Radiol **121**, 108688 (2019). <https://doi.org/doi:10.1016/j.ejrad.2019.108688>

30	Seremet, T. et al. Undetectable circulating tumor DNA (ctDNA) levels correlate with favorable outcome in metastatic melanoma patients treated with anti-PD1 therapy. J Transl Med **17**, 303 (2019). <https://doi.org/doi:10.1186/s12967-019-2051-8>

31	Simetić, L., Blažičević, K., Međugorac, K., Golčić, M. & Herceg, D. Relative Change in S100 as a Biomarker of Survival in Patients With Metastatic Melanoma Treated With Pembrolizumab. Anticancer Res **40**, 2157-2163 (2020). <https://doi.org/10.21873/anticanres.14175>

32	Costa Svedman, F. et al. Plasma Thymidine Kinase Activity as a Novel Biomarker in Metastatic Melanoma Patients Treated with Immune Checkpoint Inhibitors. Cancers (Basel) **14** (2022). <https://doi.org/10.3390/cancers14030702>

33	Trommer, M. et al. Oncologic Outcome and Immune Responses of Radiotherapy with Anti-PD-1 Treatment for Brain Metastases Regarding Timing and Benefiting Subgroups. Cancers (Basel) **14** (2022). <https://doi.org/doi:10.3390/cancers14051240>

34	Tsutsumida, A. et al. Japanese real-world study of sequential nivolumab and ipilimumab treament in melanoma. J Dermatol **46**, 947-955 (2019). <https://doi.org/doi:10.1111/1346-8138.15073>

35	Turiello, R. et al. Serum CD73 is a prognostic factor in patients with metastatic melanoma and is associated with response to anti-PD-1 therapy. J Immunother Cancer **8** (2020). <https://doi.org/doi:10.1136/jitc-2020-001689>

36	Váraljai, R. et al. Application of Circulating Cell-Free Tumor DNA Profiles for Therapeutic Monitoring and Outcome Prediction in Genetically Heterogeneous Metastatic Melanoma. JCO Precis Oncol **3** (2020). <https://doi.org/doi:10.1200/po.18.00229>

37	Vila, C. M. et al. Exploratory analysis of clinical benefit of ipilimumab and nivolumab treatment in patients with metastatic melanoma from a single institution. Clin Transl Oncol **24**, 319-330 (2022). <https://doi.org/10.1007/s12094-021-02692-9>

38	Zaragoza, J. et al. High neutrophil to lymphocyte ratio measured before starting ipilimumab treatment is associated with reduced overall survival in patients with melanoma. Br J Dermatol **174**, 146-151 (2016). <https://doi.org/10.1111/bjd.14155>

39	Zhao, J. et al. Nomogram for predicting prognosis of patients with metastatic melanoma after immunotherapy: A Chinese population-based analysis. Front Immunol **13**, 1083840 (2022). <https://doi.org/doi:10.3389/fimmu.2022.1083840>

40	Cocorocchio, E. et al. Baseline neutrophil-to-lymphocyte ratio (NLR) is associated with outcome of patients treated with BRAF inhibitors. Clin Transl Oncol **22**, 1818-1824 (2020). <https://doi.org/doi:10.1007/s12094-020-02320-y>

41	Czirbesz, K. et al. Efficacy of Vemurafenib Treatment in 43 Metastatic Melanoma Patients with BRAF Mutation. Single-Institute Retrospective Analysis, Early Real-Life Survival Data. Pathol Oncol Res **25**, 45-50 (2019). <https://doi.org/doi:10.1007/s12253-017-0324-1>

42	Depenni, R. et al. Dabrafenib-trametinib combination in 'field-practice': an Italian experience. Future Oncol **14**, 2045-2052 (2018). <https://doi.org/doi:10.2217/fon-2017-0714>

43	Dutriaux, C. et al. An open label, non-randomised, phase IIIb study of trametinib in combination with dabrafenib in patients with unresectable (stage III) or distant metastatic (stage IV) BRAF V600-mutant melanoma: A subgroup analysis of patients with brain metastases. Eur J Cancer **175**, 254-262 (2022). <https://doi.org/doi:10.1016/j.ejca.2022.07.035>

44	Finon, A. et al. A high neutrophil to lymphocyte ratio prior to BRAF inhibitor treatment is a predictor of poor progression-free survival in patients with metastatic melanoma. Eur J Dermatol **28**, 38-43 (2018). <https://doi.org/doi:10.1684/ejd.2017.3167>

45	Garlan, F. et al. Circulating Tumor DNA Measurement by Picoliter Droplet-Based Digital PCR and Vemurafenib Plasma Concentrations in Patients with Advanced BRAF-Mutated Melanoma. Target Oncol **12**, 365-371 (2017). <https://doi.org/doi:10.1007/s11523-017-0491-8>

46	Gassenmaier, M. et al. Serum S100B and LDH at Baseline and During Therapy Predict the Outcome of Metastatic Melanoma Patients Treated with BRAF Inhibitors. Target Oncol **16**, 197-205 (2021). <https://doi.org/doi:10.1007/s11523-021-00792-8>

47	Kozak, K. et al. Cell-free DNA BRAF V600E measurements during BRAF inhibitor therapy of metastatic melanoma: long-term analysis. Tumori, 300891619900928 (2020). <https://doi.org/doi:10.1177/0300891619900928>

48	Long, G. V. et al. Overall Survival and Durable Responses in Patients With BRAF V600-Mutant Metastatic Melanoma Receiving Dabrafenib Combined With Trametinib. Journal of clinical oncology **34**, 871‐878 (2016). <https://doi.org/doi:10.1200/JCO.2015.62.9345>

49	Lu, W. et al. Elevated Levels of BRAF(V600) Mutant Circulating Tumor DNA and Circulating Hepatocyte Growth Factor Are Associated With Poor Prognosis in Patients With Metastatic Melanoma. JCO Precis Oncol **2**, 1-17 (2018). <https://doi.org/doi:10.1200/po.17.00168>

50	Ruggiero, C. F. et al. Identification of a miRNA-based non-invasive predictive biomarker of response to target therapy in BRAF-mutant melanoma. Theranostics **12**, 7420-7430 (2022). <https://doi.org/doi:10.7150/thno.77761>

51	Ugurel, S. et al. A multicenter DeCOG study on predictors of vemurafenib therapy outcome in melanoma: pretreatment impacts survival. Ann Oncol **26**, 573-582 (2015). <https://doi.org/doi:10.1093/annonc/mdu573>

52	Saiag, P. et al. Efficacy, safety and factors associated with disease progression in patients with unresectable (stage III) or distant metastatic (stage IV) BRAF V600-mutant melanoma: An open label, non-randomized, phase IIIb study of trametinib in combination with dabrafenib. Eur J Cancer **154**, 57-65 (2021). <https://doi.org/10.1016/j.ejca.2021.05.031>

53	Syeda, M. M. et al. Circulating tumour DNA in patients with advanced melanoma treated with dabrafenib or dabrafenib plus trametinib: a clinical validation study. The lancet. Oncology **22**, 370‐380 (2021). <https://doi.org/doi:10.1016/S1470-2045(20)30726-9>

54	Allen, B. J. et al. Analysis of patient survival in a Phase I trial of systemic targeted α-therapy for metastatic melanoma. Immunotherapy **3**, 1041-1050 (2011). <https://doi.org/doi:10.2217/imt.11.97>

55	Aoude, L. G. et al. Circulating Tumor DNA: A Promising Biomarker for Predicting Recurrence in Patients with BRAF-Negative Melanoma. J Mol Diagn **25**, 771-781 (2023). <https://doi.org/10.1016/j.jmoldx.2023.06.014>

56	Awada, G. et al. A Comprehensive Analysis of Baseline Clinical Characteristics and Biomarkers Associated with Outcome in Advanced Melanoma Patients Treated with Pembrolizumab. Cancers (Basel) **13** (2021). <https://doi.org/doi:10.3390/cancers13020168>

57	Boerlin, A. et al. The Prognostic Value of a Single, Randomly Timed Circulating Tumor DNA Measurement in Patients with Metastatic Melanoma. Cancers (Basel) **14** (2022). <https://doi.org/doi:10.3390/cancers14174158>

58	Braune, J. et al. Circulating Tumor DNA Allows Early Treatment Monitoring in BRAF- and NRAS-Mutant Malignant Melanoma. JCO Precis Oncol **4**, 20-31 (2020). <https://doi.org/doi:10.1200/po.19.00174>

59	Weide, B. et al. Serum S100B, lactate dehydrogenase and brain metastasis are prognostic factors in patients with distant melanoma metastasis and systemic therapy. PLoS One **8**, e81624 (2013). <https://doi.org/doi:10.1371/journal.pone.0081624>

60	Cybulska-Stopa, B. et al. Real-world treatment practice in patients with advanced melanoma. Contemp Oncol (Pozn) **24**, 118-124 (2020). <https://doi.org/10.5114/wo.2020.97607>

61	Cananzi, F. C., Dalgleish, A. & Mudan, S. Surgical management of intraabdominal metastases from melanoma: role of the neutrophil to lymphocyte ratio as a potential prognostic factor. World J Surg **38**, 1542-1550 (2014). <https://doi.org/doi:10.1007/s00268-013-2418-6>

62	Chiarion-Sileni, V. et al. Quality of life evaluation in a randomised trial of chemotherapy versus bio-chemotherapy in advanced melanoma patients. Eur J Cancer **39**, 1577-1585 (2003). <https://doi.org/10.1016/s0959-8049(03)00372-1>

63	Desai, A. D. et al. An analysis of lactate dehydrogenase (LDH) levels in advanced stage IV melanoma of the skin: prognostic capabilities and demographic variability. Arch Dermatol Res **315**, 799-806 (2023). <https://doi.org/doi:10.1007/s00403-022-02425-0>

64	Desch, A., Gebhardt, C., Utikal, J. & Schneider, S. W. D-dimers in malignant melanoma: Association with prognosis and dynamic variation in disease progress. Int J Cancer **140**, 914-921 (2017). <https://doi.org/doi:10.1002/ijc.30498>

65	Eton, O. et al. Sequential biochemotherapy versus chemotherapy for metastatic melanoma: results from a phase III randomized trial. J Clin Oncol **20**, 2045-2052 (2002). <https://doi.org/10.1200/jco.2002.07.044>

66	Fang, S. et al. C-reactive protein as a marker of melanoma progression. J Clin Oncol **33**, 1389-1396 (2015). <https://doi.org/doi:10.1200/jco.2014.58.0209>

67	Forthun, R. B. et al. ctDNA detected by ddPCR reveals changes in tumour load in metastatic malignant melanoma treated with bevacizumab. Sci Rep **9**, 17471 (2019). <https://doi.org/doi:10.1038/s41598-019-53917-5>

68	Gonzalez-Cao, M. et al. Early evolution of BRAFV600 status in the blood of melanoma patients correlates with clinical outcome and identifies patients refractory to therapy. Melanoma Res **28**, 195-203 (2018). <https://doi.org/doi:10.1097/cmr.0000000000000432>

69	Gray, M. R. et al. Metastatic melanoma: lactate dehydrogenase levels and CT imaging findings of tumor devascularization allow accurate prediction of survival in patients treated with bevacizumab. Radiology **270**, 425‐434 (2014). <https://doi.org/doi:10.1148/radiol.13130776>

70	Henry, L. et al. Clinical use of p-proteasome in discriminating metastatic melanoma patients: comparative study with LDH, MIA and S100B protein. Int J Cancer **133**, 142-148 (2013). <https://doi.org/doi:10.1002/ijc.27991>

71	Hügel, R. et al. Serum levels of hepatocyte growth factor as a potential tumor marker in patients with malignant melanoma. Melanoma Res **26**, 354-360 (2016). <https://doi.org/doi:10.1097/cmr.0000000000000269>

72	Ibrahim, A. M. et al. Imaging Intensity and Survival Outcomes in High-Risk Resected Melanoma Treated by Systemic Therapy at Recurrence. Ann Surg Oncol **27**, 3683-3691 (2020). <https://doi.org/doi:10.1245/s10434-020-08407-8>

73	Internò, V. et al. Melanoma Brain Metastases: A Retrospective Analysis of Prognostic Factors and Efficacy of Multimodal Therapies. Cancers (Basel) **15** (2023). <https://doi.org/doi:10.3390/cancers15051542>

74	Kopecky, J. et al. Prognostic value of blood cell count-derived ratios in BRAF-mutated metastatic melanoma. Biomed Pap Med Fac Univ Palacky Olomouc Czech Repub **166**, 393-404 (2022). <https://doi.org/doi:10.5507/bp.2021.053>

75	Wevers, K. P. et al. S-100B: a stronger prognostic biomarker than LDH in stage IIIB-C melanoma. Ann Surg Oncol **20**, 2772-2779 (2013). <https://doi.org/10.1245/s10434-013-2949-y>

76	Lee, R. J. et al. Circulating tumor DNA predicts survival in patients with resected high-risk stage II/III melanoma. Ann Oncol **29**, 490-496 (2018). <https://doi.org/doi:10.1093/annonc/mdx717>

77	Marcus, D. M. et al. Prognostic factors for overall survival after radiosurgery for brain metastases from melanoma. Am J Clin Oncol **37**, 580-584 (2014). <https://doi.org/doi:10.1097/COC.0b013e318280d7be>

78	Meckbach, D. et al. BRAF-V600 mutations have no prognostic impact in stage IV melanoma patients treated with monochemotherapy. PLoS One **9**, e89218 (2014). <https://doi.org/doi:10.1371/journal.pone.0089218>

79	Moser, J. C. et al. Real-world survival of patients with advanced BRAF V600 mutated melanoma treated with front-line BRAF/MEK inhibitors, anti-PD-1 antibodies, or nivolumab/ipilimumab. Cancer Med **8**, 7637-7643 (2019). <https://doi.org/doi:10.1002/cam4.2625>

80	Neuman, H. B. et al. Stage-IV melanoma and pulmonary metastases: factors predictive of survival. Ann Surg Oncol **14**, 2847-2853 (2007). <https://doi.org/doi:10.1245/s10434-007-9448-y>

81	Partl, R., Richtig, E., Avian, A., Berghold, A. & Kapp, K. S. Karnofsky performance status and lactate dehydrogenase predict the benefit of palliative whole-brain irradiation in patients with advanced intra- and extracranial metastases from malignant melanoma. Int J Radiat Oncol Biol Phys **85**, 662-666 (2013). <https://doi.org/doi:10.1016/j.ijrobp.2012.06.009>

82	Partl, R. et al. KPS/LDH index: a simple tool for identifying patients with metastatic melanoma who are unlikely to benefit from palliative whole brain radiotherapy. Support Care Cancer **24**, 523-528 (2016). <https://doi.org/doi:10.1007/s00520-015-2793-7>

83	Reinert, C. P. et al. Clinical and prognostic value of tumor volumetric parameters in melanoma patients undergoing (18)F-FDG-PET/CT: a comparison with serologic markers of tumor burden and inflammation. Cancer Imaging **20**, 44 (2020). <https://doi.org/doi:10.1186/s40644-020-00322-1>

84	Kruijff, S. et al. The value of pre operative S-100B and SUV in clinically stage III melanoma patients undergoing therapeutic lymph node dissection. Eur J Surg Oncol **37**, 225-232 (2011). <https://doi.org/10.1016/j.ejso.2010.12.013>

85	Keilholz, U., Scheibenbogen, C., Sommer, M., Pritsch, M. & Geuke, A. M. Prognostic factors for response and survival in patients with metastatic melanoma receiving immunotherapy. Melanoma research **6**, 173‐178 (1996). <https://doi.org/doi:10.1097/00008390-199604000-00013>

86	Valpione, S. et al. Plasma total cell-free DNA (cfDNA) is a surrogate biomarker for tumour burden and a prognostic biomarker for survival in metastatic melanoma patients. Eur J Cancer **88**, 1-9 (2018). <https://doi.org/doi:10.1016/j.ejca.2017.10.029>

87	Schmidt, H., Sorensen, B. S., Fode, K., Nexo, E. & von der Maase, H. Tyrosinase messenger RNA in peripheral blood is related to poor survival in patients with metastatic melanoma following interleukin-2-based immunotherapy. Melanoma Res **15**, 409-416 (2005). <https://doi.org/10.1097/00008390-200510000-00009>

88	Soubrane, C., Rixe, O., Meric, J. B., Khayat, D. & Mouawad, R. Pretreatment serum interleukin-6 concentration as a prognostic factor of overall survival in metastatic malignant melanoma patients treated with biochemotherapy: a retrospective study. Melanoma Res **15**, 199-204 (2005). <https://doi.org/doi:10.1097/00008390-200506000-00009>

89	Marczynski, G. T., Laus, A. C., Dos Reis, M. B., Reis, R. M. & Vazquez, V. L. Circulating tumor DNA (ctDNA) detection is associated with shorter progression-free survival in advanced melanoma patients. Sci Rep **10**, 18682 (2020). <https://doi.org/10.1038/s41598-020-75792-1>

90	Tolmeijer, S. H. et al. Plasma BRAF Mutation Detection for the Diagnostic and Monitoring Trajectory of Patients with LDH-High Stage IV Melanoma. Cancers (Basel) **13** (2021). <https://doi.org/doi:10.3390/cancers13153913>

91	Váraljai, R. et al. The predictive and prognostic significance of cell-free DNA concentration in melanoma. J Eur Acad Dermatol Venereol **35**, 387-395 (2021). <https://doi.org/doi:10.1111/jdv.16766>

92	Vereecken, P. et al. Evaluation of the prognostic significance of serum galectin-3 in American Joint Committee on Cancer stage III and stage IV melanoma patients. Melanoma Res **19**, 316-320 (2009). <https://doi.org/doi:10.1097/CMR.0b013e32832ec001>

93	Yu, J. et al. Systemic Immune-Inflammation Index and Circulating T-Cell Immune Index Predict Outcomes in High-Risk Acral Melanoma Patients Treated with High-Dose Interferon. Transl Oncol **10**, 719-725 (2017). <https://doi.org/doi:10.1016/j.tranon.2017.06.004>

94	van Zeijl, M. C. T. et al. Real-world outcomes of advanced melanoma patients not represented in phase III trials. Int J Cancer **147**, 3461-3470 (2020). <https://doi.org/10.1002/ijc.33162>

95	Zhang, T. et al. Diagnostic and prognostic value of heat shock protein 90α in malignant melanoma. Melanoma Res **31**, 152-161 (2021). <https://doi.org/doi:10.1097/cmr.0000000000000716>

96	Porter, G. A. et al. Significance of plasma cytokine levels in melanoma patients with histologically negative sentinel lymph nodes. Ann Surg Oncol **8**, 116-122 (2001). <https://doi.org/doi:10.1007/s10434-001-0116-3>

97	Aglietta, M. et al. Outcomes in patients with BRAF(V600)-mutated melanoma and brain metastases at baseline treated with dabrafenib plus trametinib. Tumori, 3008916231179251 (2023). <https://doi.org/doi:10.1177/03008916231179251>

98	Weide, B. et al. Serum markers lactate dehydrogenase and S100B predict independently disease outcome in melanoma patients with distant metastasis. Br J Cancer **107**, 422-428 (2012). <https://doi.org/doi:10.1038/bjc.2012.306>

99	Bolander, A. et al. Serological and immunohistochemical analysis of S100 and new derivatives as markers for prognosis in patients with malignant melanoma. Melanoma Res **18**, 412-419 (2008). <https://doi.org/doi:10.1097/CMR.0b013e328315c690>

100	Cao, M. G. et al. Melanoma inhibiting activity protein (MIA), beta-2 microglobulin and lactate dehydrogenase (LDH) in metastatic melanoma. Anticancer Res **27**, 595-599 (2007).

101	Chung, M. H. et al. Serum TA90 immune complex assay can predict outcome after resection of thick (≥4 mm) primary melanoma and sentinel lymphadenectomy. Annals of Surgical Oncology **9**, 120-126 (2002). <https://doi.org/doi:10.1245/aso.2002.9.2.120>

102	Corrie, P. G. et al. Adjuvant bevacizumab for melanoma patients at high risk of recurrence: survival analysis of the AVAST-M trial. Annals of oncology : official journal of the european society for medical oncology **29**, 1843‐1852 (2018). <https://doi.org/doi:10.1093/annonc/mdy229>

103	Cybulska-Stopa, B. et al. Results of systemic treatment of cutaneous melanoma in inoperable stage III and IV. Contemp Oncol (Pozn) **16**, 532-545 (2012). <https://doi.org/doi:10.5114/wo.2012.32487>

104	Deckers, E. A. et al. The association between active tumor volume, total lesion glycolysis and levels of S-100B and LDH in stage IV melanoma patients. Eur J Surg Oncol **46**, 2147-2153 (2020). <https://doi.org/doi:10.1016/j.ejso.2020.07.011>

105	Decoster, L. et al. Biomarker Analysis in a Phase II Study of Sunitinib in Patients with Advanced Melanoma. Anticancer Res **35**, 6893-6899 (2015). <https://doi.org/doi>:

106	Domingo-Domènech, J. et al. Prognostic implications of protein S-100beta serum levels in the clinical outcome of high-risk melanoma patients. Tumour Biol **28**, 264-272 (2007). <https://doi.org/10.1159/000110424>

107	Egberts, F. et al. Long-term survival analysis in metastatic melanoma: serum S100B is an independent prognostic marker and superior to LDH. Onkologie **31**, 380-384 (2008). <https://doi.org/10.1159/000135492>

108	Fang, S. et al. Association of Vitamin D Levels With Outcome in Patients With Melanoma After Adjustment For C-Reactive Protein. J Clin Oncol **34**, 1741-1747 (2016). <https://doi.org/doi:10.1200/jco.2015.64.1357>

109	Faries, M. B., Gupta, R. K., Ye, X., Hsueh, E. C. & Morton, D. L. Melanoma-inhibiting activity assay predicts survival in patients receiving a therapeutic cancer vaccine after complete resection of American Joint Committee on Cancer Stage III Melanoma. Ann Surg Oncol **11**, 85-93 (2004). <https://doi.org/doi:10.1007/bf02524351>

110	Felix, J. et al. Relevance of serum biomarkers associated with melanoma during follow-up of anti-CTLA-4 immunotherapy. Int Immunopharmacol **40**, 466-473 (2016). <https://doi.org/doi:10.1016/j.intimp.2016.09.030>

111	Filia, A. et al. Plasma osteopontin concentrations in patients with cutaneous melanoma. Oncol Rep **30**, 1575-1580 (2013). <https://doi.org/doi:10.3892/or.2013.2666>

112	Geukes Foppen, M. H. et al. Targeted treatment and immunotherapy in leptomeningeal metastases from melanoma. Ann Oncol **27**, 1138-1142 (2016). <https://doi.org/10.1093/annonc/mdw134>

113	Forschner, A. et al. Circulating tumor DNA correlates with outcome in metastatic melanoma treated by braf and mek inhibitors – results of a prospective biomarker study. OncoTargets and Therapy **13**, 5017-5032 (2020). <https://doi.org/doi:10.2147/OTT.S248237>

114	Fusi, A. et al. Circulating melanoma cells and distant metastasis-free survival in stage III melanoma patients with or without adjuvant interferon treatment (EORTC 18991 side study). European journal of cancer (Oxford, England : 1990) **45**, 3189‐3197 (2009). <https://doi.org/doi:10.1016/j.ejca.2009.09.004>

115	Guida, M. et al. Fibrinogen: a novel predictor of responsiveness in metastatic melanoma patients treated with bio-chemotherapy: IMI (Italian melanoma inter-group) trial. Journal of translational medicine **1** (2003). <https://doi.org/doi:10.1186/1479-5876-1-13>

116	Guida, M. et al. Basal level and behaviour of cytokines in a randomized outpatient trial comparing chemotherapy and biochemotherapy in metastatic melanoma. Melanoma research **16**, 317‐323 (2006). <https://doi.org/doi:10.1097/01.cmr.0000200491.00841.5f>

117	Henry, L. et al. Diagnostic value and prognostic significance of plasmatic proteasome level in patients with melanoma. Exp Dermatol **19**, 1054-1059 (2010). <https://doi.org/10.1111/j.1600-0625.2010.01151.x>

118	Hofmann, M. A. et al. Impact of lymph node metastases on serum level of melanoma inhibitory activity in stage III melanoma patients. J Dermatol **38**, 880-886 (2011). <https://doi.org/doi:10.1111/j.1346-8138.2011.01219.x>

119	Hoshimoto, S. et al. Association between circulating tumor cells and prognosis in patients with stage III melanoma with sentinel lymph node metastasis in a phase III international multicenter trial. Journal of clinical oncology **30**, 3819‐3826 (2012). <https://doi.org/doi:10.1200/JCO.2011.40.0887>

120	Hu, H. P., Archer, C., Yip, D. & Peters, G. Clinical predictors of survival in real world practice in stage IV melanoma. Cancer Rep (Hoboken) **6**, e1691 (2023). <https://doi.org/doi:10.1002/cnr2.1691>

121	Iivanainen, S., Ahvonen, J., Knuuttila, A., Tiainen, S. & Koivunen, J. P. Elevated CRP levels indicate poor progression-free and overall survival on cancer patients treated with PD-1 inhibitors. ESMO Open **4**, e000531 (2019). <https://doi.org/doi:10.1136/esmoopen-2019-000531>

122	van Not, O. J. et al. A Survival Tree of Advanced Melanoma Patients with Brain Metastases Treated with Immune Checkpoint Inhibitors. Cancers (Basel) **15** (2023). <https://doi.org/doi:10.3390/cancers15112922>

123	Keller, L. et al. Early Circulating Tumour DNA Variations Predict Tumour Response in Melanoma Patients Treated with Immunotherapy. Acta Derm Venereol **99**, 206-210 (2019). <https://doi.org/doi:10.2340/00015555-3080>

124	Khoja, L. et al. Biomarker utility of circulating tumor cells in metastatic cutaneous melanoma. J Invest Dermatol **133**, 1582-1590 (2013). <https://doi.org/doi:10.1038/jid.2012.468>

125	Koyanagi, K. et al. Serial monitoring of circulating tumor cells predicts outcome of induction biochemotherapy plus maintenance biotherapy for metastatic melanoma. Clin Cancer Res **16**, 2402-2408 (2010). <https://doi.org/doi:10.1158/1078-0432.Ccr-10-0037>

126	Kruijff, S. et al. S-100B concentrations predict disease-free survival in stage III melanoma patients. Ann Surg Oncol **16**, 3455-3462 (2009). <https://doi.org/10.1245/s10434-009-0629-8>

127	Lee, J. H. et al. Pre-operative ctDNA predicts survival in high-risk stage III cutaneous melanoma patients. Ann Oncol **30**, 815-822 (2019). <https://doi.org/doi:10.1093/annonc/mdz075>

128	Levati, L. et al. Circulating miR-1246 and miR-485-3p as Promising Biomarkers of Clinical Response and Outcome in Melanoma Patients Treated with Targeted Therapy. Cancers (Basel) **14** (2022). <https://doi.org/10.3390/cancers14153706>

129	Li, P., He, Q. Y., Luo, C. Q. & Qian, L. Y. Circulating miR-221 expression level and prognosis of cutaneous malignant melanoma. Med Sci Monit **20**, 2472-2477 (2014). <https://doi.org/doi:10.12659/msm.891327>

130	Li, J., Fu, W., Zhang, W. & Li, P. High Number of Circulating Tumor Cells Predicts Poor Survival of Cutaneous Melanoma Patients in China. Med Sci Monit **24**, 324-331 (2018). <https://doi.org/doi:10.12659/msm.904770>

131	Li, C. et al. The value of melanoma inhibitory activity and LDH with melanoma patients in a Chinese population. Medicine (Baltimore) **100**, e24840 (2021). <https://doi.org/doi:10.1097/md.0000000000024840>

132	Lin, S. Y. et al. Multiplex Gene Profiling of Cell-Free DNA in Patients With Metastatic Melanoma for Monitoring Disease. JCO Precis Oncol **2** (2018). <https://doi.org/doi:10.1200/po.17.00225>

133	Lu, L. et al. Dynamic changes of circulating soluble PD-1/PD-L1 and its association with patient survival in immune checkpoint blockade-treated melanoma. Int Immunopharmacol **118**, 110092 (2023). <https://doi.org/doi:10.1016/j.intimp.2023.110092>

134	Lucci, A. et al. Circulating Tumor Cells and Early Relapse in Node-positive Melanoma. Clin Cancer Res **26**, 1886-1895 (2020). <https://doi.org/doi:10.1158/1078-0432.Ccr-19-2670>

135	Lucci, A. et al. Presence of Circulating Tumor Cells Predates Imaging Detection of Relapse in Patients with Stage III Melanoma. Cancers (Basel) **15** (2023). <https://doi.org/doi:10.3390/cancers15143630>

136	Staudt, M. et al. Determinants of survival in patients with brain metastases from cutaneous melanoma. Br J Cancer **102**, 1213-1218 (2010). <https://doi.org/doi:10.1038/sj.bjc.6605622>

137	Sanmamed, M. F. et al. Changes in serum interleukin-8 (IL-8) levels reflect and predict response to anti-PD-1 treatment in melanoma and non-small-cell lung cancer patients. Ann Oncol **28**, 1988-1995 (2017). <https://doi.org/doi:10.1093/annonc/mdx190>

138	Mahalingam, D. et al. Phase II study evaluating the efficacy, safety, and pharmacodynamic correlative study of dual antiangiogenic inhibition using bevacizumab in combination with sorafenib in patients with advanced malignant melanoma. Cancer Chemother Pharmacol **74**, 77-84 (2014). <https://doi.org/doi:10.1007/s00280-014-2479-8>

139	Meral, R. et al. Prognostic significance of melanoma inhibiting activity levels in malignant melanoma. Melanoma Res **11**, 627-632 (2001). <https://doi.org/doi:10.1097/00008390-200112000-00009>

140	Pelizzari, G. et al. Estimating survival in patients with melanoma brain metastases: prognostic value of lactate dehydrogenase. Melanoma Res **33**, 398-405 (2023). <https://doi.org/10.1097/cmr.0000000000000907>

141	Prasmickaite, L. et al. Evaluation of serum osteopontin level and gene polymorphism as biomarkers: analyses from the Nordic Adjuvant Interferon alpha Melanoma trial. Cancer Immunol Immunother **64**, 769-776 (2015). <https://doi.org/doi:10.1007/s00262-015-1686-4>

142	Rao, C. et al. Circulating melanoma cells and survival in metastatic melanoma. International Journal of Oncology **38**, 755-760 (2011). <https://doi.org/doi:10.3892/ijo.2011.896>

143	Diem, S. et al. Serum lactate dehydrogenase as an early marker for outcome in patients treated with anti-PD-1 therapy in metastatic melanoma. Br J Cancer **114**, 256-261 (2016). <https://doi.org/10.1038/bjc.2015.467>

144	Hall, C. S. et al. Circulating Tumor Cells in Stage IV Melanoma Patients. J Am Coll Surg **227**, 116-124 (2018). <https://doi.org/10.1016/j.jamcollsurg.2018.04.026>

145	Sanmamed, M. F. et al. Relevance of MIA and S100 serum tumor markers to monitor BRAF inhibitor therapy in metastatic melanoma patients. Clin Chim Acta **429**, 168-174 (2014). <https://doi.org/10.1016/j.cca.2013.11.034>

146	Sanmamed, M. F. et al. Quantitative cell-free circulating BRAFV600E mutation analysis by use of droplet digital PCR in the follow-up of patients with melanoma being treated with BRAF inhibitors. Clin Chem **61**, 297-304 (2015). <https://doi.org/doi:10.1373/clinchem.2014.230235>

147	Schildbach, V. A. S. et al. C-Reactive Protein and Lymphocyte-to-Monocyte Ratio Predict Recurrence in Stage III Melanoma Patients with Microscopic Sentinel Lymph Node Metastasis. Cancers (Basel) **15** (2023). <https://doi.org/doi:10.3390/cancers15030702>

148	Schmidt, H. et al. Serum YKL-40 predicts relapse-free and overall survival in patients with American Joint Committee on Cancer stage I and II melanoma. J Clin Oncol **24**, 798-804 (2006). <https://doi.org/doi:10.1200/jco.2005.03.7960>

149	Schneider, M. et al. Prognostic Value of Preoperative Inflammatory Markers in Melanoma Patients with Brain Metastases. J Clin Med **10** (2021). <https://doi.org/doi:10.3390/jcm10040634>

150	Schouwenburg, M. G. et al. Vemurafenib in BRAF-mutant metastatic melanoma patients in real-world clinical practice: prognostic factors associated with clinical outcomes. Melanoma Res **28**, 326-332 (2018). <https://doi.org/doi:10.1097/cmr.0000000000000453>

151	Seetharamu, N. et al. Enrollment in clinical trials correlates with improved survival in metastatic melanoma. Oncology **81**, 403-409 (2011). <https://doi.org/doi:10.1159/000335000>

152	Shah, K. P., Song, H., Ye, F. & Johnson, D. B. Prognostic Clinical and Radiographic Biomarkers for BRAF-Targeted Therapy in Advanced Melanoma. Oncologist **26**, e333-e335 (2021). <https://doi.org/doi:10.1002/onco.13562>

153	Silva, S. et al. Genome-Wide Analysis of Circulating Cell-Free DNA Copy Number Detects Active Melanoma and Predicts Survival. Clin Chem **64**, 1338-1346 (2018). <https://doi.org/doi:10.1373/clinchem.2018.290023>

154	Sivan, S. et al. Serum CEACAM1 Correlates with Disease Progression and Survival in Malignant Melanoma Patients. Clin Dev Immunol **2012**, 290536 (2012). <https://doi.org/doi:10.1155/2012/290536>

155	Smit, L. H. et al. Normal values of serum S-100B predict prolonged survival for stage IV melanoma patients. Eur J Cancer **41**, 386-392 (2005). <https://doi.org/10.1016/j.ejca.2004.10.019>

156	Tan, L. et al. Prediction and monitoring of relapse in stage III melanoma using circulating tumor DNA. Ann Oncol **30**, 804-814 (2019). <https://doi.org/doi:10.1093/annonc/mdz048>

157	Tarhini, A. A., Stuckert, J., Lee, S., Sander, C. & Kirkwood, J. M. Prognostic significance of serum S100B protein in high-risk surgically resected melanoma patients participating in Intergroup Trial ECOG 1694. J Clin Oncol **27**, 38-44 (2009). <https://doi.org/doi:10.1200/jco.2008.17.1777>

158	Tartour, E. et al. Predictors of clinical response to interleukin-2--based immunotherapy in melanoma patients: a French multiinstitutional study. J Clin Oncol **14**, 1697-1703 (1996). <https://doi.org/doi:10.1200/jco.1996.14.5.1697>

159	Tas, F. et al. The value of serum levels of IL-6, TNF-alpha, and erythropoietin in metastatic malignant melanoma: serum IL-6 level is a valuable prognostic factor at least as serum LDH in advanced melanoma. Med Oncol **22**, 241-246 (2005). <https://doi.org/doi:10.1385/mo:22:3:241>

160	Tawbi, H. A. et al. Spartalizumab or placebo in combination with dabrafenib and trametinib in patients with BRAF V600-mutant melanoma: exploratory biomarker analyses from a randomized phase 3 trial (COMBI-i). Journal for immunotherapy of cancer **10** (2022). <https://doi.org/doi:10.1136/jitc-2021-004226>

161	Ugurel, S., Rappl, G., Tilgen, W. & Reinhold, U. Increased serum concentration of angiogenic factors in malignant melanoma patients correlates with tumor progression and survival. J Clin Oncol **19**, 577-583 (2001). <https://doi.org/doi:10.1200/jco.2001.19.2.577>

162	Umemura, H. et al. Usefulness of serum 5-S-cysteinyl-dopa as a biomarker for predicting prognosis and detecting relapse in patients with advanced stage malignant melanoma. J Dermatol **44**, 449-454 (2017). <https://doi.org/doi:10.1111/1346-8138.13651>

163	Wilgenhof, S. et al. Single-center experience with ipilimumab in an expanded access program for patients with pretreated advanced melanoma. J Immunother **36**, 215-222 (2013). <https://doi.org/doi:10.1097/CJI.0b013e31828eed39>

164	Wilson, M. A. et al. Impact of initial stage on metastatic melanoma survival. Melanoma Res **29**, 281-288 (2019). <https://doi.org/doi:10.1097/cmr.0000000000000526>

165	Wu, X. et al. Angiopoietin-2 as a Biomarker and Target for Immune Checkpoint Therapy. Cancer Immunol Res **5**, 17-28 (2017). <https://doi.org/doi:10.1158/2326-6066.Cir-16-0206>

166	Wu, X. et al. Anti-CTLA-4 based therapy elicits humoral immunity to galectin-3 in patients with metastatic melanoma. Oncoimmunology **7**, e1440930 (2018). <https://doi.org/doi:10.1080/2162402x.2018.1440930>
